# QTL mapping of yield components and kernel traits in wheat cultivars TAM 112 and Duster

**DOI:** 10.3389/fpls.2022.1057701

**Published:** 2022-12-07

**Authors:** Zhen Wang, Smit Dhakal, Mustafa Cerit, Shichen Wang, Yahya Rauf, Shuhao Yu, Frank Maulana, Wangqi Huang, Joshua D. Anderson, Xue-Feng Ma, Jackie C. Rudd, Amir M. H. Ibrahim, Qingwu Xue, Dirk B. Hays, Amy Bernardo, Paul St. Amand, Guihua Bai, Jason Baker, Shannon Baker, Shuyu Liu

**Affiliations:** ^1^ Texas A&M AgriLife Research and Extension Center, Amarillo, TX, United States; ^2^ Genomics and Bioinformatics Service Center, Texas A&M AgriLife Research, College Station, TX, United States; ^3^ Noble Research Institute, Ardmore, OK, United States; ^4^ Department of Soil and Crop Science, Texas A&M University, College Station, TX, United States; ^5^ Central Small Grain Genotyping Lab and Hard Winter Wheat Genetics Research Unit, U.S. Department of Agriculture-Agricultural Research Service, Manhattan, KS, United States

**Keywords:** wheat, QTL mapping, multiple-environment trials, yield components, kernel traits

## Abstract

In the Southern Great Plains, wheat cultivars have been selected for a combination of outstanding yield and drought tolerance as a long-term breeding goal. To understand the underlying genetic mechanisms, this study aimed to dissect the quantitative trait loci (QTL) associated with yield components and kernel traits in two wheat cultivars `TAM 112' and `Duster' under both irrigated and dryland environments. A set of 182 recombined inbred lines (RIL) derived from the cross of TAM 112/Duster were planted in 13 diverse environments for evaluation of 18 yield and kernel related traits. High-density genetic linkage map was constructed using 5,081 single nucleotide polymorphisms (SNPs) from genotyping-by-sequencing (GBS). QTL mapping analysis detected 134 QTL regions on all 21 wheat chromosomes, including 30 pleiotropic QTL regions and 21 consistent QTL regions, with 10 QTL regions in common. Three major pleiotropic QTL on the short arms of chromosomes 2B (57.5 - 61.6 Mbps), 2D (37.1 - 38.7 Mbps), and 7D (66.0 - 69.2 Mbps) colocalized with genes *Ppd-B1*, *Ppd-D1*, and *FT-D1*, respectively. And four consistent QTL associated with kernel length (KLEN), thousand kernel weight (TKW), plot grain yield (YLD), and kernel spike^-1^ (KPS) (*Qklen.tamu.1A.325*, *Qtkw.tamu.2B.137*, *Qyld.tamu.2D.3*, and *Qkps.tamu.6A.113*) explained more than 5% of the phenotypic variation. QTL Qklen.tamu.1A.325 is a novel QTL with consistent effects under all tested environments. Marker haplotype analysis indicated the QTL combinations significantly increased yield and kernel traits. QTL and the linked markers identified in this study will facilitate future marker-assisted selection (MAS) for pyramiding the favorable alleles and QTL map-based cloning.

## Introduction

Bread wheat (*Triticum aestivum* L.) is one of the most common staple foods in the human daily diet, providing calories and proteins for one-third of the world population (Shewry and Hey, 2015). By the end of this century, the projected world population is expected to be 10.9 billion, leading to a significantly higher wheat consumption forecast (Enghiad et al., 2017; Roser et al., 2019). Additionally, droughts, diseases and pest prevalence increase concern about insufficient wheat production (Yue et al., 2019). To meet future demands, novel wheat cultivars with higher yield potential under stressed environments need to be developed (Trethowan and Mujeeb-Kazi, 2008).

Yield is a complex quantitative trait involving multiple QTL, epistatic, and genetic by environment interactions. Multiple environment QTL mapping analysis can reveal the genetic architecture and QTL stability, which could facilitate the effective utilization of genetic resources in breeding by marker-assisted selection (MAS) (Collard and Mackill, 2008). Yield consists of three main components, kernels spike^-1^ (KPS), spikes m^-2^ (SPM), and thousand kernel weight (TKW) (Cao et al., 2020). Most yield-related QTL may influence one or multiple yield components (Mason et al., 2013; Yu et al., 2018; Juliana et al., 2019; Li et al., 2019; Yang et al., 2020). Analysis of wheat breeding during practices from the past several decades show that yield improvement was mainly due to the continuous gains in KPS and SPM (Feng et al., 2018; Würschum et al., 2018). QTL for KPS have been reported on all wheat chromosomes from various sources (Assanga et al., 2017; Li et al., 2018; Liu et al., 2018; Yu et al., 2018; Muhammad et al., 2020; Pang et al., 2020). Several genes have been well-characterized and known for controlling KPS. For example, *FLOWERING LOCUS T1* gene (*TaFT1*) on chromosome group seven, also known as *VRN3*, which controls KPS, heading date (HD), as well as the integrator of several autonomous pathways (Yan et al., 2006; Lv et al., 2014). Deletion of *FT-B1* or *FT-D1* in hexaploid wheat increased the KPS and delayed the HD (Finnegan et al., 2018; Shaw et al., 2019; Glenn et al., 2021; Chen et al., 2022). Functional *WHEAT ORTHOLOG of APO1* (*WAPO1*) gene on chromosome group seven also increased KPS in wheat (Ikeda et al., 2007; Kuzay et al., 2019; Kuzay et al., 2022). For SPM, QTL have been identified on all wheat chromosomes (Assanga et al., 2017; Li et al., 2018; Ma et al., 2018; Yu et al., 2018; Li et al., 2020; Pang et al., 2020; Isham et al., 2021; Ren et al., 2021). Several genes associated with SPM have been characterized, including *TaD27* on chromosome group seven encoding a strigolactones biosynthesis enzyme that regulates the tiller number in many plant species, including wheat (Zhao et al., 2020). Additionally, *TaPIL1* on chromosome group five reduced the tiller number by interacting with *TaSPL3/17* and regulating *TaTB1* transcription (Zhang et al., 2021).

Thousand kernel weight is another important yield component, which has intensively selected during domestication and modern breeding (Golan et al., 2015; Qin et al., 2015). Similar to KPS and SPM, TKW QTL have been detected on all wheat chromosomes (Assanga et al., 2017; Sun et al., 2017; Li et al., 2018; Ma et al., 2018; Duan et al., 2020; Muhammad et al., 2020; Pang et al., 2020; Isham et al., 2021; Ren et al., 2021). *TaGL3* (Yang et al., 2019a), *TaGS1* (Guo et al., 2013), *TaGS3* (Yang et al., 2019b), *TaGS5* (Wang et al., 2015), *TaGW2* (Su et al., 2011), *TaGW7* (Wang et al., 2019), *TaGW8* (Yan et al., 2019), *TaTGW6* (Hanif et al., 2016), and *TaTGW7* (Hu et al., 2016), have been characterized for association with kernel size and TKW. Genes with pleiotropic effects on multiple yield-related traits of wheat were also reported, such as *Ppd1*, responsible for photoperiod and HD regulation. The copy number variations of *Ppd-B1* facilitated the adaption of wheat worldwide by modifying the HD (Würschum et al., 2015). Besides affecting HD, *Ppd-B1* also showed pleiotropic effects on KPS, SPM, and TKW in durum wheat (Arjona et al., 2018).

Although abundant QTL were characterized in previous studies, only a few were used in wheat breeding since the majority of them showed inconsistent or minor effects in different genetic backgrounds (Zheng et al., 2021). To avoid the unfavorable impact on QTL deployment, and concurrently meet the breeding purposes for the Southern Great Plains, we used the two most drought tolerant cultivars ‘TAM 112’ and ‘Duster’ in Texas and Oklahoma, respectively, to construct a mapping population. TAM 112 and Duster significantly differed in TKW, KPS, and kernel shape while both maintained outstanding yield performance in the Southern Great Plains region, where the extreme drought stresses frequently threaten the wheat production. In order to provide insight into the genomic regions contributing to high yield under water-limited conditions, this study evaluated the yield and agronomic traits of the mapping population in diverse environments. The consistent and pleiotropic QTL were identified using single nucleotide polymorphisms (SNPs), and their effects were assessed through haplotype analysis.

## Material and methods

### Plant material

The mapping population contains 182 F_6_ recombinant inbred lines (RILs) derived from the cross between ‘TAM 112’ (PI 643143) and ‘Duster’ (PI 644016). TAM 112 is an awned, red glume, medium-early maturing, semidwarf hard red winter wheat released by Texas A&M AgriLife Research in 2005 (Rudd et al., 2014). Carrying *1AL.1RS* chromosome translocation from rye (*Secale cereale* L), TAM 112 has greenbug [*Schizaphis graminum* (Rondani)] and wheat curl mite (*Aceria tosichella* Keifer) resistance, also the excellent grain yield potential, end-use quality, and drought tolerance. Duster is an awned, white glume, intermediate heading time, dual-purpose, semidwarf hard red winter wheat cultivar released by the Oklahoma Agricultural Experiment Station and the USDA-ARS in 2006 (Edwards et al., 2012). It shows resistance to *Soilborne wheat mosaic virus* (SBWMV), *Wheat spindle streak mosaic virus* (WSSMV), and Hessian fly (*Mayetiola destructor* Say).

### Field experiments

The RIL population and both parents were planted under different irrigation regimes at seven locations across Texas and Oklahoma from 2017 to 2019 with a total of 13 environments (combination of treatments, years, and locations) (**Table S1**). The four Texas dryland environments are Bushland middle school (35° 06’ N, 102° 27’ W) in 2017, 2018 and 2019 (17BMS, 18BMS, 19BMS), and Chillicothe (34° 07’ N, 99° 18’ W) in 2017 (17CH). Six Texas irrigated environments are Texas A&M AgriLife Research stations in Bushland (35° 06’ N, 102° 27’ W) in 2018 (18BI), Bushland south pivot (35° 06’ N, 102° 27’ W) in 2017, 2018 and 2019 (17BSP100, 18BSP100, 19BSP100 at full irrigation, and 17BSP67 at 67% irrigation level) and Dumas (35° 51’ N, 101° 58’ W) in 2018 (18DMS). The three Oklahoma dryland environments include Noble Foundation Dupy Farm (34.29° N, 96.99° W) in 2018 (18NFD) and Red River Research and Demonstration Farm (33.88°N, 97.28°W) in 2017 and 2019 (17RRD, 19RRD).

The field experiments employed an Alpha Lattice design with incomplete blocks each containing five plots. All individual environments had two replications, except one replication for 18DMS and three replications for 18NFD and 19RRD. The plot size of all Oklahoma locations was 2.32 m^2^. However, for Texas locations, the plot size was 4.64 m^2^ for irrigated land and 6.96 m^2^ for dryland. Standard management was employed for all field trials (Assanga et al., 2017).

### Phenotyping and statistical analysis

A set of 18 traits from 13 individual environments (**Table S1**) were collected following the same procedures as described by Assanga et al. (2017). After maturity, a biomass sample was collected by manually harvesting a 0.5-meter-long evenly growing inner row in each plot, and the rest of plots were harvested by a combine harvester. The combine-harvested seeds were weighed for grain yield (YLD) and test weight (TW). Biomass samples were dried in a drying chamber for more than 72 h at 60°C and then weighed to calculate total biomass weight (BM). Spikes from each biomass sample were counted, weighed, and converted to SPM and single head dry weight (SHDW). The spike length (SL) was measured in 17BSP100 and 18BI. All spikes were threshed to get seeds and weighed to calculate the biomass yield (BMYLD).

The threshed seeds of biomass samples from 18BSP100, 18DMS, 18BI, 18BMS, 19BSP100, and 19BMS were further scanned for kernel traits with an HP G4010 (HP 11956A, Hewlett-Packard, Plao Alto, CA, USA) scanner, and seed weights of the scanned seeds were recorded. The scan resolution was 300 dots per inch (DPI). Seed images were analyzed using grain scan v1.0 software (Whan et al., 2014) with parameters set as minimum grain width = 20, minimum grain length = 20, and auto threshold sensitivity = 0.8. Kernel area (AREA), kernel perimeter (PERI), kernel length (KLEN), kernel width (KWID), and kernel number were automatically output from the software. Two methods were employed to estimate TKW: dividing the scanned grain weight by kernel number and multiplying by 1000 in these locations using the seed scanner, and by directly hand-counting and weighing 200 threshed seeds to estimate TKW for the rest of environments. Kernels spike^-1^ was calculated using BMYLD, TKW, and SPM. Single culm weight (SCW) was computed from BM divided by SPM. Harvest Index (HI) was calculated using BMYLD divided by BM. Single head dry grain weight (SHGW) was computed using BMYLD and the number of spikes.

The raw phenotypic data was first filtered manually to remove the outliers that were beyond three standard deviations from the mean. Mega-environments were classified based on the principal component analysis (PCA). Analysis of variance (ANOVA) was conducted to estimate the significance of genotypes (G), environments (E), and genotype-by-environment interactions (G x E). The best linear unbiased estimate (BLUE) values were calculated for all phenotypic data by fitting the mixed linear model *Y_ijkl_ = μ+Env_i_+Rep_j_(Env_i_)+Block_k_(Env_i_ Rep_i_)+Gen_l_+Env_i_xGenl+ϵ_ijkl_*, where genotypes and environments were fixed and random effects, respectively, under both individual environments and mega-environments. In the mixed linear model, *Y* represents the phenotype, *Env*, *Rep*, *Block*, and *ϵ* are random effects that accounted for the effects of environment, replication, block, and error, respectively. *Gen* is fixed genotypic effect. The broad-sense heritability was calculated on entry-mean basis using the following formula:


$${\text{H}}^{2}=\frac{\sigma_{\text{g}}^{2}}{\sigma_{\text{g}}^{2}+\sigma_{\text{ge}}^{2}/nLoc+\sigma_{\text{g}}^{2}/(nLoc\times nRep)}$$

where $\sigma_{\text{g}}^{2}$ , $\sigma_{\text{ge}}^{2}$ , and $\sigma_{\text{g}}^{2}$ , are the estimated variances of genotype, genotype-by-environment, and error, while nLoc and nRep indicate the number of environments and replications, respectively. The Multiple Environment Trait Analysis with R (META-R) software (Alvarado et al., 2020) based on the R package lme4 (Bates et al., 2015) was used for PCA, ANOVA, and heritability analysis. Removal of outliers from the raw data as well as BLUE based Pearson correlation coefficients among variables were analyzed by JMP pro 16 (Sall et al., 2017).

### Genotyping and linkage map construction

DNA of parents and 182 RILs was extracted from the young leaves using the modified cetyltrimethylammonium bromide (CTAB) protocol at 2 to 3 leaf stage (Liu et al., 2014). The quality of the extracted DNA was evaluated using agarose gel electrophoresis. About 200 ng genomic DNA per sample was used for genotyping-by-sequence (GBS) library construction in the USDA Central Small Grain Genotyping Center (CSGGC), Manhattan, KS (https://hwwgenotyping.ksu.edu/genotyping.html) following the protocol from Poland and Rife (2012). In brief, normalized genomic DNA was double-digested using restriction enzymes *PstI* and *MspI* (New England Biolabs Inc., Ipswich, MA) and ligated to a set of barcode adapters using T4 ligase (New England Biolabs Inc). The GBS library was size-selected for 200-300 bp fragments in an E-gel system (ThermoFisher Scientific). The selected polymerase chain reaction (PCR) fragments were purified with the GenCatch PCR purification kit (Epoch Life Science Inc., Sugar Land, TX), and quantified with the Qubit dsDNA HS assay kit (ThermoFisher Scientific) before sequencing using PI v3 chips and Hi-Q sequencing kits (ThermoFisher Scientific) in an Ion Torrent Proton sequencer (Life Technologies, Carlsbad, CA). The raw sequence reads were assigned to each sample based on attached barcodes, then trimmed to 64 bp DNA sequences including the 5’ restriction site. SNPs were detected using a reference-based pipeline in TASSEL 5.0 (Bradbury et al., 2007) using the IWGSC RefSeq v1.1 genome (IWGSC et al., 2018) to assign the physical positions for each SNP.

The homozygous SNP genotypes of each RIL were converted to parental genotypes for ‘A’ as the female parent TAM 112 and ‘B’ as the male parent Duster. The heterozygous SNPs were considered missing values. SNPs with no calls (NN) from parental genotypes and missing value ratio of more than 20%, or A/B ratio outside of 0.5 to 2 were removed before linkage map construction. Filtered GBS data was inspected, and the false double crossovers were manually removed according to the physical location of the SNPs. Redundant and distorted makers were purged by JoinMap 4.0 software (Van Ooijen, 2006). Only one SNP was retained for linkage map construction when multiple SNPs have more than 99.5% similarity. SNPs significantly deviated from 1:1 Mendelian segregation ratios (*P*< 0.01) were also removed for the chi-square (χ^2^) test. Finally, the remaining unique SNPs were grouped by setting independent logarithm of odds (LOD) threshold ranging from 3 to 30, with maximum likelihood (ML) as the mapping algorithm and Kosambi’s regression mapping (Kosambi, 2016) as mapping function to construct the linkage map.

### QTL and haplotype analysis

BLUE values from individual and mega-environments were used for QTL analysis using the inclusive composite interval mapping (ICIM) function in QTL IciMapping v 4.1 (Meng et al., 2015). Additive and additive by environment interaction (A x E) effects of QTL based on single allele were determined by ICIM additive mapping (ICIM-ADD) function. The threshold LOD score to declare a significant QTL with a type I error less than 0.05 was determined using one thousand permutations. The step length in QTL scanning was one centimorgan (cM), and the probability in stepwise regression (PIN) was 0.001.

The epistasis effects were analyzed using the epistatic mapping (ICIM-EPI) function. The threshold LOD score was 10 for reducing interaction number, step length was 5 cM, and PIN was 0.0001. QTL names were designated following procedure described by McIntosh et al. (2013) with slight modifications in the format as: *Qtrait.tamu.chrom.Mbps*, where ‘Q’ represents QTL, ‘trait’ is the trait name, ‘tamu’ is for the ‘Texas A&M University’, ‘chrom’ represent chromosome number, and ‘Mbps’ is the physical position aligned to the IWGSC Chinese Spring RefSeq v 1.0 (https://wheat-urgi.versailles.inra.fr/Seq-Repository/Assemblies). If multiple QTL were mapped in the same genomic region with overlapping flanking intervals, they were considered to belong to the same QTL region. A ‘consistent QTL’ was defined as the QTL mapped on the same physical position for the same trait that was significant in at least two individual environments. While ‘pleiotropic QTL’ was defined as a QTL mapped on the same physical position for different traits that were not highly correlated.

For each trait, the allelic effects of a QTL were estimated using the average trait BLUE values based on marker haplotype groups. The single peak marker which explained the maximum phenotypic variation was used to distinguish the allele source. RILs were grouped based on marker haplotypes for five traits, including YLD, KPS, TKW, KLEN, and KWID. The RILs carrying different alleles were analyzed based on their allelic combinations in the same individual environment. For statistical analysis, Tukey’s honest significance difference (HSD) test was conducted in JMP pro 16 (Sall et al., 2017) to compare the trait BLUE values among haplotype groups at *P*< 0.05.

## Results

### Phenotype and heritability

Distributions of all traits across testing environments were depicted using boxplots (**Figure S1**). Grain yield averages ranged from 115.8 to 564.2 g m^-2^ while BM averages ranged from 964.7 to 2331.0 g m^-2^. Biomass yield averages ranged from 312.3 to 705.8 g m^-2^. The ranges of HI and TW were 16.3 to 39.5% and 706.0 to 794.8 Kg m^-3^. The three yield components, TKW, SPM, and KPS, had average values ranging from 22.2 to 32.6 g, 534.5 to 1124.9 spikes m^-2^, and 17.1 to 35.1 kernel spike^-2^ respectively. For means of kernel traits, KLEN ranged from 4.7 to 5.4 mm and KWID ranged from 2.3 to 2.7 mm. In addition, average HD ranged from 112.4 to 134.4 days while average PH ranged from 54.3 to 84.0 cm.

The variance of genotype, environment, and G x E for all traits were significant (*p<* 0.001), except the variance of genotype for BM and G x E for SPM (**Table S2**). TAM 112 had higher values of BM, SHDW, SHGW, TKW and all four kernel traits, while Duster had higher values of KPS, YLD, HD and PH in the mean performance as well as the overall BLUE values across all individual environments. No significant difference was observed for BMYLD, KPS, SPM, HI, TW, and PH between the two parents. The entry mean based broad-sense heritability was moderate to high for all traits (0.51 – 0.95), except BM (0.10) (**Table S2**).

### Correlations among traits

Significant positive correlations were observed between BMYLD and SPM (*r* = 0.43 – 0.77, *p*< 0.001), TKW (*r* = 0.21 – 0.46, *p*< 0.01), KPS (*r* = 0.26 – 0.59, *p*< 0.001) in most environments (**Table S3**). Consistent negative correlations were observed between KPS and TKW (*r* = -0.22 – -0.51, *p*< 0.01) and between SPM and TKW (*r* = -0.22 – -0.35, *p*< 0.01). However, the correlation between KPS and SPM was not significant (*r* = -0.18 – 0.04) in most of the environments, suggesting that KPS and SPM can be improved simultaneously. Both TW (*r* = 0.27 – 0.52, *p*< 0.001) and HI (*r* = 0.16 – 0.66, *p*< 0.001) were significantly and positively correlated with YLD in all environments, except in 19BMS for TW, and in 17BSP67 and 17BSP100 for HI. Heading date and PH had inconsistent correlations with YLD under individual environments, and the overall correlation value was negative. In addition, moderate to high positive correlations were observed between TKW and all four kernel size traits (*r* = 0.57 – 0.97, *p*< 0.001) from all six environments in 2018 and 2019.

### Mega-environment classification

Mega environments were assigned based on the biplot data for each trait across multiple environments. Only the traits directly related to yield were analyzed for the mega-environment. YLD, SPM, and HI had three mega-environments (ME1 – ME3), while BMYLD, KPS, TKW, KLEN, and KWID had two mega-environments (ME1 – ME2) (**Figure S2** and **Table S4**).

### Genetic map construction

A total of 23,609 SNPs between TAM 112 and Duster were called and 7,139 polymorphic SNPs were retained for linkage map construction after removal of monomorphic makers or markers with missing datapoints >20% or heterozygotes >10%. Furthermore, 712 distorted and 1,346 redundant SNPs were removed from the final map. A total of 5,081 unique SNPs were assigned to 45 linkage groups corresponding to 21 chromosomes. The final map spanned 2,399.5 cM in genetic distance or 11,540.9 mega-base-pairs (Mbps) in physical distance, with an average marker density of 2.12 SNPs cM^-1^ and 0.44 SNPs Mbps^-1^. The SNPs in A, B, and D genomes were 1,517, 2,581, and 983, respectively (**Table S5**). The SNPs density maps that based on genetic and physical positions were shown in **Figure S3**.

### QTL analysis

**Figure 1:**
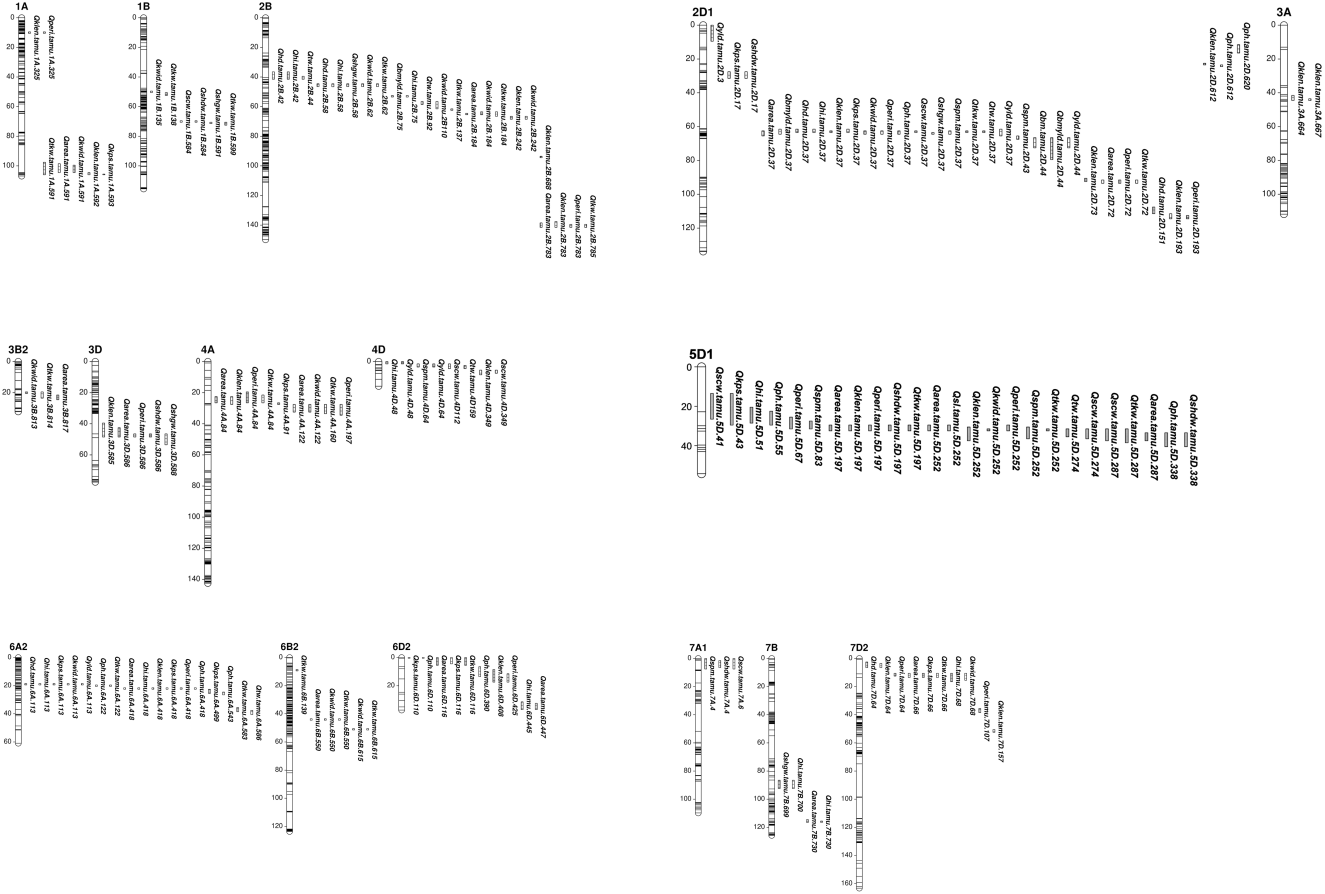
Consistent and pleiotropic QTL for yield related, agronomy and kernel traits identified with TAM112/Duster population by individual and multiple environment analysis. Traits include: 1) grain yield from whole plot (YLD, g m^-2^); 2) dry weight of biomass sample (BM, g m^-2^); 3) grain yield from biomass sample (BMYLD, g m^-2^); 4) test weight (TW, Kg m^-3^); 5) harvest index (HI, %); 6) kernel spike^-1^ (KPS, kernel spike^-1^); 7) spike m^-2^ (SPM, spike m^-2^); 8) thousand kernel weight (TKW, g); 9) single head dry weight (SHDW, mg); 10) single head grain weight (SHGW, mg); 11) single culm weight (SCW, g); 12) kernel area (AREA, mm^2^); 13) kernel perimeter (PERI, mm); 14) kernel length (KLEN, mm); 15) kernel width (KWID, mm); 16) spike length (SL, cm); 17) heading date (HD, days); 18) plant height (PH, cm).

A total of 134 QTL regions were identified by analyzing BLUE values of 16 traits from individual environments, across individual environments, within mega-environment, and across mega-environments (**Figures 1**, **S4, S5** and **Table S6**). Among them, 21 consistent QTL for 12 traits on 10 chromosomes were identified in at least two individual environments (**Table 1**). A set of 30 QTL showing pleiotropic effects to at least two traits without strong correlations were mapped on 12 chromosomes. Ten QTL were in common between consistent and pleiotropic QTL (**Table S6**).

**Table 1:** Consistent and pleiotropic QTL for yield related, agronomy and kernel traits identified with TAM112/Duster population by individual and multiple environment analysis.

QTL name	Chrom	Peak Position (Mb)^a^	Traits^b^	Threshold	LOD^c^	LOD (A)	LOD (AbyE)	PVE (%)^d^	PVE(A) (%)	PVE(AbyE)	Add effect^e^	Parental favorable allele	LeftMarker	Left SNP alleles	TAM 112 alleles	Consistent QTL	Pleiotropic QTL	Environments^f^
Qarea.tamu.2D.37	2D	37.2 - 38.7	AREA	3.4 - 6.1	4.6 - 22.2	1.7 - 11.8	4.8 - 10.5	2.0 - 12.9	0.6 - 4.5	1.3 - 8.0	-0.34 - -0.04	Duster	chr2D_35558557	G/A	G	y	y	18BI, 18BMS, 18DMS, 19BSP100, 19BMS, across 6 envs
Qarea.tamu.2D.72	2D	71.9 - 72.7	AREA	3.4 - 6.1	5.2 - 16.2	14.1	2.0	5.1 - 6.7	5.3	1.3	0.13 - 0.21	TAM 112	chr2D_71764015	C/T	C	y		18BI, 19BSP100, across6 envs
Qarea.tamu.4A.84	4A	83.8 - 90.5	AREA	3.4 - 6.1	4.1 - 14.6	9.2	5.3	5.5 - 6.3	3.4	2.8	-0.24 - -0.1	Duster	chr4A_30316423	G/C	G	y		18DMS, 19BSP100, across 6 envs
Qarea.tamu.5D.197	5D	196.9	AREA	3.4	3.7 - 6.4			7.3 - 8.4			-0.2 - -0.16	Duster	chr5D_70896874	G/A	G	y		18BSP100, 19BMS
Qarea.tamu.5D.252	5D	252.0	AREA	3.4 - 6.1	6.1 - 26.9	21.7	5.2	7.7 - 9.8	8.30	1.50	-0.23 - -0.16	Duster	chr5D_233797558	C/T	C		y	18BI, across 6 envs
Qarea.tamu.6D.116	6D	115.7	AREA	3.4 - 6.1	4.1 - 9.1	7.4	1.7	3.3 - 4.6	2.7	0.6	0.09 - 0.16	TAM 112	chr6D_97915036	G/A	G		y	19BMS, across 6 envs
Qarea.tamu.7D.66	7D	66 - 67.7	AREA	3.4 - 6.1	8.2 - 26.7	16.9	9.8	9.6 - 12	6.2	3.3	0.1 - 0.3	TAM 112	chr7D_64236400	G/T	G	y	y	18BI, 18BMS, across 6 envs
Qbm.tamu.2D.44	2D	43.7	BM	7.7	8.0	0.5	7.5	14.5	1.6	12.9	6.9	TAM 112	chr2D_42872584	A/C	A		y	across 9 envs
Qbmyld.tamu.2D.44	2D	43.7	BMYLD	3.4	3.7			9.2			-16.9	Duster	chr2D_42872584	A/C	A		y	18BSP100
Qbmyld.tamu.2D.37	2D	37.1 - 37.4	BMYLD	3.4 - 7.5	3.8 - 15.4	2.2 - 5.0	3.8 - 13.2	9.0 - 26.5	4.7 - 10.1	2.9 - 21.8	-28.3 - -6.2	Duster	chr2D_35558557	G/A	G	y	y	17BSP67, 17BSP100, ME2, ME12, across 9 envs
Qhd.tamu.2B.42	2B	41.8	HD	3.4 - 6.3	7.3 - 9.5	4.8	4.7	3.7 - 7.6	1.6	2.1	-0.92 - -0.27	Duster	chr2B_35320841	A/T	A		y	18BSP100, across 6 envs
Qhd.tamu.2B.58	2B	57.5	HD	3.4 - 6.3	4.5 - 10.7	4.1	4.9	3.3 - 7.3	1.4	2.0	-1.4 - -0.24	Duster	chr2B_57246450	A/C	A	y	y	17BMS, 18BI, 18BMS, across 6 envs
Qhd.tamu.2D.37	2D	37.4 - 38.7	HD	3.4 - 6.3	20.4 - 82.8	56.0	26.7	22.0 - 39.9	21.3	18.6	0.96 - 2.2	TAM 112	chr2D_35558557	G/A	G	y	y	18BI, 18BMS, 18DMS, 18BSP100, 19BSP100, across 6 envs
Qhd.tamu.6A.113	6A	113.0	HD	3.4 - 6.3	6.5 - 8.8	5.1	3.7	2.6 - 5.3	1.7	0.9	-0.65 - -0.27	Duster	chr6A_122456115	C/T	C		y	18BMS, across 6 envs
Qhd.tamu.7D.64	7D	64.4 - 67.7	HD	3.4 - 6.3	4.4 - 64.5	57.8	6.7	2.9 - 25.3	21.3	2.5	-1.7 - -0.7	Duster	chr7D_64236400	G/T	G	y	y	17BMS, 18BI, 18BMS, 18DMS, 18BSP100, 19BSP100, across 6 envs
Qhi.tamu.2B.42	2B	41.8	HI	3.4 - 4.0	4.2 - 4.5	2.5	2.0	3.7 - 5.9	3.20	0.6	0.59 - 0.86	TAM 112	chr2B_35320841	A/T	A		y	18BSP100, ME3
Qhi.tamu.2B.58	2B	57.5	HI	3.4 - 7.5	4.5 - 8.1	4.1 - 6.3	0.5 - 4.0	5.3 - 11.9	2.1 - 8.1	2.2 - 3.8	0.3 - 1.1	TAM 112	chr2B_57246450	A/C	A		y	17BSP100, ME2, ME123, across 9 envs
Qhi.tamu.2D.37	2D	37.4 - 38.7	HI	3.4 - 7.5	7.4 - 46.7	25.2	21.4	13.4 - 40.7	22.9	17.7	-2.2 - -0.74	Duster	chr2D_35558557	G/A	G	y	y	17BSP67, 18DMS, 18BSP100, 19BSP100, ME2, ME3, ME123, across 9 envs
Qhi.tamu.6A.113	6A	113.0	HI	3.4 - 7.5	4.5 - 10.7	3.0 - 8.0	1.5 - 2.7	6.1 - 12.4	4.0 - 5.9	2.1 - 6.5	0.4 - 0.94	TAM 112	chr6A_122456115	C/T	C		y	18BI, ME1, across 9 envs
Qhi.tamu.7D.68	7D	67.7	HI	3.4 - 7.5	7.9 - 11.8	3.7 - 5.9	4.6 - 5.9	5.5 - 11.6	3.0 - 4.7	2.1 - 2.6	0.3 - 1.2	TAM 112	chr7D_64236400	G/T	G		y	18BSP100, ME3, across 9 envss
Qklen.tamu.1A.325	1A	324.6	KLEN	3.4 - 6.3	6.3 - 63	11.4 - 59.9	0.05 - 5.7	5.7 - 19.5	4.5 - 18.0	0.02 - 1.2	0.05 - 0.10	TAM 112	chr1A_306143327	G/A	G	y		18BI, 18BSP100, 18DMS, 18BMS, 19BSP100, 19BMS, ME1, ME2, ME12, across 6 envs
Qklen.tamu.2B.688	2B	688.4	KLEN	3.4 - 6.3	3.6 - 12.1	4.0 - 8.1	0.4 - 4.0	1.9 - 6.5	1.8 - 4.3	0.3 - 1.0	-0.06 - -0.03	Duster	chr2B_690936220	A/G	A	y		18BMS, 19BMS, ME1, ME2, across 6 envs
Qklen.tamu.2B.783	2B	781.2 - 784.7	KLEN	3.4 - 6.3	5.6 - 22.4	6.1 - 19.8	0.8 - 2.6	3.7 - 6.2	3.2 - 5.2	0.0015 - 0.70	0.04 - 0.07	TAM 112	chr2B_779319734	C/T	C	y		18BMS, 18BSP100, 19BMS, ME1, ME2, ME12, across 6 envs
Qklen.tamu.2D.37	2D	37.4 - 38.7	KLEN	4.1 - 4.2	4.5 - 6.5	4.3 - 6.0	0.2 - 0.5	2.0 - 3.1	1.9 - 2.2	0.04 - 0.86	-0.04 - -0.03	Duster	chr2D_39423582	A/T	A		y	ME2, ME12
Qklen.tamu.3A.664	3A	664 - 667.3	KLEN	3.4 - 6.3	3.8 - 56.5	39.9	16.6	4.0 - 33.6	20.6	13.5	-0.2 - 0.05	Duster	chr3A_659529719	C/T	C	y		18BSP100, 19BMS, 19BSP100, ME2, ME12, across 6 envs
Qklen.tamu.4A.84	4A	83.8 - 90.5	KLEN	3.4 - 6.3	3.9 - 8.4	2.5 - 6.2	0.17 - 4.9	2.1 - 6.6	0.9 - 2.8	0.049 - 1.2	-0.06 - -0.02	Duster	chr4A_30316423	G/C	G	y		18DMS, 19BSP100, ME2, ME12, across 6 envs
Qklen.tamu.5D.252	5D	252.0	KLEN	3.4 - 6.3	4.4 - 11.9	7.7 - 10.9	0.3 - 1.7	3.3 - 6.5	2.6 - 6.5	0.04 - 1.9	-0.056 - -0.034	Duster	chr5D_233797558	C/T	C		y	18BMS, ME1, ME12, across 6 envs
Qklen.tamu.7D.157	7D	156.8	KLEN	3.4 - 6.3	6.1 - 23.9	7.3 - 15.4	0 - 12.6	4.7 - 8.5	1.9 - 7.6	0.16 - 4.1	0.029 - 0.088	TAM 112	chr7D_156192771	A/G	A	y		19BMS, 19BSP100, ME2, ME12, across 6 envs
Qklen.tamu.7D.64	7D	64.4 - 67.7	KLEN	3.4 - 4.1	4.2 - 6.9	4.0 - 5.1	0.2 - 1.3	3.2 - 10.3	1.9 - 3.2	0.8 - 1.3	0.03 - 0.08	TAM 112	chr7D_64236400	G/T	G		y	18BI, ME1, ME12
Qkps.tamu.2D.37	2D	37.4 - 38.7	KPS	3.4 - 7.6	4.3 - 20.8	2.4 - 14	1.4 - 10.9	5.0 - 13.5	2.8 - 10.6	1.7 - 3.4	0.32 - 1.1	TAM 112	chr2D_35558557	G/A	G	y	y	17BSP67, 18BI, 19BMS, ME1, ME2, ME12, across 9 envs
Qkps.tamu.6A.113	6A	113.0	KPS	3.4 - 7.6	3.8 - 17.3	5.8 - 11.8	3.2 - 5.5	7.1 - 11.6	4.7 - 6.7	2.5 - 4.9	0.4 - 1.4	TAM 112	chr6A_122456115	C/T	C	y	y	17BMS, 18BSP100, 19BSP100, ME1, across 9 envs
Qkps.tamu.6D.110	6D	110.4 - 115.7	KPS	3.4 - 7.6	5.4 - 13.9	4.2 - 9.2	0.2 - 4.7	5.3 - 10.9	3.6 - 8.1	0.3 - 2.1	-0.8 - -0.4	Duster	chr6D_97915036	G/A	G		y	19BSP100, ME1, ME12, across 9 envs
Qkps.tamu.7D.66	7D	66.0 - 67.7	KPS	3.4 - 7.6	4.3 - 11.9	5.3 - 8.2	0.6 - 3.8	4.1 - 6.7	3.2 - 5.3	0.4 - 1.2	-0.7 - -0.4	Duster	chr7D_64236400	G/T	G		y	18BI, ME1, ME12, across 9 envs
Qkwid.tamu.2B.62	2B	61.6	KWID	3.4	4.8			8.8			0.04	TAM 112	chr2B_57246450	A/C	A		y	18DMS
Qkwid.tamu.2D.37	2D	37.1 - 37.4	KWID	3.4 - 6.3	5.3 - 9.8	3.1 - 7	0.3 - 6.7	7.3 - 13.1	2.2 - 9.2	0.5 - 5.5	-0.036 - -0.009	Duster	chr2D_35558557	G/A	G	y	y	18BMS, 19BSP100, ME1, ME2, across 6 envs
Qkwid.tamu.5D.252	5D	252.0	KWID	4.1	4.4	3.0	1.4	1.9	1.3	0.5	-0.012	Duster	chr5D_233797558	C/T	C		y	ME12
Qkwid.tamu.6A.113	6A	113.0	KWID	3.4	3.6			4.1			-0.02	Duster	chr6A_122456115	C/T	C		y	18BSP100
Qkwid.tamu.7D.68	7D	62.2 - 66	KWID	3.4 - 6.3	3.5 - 17.2	12.1 - 13.2	0.6 - 4.2	7.6 - 16.2	5.9 - 13	0.8 - 2.3	0.018 - 0.037	TAM 112	chr7D_64236400	G/T	G	y	y	18BI, 18BMS, ME1, ME12, across 6 envs
Qperi.tamu.1A.325	1A	324.6	PERI	3.4 - 6.2	4.5 - 22.8	19.3	3.4	3.1 - 15.7	7.4	2.1	0.13 - 0.21	TAM 112	chr1A_306143327	G/A	G	y		18BI, 18BSP100, 19BMS, across 6 envs
Qperi.tamu.2D.37	2D	37.1 - 38.7	PERI	3.4 - 6.2	4.1 - 14.2	5.6	8.5	4.5 - 9.2	2.1	2.4	-0.02 - -0.07	Duster	chr2D_35558557	G/A	G	y	y	18BMS, 18DMS, 18BMS, 19BSP100, across 6 envs
Qperi.tamu.2D.72	2D	71.9 - 72.7	PERI	3.4 - 6.2	3.6 - 13.0	8.9	4.1	3.4 - 8.3	3.3	2.2	0.085 - 0.17	TAM 112	chr2D_71764015	C/T	C	y		18BMS, 18BSP100, 19BSP100, across 6 envs
Qperi.tamu.4A.84	4A	83.8 - 90.5	PERI	3.4 - 6.2	3.7 - 10.1	6.8	3.3	1.5 - 7.7	2.4	1.4	-0.178 - -0.072	Duster	chr4A_30316423	G/C	G	y		18DMS, 19BMS, 19BSP100, across 6 envs
Qperi.tamu.5D.252	5D	252.0	PERI	3.4	9.5			8.9			-0.17	Duster	chr5D_233797558	C/T	C		y	18BMS
Qperi.tamu.7D.107	7D	107 - 109.8	PERI	3.4 - 3.4	4.8 - 5.2			2.1 - 7.2			0.16 - 0.17	TAM 112	chr7D_110098182	T/C	T	y		19BMS, 19BSP100, across 6 envs
Qperi.tamu.7D.64	7D	62.2 - 66	PERI	3.4 - 6.2	3.9 - 14.2	8	6.3	4.2 - 12.8	2.9	2.6	0.08 - 0.23	TAM 112	chr7D_53634310	A/G	A	y	y	18BI, 18BMS, across 6 envs
Qph.tamu.2D.37	2D	37.1 - 37.4	PH	3.4 - 7.0	3.8 - 25.5	12.2	13.3	3.6 - 11.3	3.3	4.8	0.7 - 1.9	TAM 112	chr2D_35558557	G/A	G	y	y	18BI, 18DMS, 19BMS, 19BSP100, across 8 envs
Qph.tamu.2D.620	2D	619.7 - 620.2	PH	3.4 - 7.0	3.6 - 12.4	9.4	3.1	3.5 - 7.6	2.3	1.2	0.6 - 1.7	TAM 112	chr2D_620685649	T/G	T	y		18BI, 19BMS, across 8 envs
Qph.tamu.5D.338	5D	338 - 350.8	PH	3.4 - 7	4.4 - 14.3	9.0	5.3	4.9 - 8.3	2.3	2.8	-2.23 - -0.62	Duster	chr5D_269148535	G/A	G	y		18BI, 18DMS, across 8 envs
Qph.tamu.6A.122	6A	121.8	PH	3.4 - 7.0	4.2 - 59.6	45.7	13.8	8.0 - 21.8	12.9	8.5	-3.2 - -1.1	Duster	chr6A_115036912	G/A	G	y		18BI, 18BMS, 18DMS, 19BMS, 19BSP100, across 8 envs
Qph.tamu.6A.418	6A	418.4	PH	3.4 - 7.0	4.4 - 30.1	8.4	21.7	8.0 - 13.6	2.2	5.9	-3.2 - -0.6	Duster	chr6A_406554946	T/G	T	y		17BSP100, 18BSP100, across 8 envs
Qph.tamu.6D.110	6D	110.4	PH	3.4 - 7.0	4.8 - 9.0	5.8	3.2	2.0 - 10.4	1.5	0.5	0.5 - 1.2	TAM 112	chr6D_110425575	T/C	T		y	18BSP100, across 8 envs
Qscw.tamu.2D.37	2D	37.4	SCW	3.4 - 7.6	5.5 - 10.9	6.6	4.3	2.7 - 15.2	2.3	0.4	0.026 - 0.045	TAM 112	chr2D_37467471	A/T	A		y	18BSP100, across 9 envs
Qscw.tamu.5D.274	5D	273.8 - 299.5	SCW	3.4 - 7.6	4.0 - 22.8	15.9	6.8	8.3 - 15	5.4	2.8	-0.10 - -0.04	Duster	chr5D_269148535	G/A	G	y		18BMS, 18DMS, across 9 envs
Qscw.tamu.7A.6	7A	5.7 - 7.0	SCW	3.4 - 7.6	3.6 - 10.8	7.7	3.2	4.9 - 9.5	2.5	2.4	-0.097 - -0.027	Duster	chr7A_2579229	G/A	G	y		19BMS, 19BSP100, across 9 envs
Qshgw.tamu.2B.58	2B	57.5 - 61.6	SHGW	3.4 - 7.5	5.9 - 10.8	6.2	4.6	5.1 - 11.2	3.1	2.0	10.0 - 30.0	TAM 112	chr2B_57246450	A/C	A		y	17BSP100, across 9 envs
Qshgw.tamu.2D.37	2D	37.1	SHGW	3.5 - 7.5	3.6 - 8.3	0.9	7.4	4.2 - 7.3	0.5	3.7	-22.8 - -3.9	Duster	chr2D_37224692	C/T	C		y	17BSP67, across 9 envs
Qsl.tamu.5D.252	5D	252.0	SL	3.4 - 4.2	5.9 - 7.4	1.7	5.7	4.9 - 8.8	2.2	2.7	-0.18 - -0.10	Duster	chr5D_233797558	C/T	C		y	17BSP100, across 2 envs
Qspm.tamu.2D.43	2D	42.9	SPM	3.4 - 7.6	6.3 - 9.5	1.2 - 5.8	1.4 - 8.3	2.7 - 15.2	0.6 - 7.7	1.4 - 2.1	-30.8 - -6.1	Duster	chr2D_42872548	T/C	T		y	17BSP100, ME3, across 9 envs
Qspm.tamu.2D.37	2D	37.1 - 37.4	SPM	3.4 - 4.6	4.9 - 5.2	1.7	3.5	5.0 - 12.9	1.9	3.1	-33.0 - -7.5	Duster	chr2D_37467471	A/T	A		y	18BSP100, ME123
Qspm.tamu.4D.64	4D	64.2	SPM	4.6 - 7.5	10.0 -10.4	5.2 - 7.8	2.6 - 4.7	5.0 - 8.5	3.5 - 6.1	1.5 - 2.5	-13.4 - -3.6	Duster	chr4D_61853616	A/C	A		y	ME123, across 9 envs
Qspm.tamu.5D.252	5D	252.0	SPM	7.5	8.1	6.1	2.0	3.7	2.7	1.0	13.3	TAM 112	chr5D_233797558	C/T	C		y	across 9 envs
Qtkw.tamu.2B.62	2B	61.6	TKW	3.4 - 7.6	7.7 - 22.5	8.9 - 12.6	2.2 - 12.7	4.7 - 18.0	4.0 - 11.3	2.4 - 6.7	0.3 - 1.0	TAM 112	chr2B_57246450	A/C	A	y	y	17BSP67, 17BSP100, ME1, ME12, across 9 envs
Qtkw.tamu.2B.137	2B	136.9	TKW	3.4	3.5 - 4.4			5.9 - 9.2			0.75 - 0.81	TAM 112	chr2B_138085124	C/T	C	y		17BMS, 18DMS
Qtkw.tamu.2B.785	2B	784.7	TKW	3.4 - 7.6	5.8 - 18.7	5.9 - 8.8	0.4 - 4.7	4.4 - 12.9	3.6 - 5.8	0.6 - 2.9	0.3 - 1.5	TAM 112	chr2B_781532381	A/C	A	y		17BSP67, 17BSP100, ME1, ME12, across 9 envs
Qtkw.tamu.2D.37	2D	37.4 - 38.7	TKW	3.4 - 7.6	3.7 - 37.1	6.9 - 29.8	0.02 - 19.8	6.1 - 23.4	5.1 - 22.6	0.7 - 11.8	-1.4 - -0.4	Duster	chr2D_35558557	G/A	G	y	y	17BSP100, 18BI, 18DMS, 18BMS, 19BMS, ME1, ME2, ME12, across 9 envs
Qtkw.tamu.2D.72	2D	71.9 - 72.7	TKW	3.4 - 7.6	3.7 - 9.9	4.4 - 8.4	0.6 - 1.7	3.9 - 5.2	3.1 - 3.4	0.8 - 1.3	0.3 - 0.7	TAM 112	chr2D_71764015	C/T	C	y		18BI, 19BSP100, ME2, ME12, across 9 envs
Qtkw.tamu.4A.84	4A	83.8 - 90.5	TKW	3.4 - 7.6	3.7 - 19	5.5 - 12.1	0.5 - 7	2.8 - 8.7	4.2 - 5.0	0.7 - 3.7	-0.8 - -0.36	Duster	chr4A_30316423	G/C	G	y		17BSP67, 17BSP100, 18DMS, 19BMS, 19BSP100, ME1, ME2, across 9 envs
Qtkw.tamu.5D.252	5D	252.0	TKW	4.8	8.0	7.8	0.1	5.8	5.6	0.2	-0.5	Duster	chr5D_233797558	C/T	C		y	ME2
Qtkw.tamu.6A.583	6A	583.2 - 584.4	TKW	5.3 - 7.6	8.3 - 10.0	2.1 - 2.9	6.2 - 7.1	4.0 - 7.1	1.2 - 1.9	2.8 - 5.2	-0.18 - -0.22	Duster	chr6A_574253510	G/A	G		y	ME1, across 8 envs
Qtkw.tamu.6B.139	6B	138.7	TKW	3.4 - 4.3	3.6 - 7.4	5.4	2.0	2.8 - 5.4	3.5	1.9	0.4 - 0.7	TAM 112	chr6B_135720799	C/G	C	y		17BSP67, 17BSP100, ME12
Qtkw.tamu.6D.116	6D	115.7	TKW	4.3	4.3	4.3	0.04	2.8	2.7	0.1	0.4	TAM 112	chr6D_97915036	G/A	G		y	ME12
Qtkw.tamu.7D.66	7D	66 - 69.2	TKW	3.4 - 7.6	9.3 - 26.2	8.3 - 18	2.5 - 8.9	6.9 - 16.8	5.3 - 13.7	1.6 - 4.3	0.43 - 1.2	TAM 112	chr7D_64236400	G/T	G	y	y	18BI, 18BMS, ME2, ME12, across 9 envs
Qtw.tamu.2B.44	2B	43.8	TW	6.1	6.7	5.6	1.1	5.3	3.6	1.7	2.8	TAM 112	chr2B_42242271	A/G	A		y	across 6 envs
Qtw.tamu.2D.37	2D	37.4 - 38.7	TW	3.4 - 6.1	4.5 - 15.2	10.2	5.1	10.3 - 25.8	7	12.7	-8.8 - -0.6	Duster	chr2D_35558557	G/A	G	y	y	17RDD, 18NFD, 19RRD, 19BSP100, across 6 envs
Qtw.tamu.6A.586	6A	585.5 - 586.7	TW	3.5 - 6.1	3.5 - 8.3	5.2	3.1	5.5 - 6.2	3.4	2.8	-2.7 - -6.5	Duster	chr6A_583269008	A/G	A		y	19RDD, across 6 envs
Qyld.tamu.2D.44	2D	43.7	YLD	4.7 - 8.0	6.5 - 8.3	1.1 - 3.0	5.3	7.8 - 10.0	1.8	6.0 - 8.2	-2.6 - -2.1	Duster	chr2D_42872584	A/C	A		y	ME123, across 9 envs
Qyld.tamu.2D.3	2D	2.5 - 3.3	YLD	3.3 - 8.0	5.8 - 18.3	4.2 - 8.8	2.3 - 13.0	6.1 - 11.0	3.3 - 6.9	0.5 - 2.9	-14.2 - -3.6	Duster	chr2D_682149	G/T	G	y		17CH, 17RRD, 19RRD, ME2, ME123, across 10 envs
Qyld.tamu.2D.37	2D	37.1 - 38.7	YLD	3.4 - 8.0	3.7 - 17.4	0.6 - 6.8	0.8 - 13.4	2.8 - 17.6	0.4 - 9.9	1.9 - 7.8	-11.7 - -1.2	Duster	chr2D_35558557	G/A	G	y	y	17CH, 17RRD, 19RDD, ME2, ME3, ME123, across 10 envs
Qyld.tamu.4D.64	4D	64.2	YLD	3.4 - 8.0	10.6 - 15.7	5.4 - 7.8	5.6 - 10.3	10.4 - 15.8	3.4 - 8.8	4.7 - 7.1	-14.2 - -3.6	Duster	chr4D_61853616	A/C	A		y	19BSP100, ME1, across 9 envs
Qyld.tamu.6A.113	6A	113.0	YLD	3.4 - 4.7	3.7 - 5.1	2.9	2.2	9.3 - 15.6	4.7	10.9	3.3 - 12.6	TAM 112	chr6A_122456115	C/T	C		y	18BI, ME123

a Physical position based on IWGSC RefSeq v 1.0 Mega base pair position.

b Abbreviation of traits: grain yield form whole plot (YLD), thousand kernel weight (TKW), kernels spike^-1^ (KPS), spikes m^-2^ (SPM), harvest index (HI), dry weight of biomass sample (BM), grain yield from biomass sample (BMYLD), single head dry weight (SHDW), single head grain weight (SHGW), single culm weight (SCW), heading date (HD), plant height (PH), test weight (TW), kernel area (AREA), kernel perimeter (PERI), kernel length (KLEN), kernel width (KWID), spike length (SL).

c Logarithm of the odds value for overall, additive effect (A), and additive by environment interaction effect (AbyE).

d Phenotypic variance explained for overall, additive effect (A), and additive by environment interaction effect (AbyE).

e Additive effect value from single allele for positive value with favorable allele from TAM 112 and negative value with favorable allele from Duster.

f QTL mapping under individual environment, across all environments, within mega-environment, and across mega-environments.Abbreviation of environments: Texas A&M AgriLife Research stations in Bushland, TX, Bushland middle school dryland in 2017, 2018, 2019 (17BMS, 18BMS, 19BMS), Bushland irrigated in 2018 (18BI), and Bushland South Pivot with two irrigated levels (67% and 100%) in 2017, 2018, 2019 (17BSP67, 17BSP100, 18BSP100, 19BSP100), Red River Demonstration Farm, CO, in 2017, 2019 (17RRD, 19RRD), Noble Foundation Dupy Farm, CO, in 2018 (18NFD), Dumas, TX, in 2018 (18DMS), Chillicothe, TX, in 2017 (2017CH). Mega-environments (MEs) are as follow: BMYLD: ME1 (17BSP67, 18BSP100), ME2 (17BMS, 17BSP100, 19BSP100), KLEN: ME1 (19BMS, 19BSP100), ME2 (18BI, 18BMS), KWID: ME1 (19BMS, 19BSP100), ME2 (18BI, 18BMS), HI: ME1 (18BI, 19BMS), ME2 (17BSP67, 17BSP100, 19BSP100), ME3 (17BMS, 18BSP100), KPS: ME1 (17BMS, 17BSP67, 17BSP100, 19BSP100), ME2 (18BI, 18BMS, 19BMS), SPM: ME1 (19BMS, 19BSP100), ME2 (17BMS, 18BMS, 18BI), ME3 (17BSP67, 17BSP100), TKW: ME1 (17BMS, 17BSP67, 17BSP100, 19BSP100), ME2 (18BI, 18BMS, 19BMS), YLD: ME1 (18BI, 19BMS, 19BSP100), ME2 (17CH, 17BMS, 19RRD), ME3 (17RRD, 18BSP100, 18NFD).

### Yield and yield component traits

A set of 10 consistent QTL associated with yield and yield components was mapped on chromosomes 2B, 2D, 2A, 6A, 6B, and 7D (**Table 1** and **Figure 1**). Among them, the QTL on 2D at 37.1 – 38.9 Mbps showed consistent effects on YLD, BMYLD, KPS, TKW, and HI. For YLD and BMYLD, *Qyld.tamu.2D.37* had LOD scores ranging 3.7 – 17.4, PVE values ranging 2.8 – 17.6%, and increased YLD up to 11.7 g m^-2^ in 17RRD. While *Qbmyld.tamu.2D.37* had LOD scores ranging 3.8 – 15.4, PVE ranging 9.0 – 26.5%, and increased BMYLD up to 28.3 g m^-2^ in 17BSP100. The favorable alleles that increased YLD and BMYLD were from Duster. For TKW and KPS, *Qtkw.tamu.2D.3*7 had LOD scores of 3.7 – 37.1, PVE values of 6.1 – 23.4%, and increased 1.4 g of TKW in 17BSP100 with the Duster allele. *Qkps.tamu.2D.37* had LOD scores of 4.3 – 20.8, PVE of 5.0 – 13.5%, and increased KPS up to 1.10 kernel spike^-1^ in 19BMS with the TAM 112 allele. In addition, a consistent YLD QTL was also identified on chromosome 2D at 2.5 – 3.3 Mbps with the favorable allele from Duster that increased YLD up to 14.2 g m^-2^ in 17RDD, with LOD scores of 5.8 – 18.3, and PVE of 6.1 – 11.0%. Another consistent KPS QTL was identified on 6A at 113 Mbps with the favorable allele from TAM 112 that had LOD scores ranging 3.8 – 17.3, PVE ranging 7.1 – 11.6, and increased KPS up to 1.4 kernel spike^-1^ in 17BMS. Moreover, another seven consistent QTL for TKW were identified on chromosomes 2B (61.6, 136.9, and 784.7 Mbps), 2D (72.7 Mbps), 4A (83.8 – 90.5 Mbps), 6B (138.7 Mbps), and 7D (66 – 69.2 Mbps) that explained 2.8 – 23.4% of TKW variations with LOD scores of 3.5 – 37.1. Except for *Qtkw.tamu.4A.84*, all these TKW QTL had favorable alleles from TAM 112 that increased TKW up to 1.5 g. In summary, all YLD and BMYLD QTL were increased by Duster alleles, while all KPS QTL and most of TKW QTL were increased by TAM 112 alleles (**Table 1**). Although there were 11 QTL regions associated with SPM, none of them were consistent QTL (**Table S6** and **Figure 1**).

### Kernel size traits

Eleven kernel size related consistent QTL were identified on chromosomes 1A, 2B, 2D, 3A, 4A, 5D, and 7D (**Table 1** and **Figure 1**). For AREA, five consistent QTL were located on chromosomes 2D (37.2 – 38.7 Mbps, 71.9 – 72.7 Mbps), 4A (83.8 – 90.5 Mbps), 5D (196.9 Mbps), and 7D (66 – 67.7 Mbps). They had LOD scores ranging 3.7 – 26.7, PVE ranging 2.0 – 12.9%, and increased AREA up to 0.34 mm^2^. The beneficial alleles of *Qarea.tamu.2D.72* and *Qarea.tamu.7D.66* were from TAM 112 while the rest three QTL had beneficial alleles from Duster. Six KLEN QTL were mapped on chromosomes 1A (324.6 Mbps), 2B (688.4 Mbps, 781.2 – 784.7 Mbps), 3A (664.0 – 667.5 Mbps), 4A (83.8 – 90.5 Mbps), and 7D (156.8 Mbps). The LOD scores and PVE values of these QTL ranged 3.6 – 56.5 and 1.9 – 33.6, respectively. The additive effect of *Qklen.tamu.3A.664* for KLEN was up to 0.2 mm in 19BMS, with the favorable alleles from Duster. Only two consistent KWID QTL were mapped on chromosomes 2D (37.1 – 37.4 Mbps) and 7D (62.2 – 66.0 Mbps) that increased KWID up to 0.037 mm^2^, with LOD scores of 3.5 – 17.2, and PVE values of 7.3 – 16.2%. The favorable alleles were from Duster and TAM 112, respectively. Moreover, six consistent QTL that increased PERI up to 0.23 mm were located on chromosomes 1A (324.6 Mbps), 2D (37.1 – 38.7 Mbps, 71.9 – 72.7 Mbps), 4A (83.8 – 90.5 Mbps), and 7D (62.2 – 66.0 Mbps, 107.0 – 109.8 Mbps) with LOD scores ranging 3.6 – 22.8 and PVE values ranging 1.5 – 15.7%. All of the PERI QTL had favorable alleles from TAM 112, except *Qperi.tamu.2D.37* and *Qperi.tamu.4A.84*. Among all of detected kernel size related QTL, TAM 112 and Duster each contributed half of the favorable alleles (**Table 1**).

In addition, colocalizations were observed for kernel size related consistent QTL. QTL on chromosome 1A at 324.6 Mbps was linked to KLEN and PERI. QTL on 2D at 37.1 – 38.7 Mbps and 7D at 62.2 – 67.7 Mbps were associated with AREA, KWID, PERI. QTL on 2D at 71.9 – 72.7 Mbps was linked to AREA and PERI while the QTL on 4A at 83.8 - 90.5 Mbps was associated with AREA, PERI, and KLEN (**Table 1** and **Figure 1**).

### Agronomy traits

In total, eight agronomic consistent QTL were detected on chromosomes 2B, 2D, 5D, 6A, 7A, and 7D (**Table 1** and **Figure 1**). One major QTL on 2D at 37.1 – 38.7 Mbps consistently influenced HD, PH, TW, and increased these traits up to 2.2 days, 2.2%, 1.9 cm, and 8.8 Kg m^-3^. For HD, *Qhd.tamu.2D.37* had LOD scores of 20.4 – 82.8 and PVE values of 22.0 – 39.9%. For PH, *Qph.tamu.2D.37* had LOD scores of 3.8 – 25.5 and PVE values of 3.6 – 11.3%. The LOD scores of Qhi.tamu.2D.37 and Qtw.tamu.2D.37 were ranged from 7.4 – 46.7, 4.5 – 15.2 while their PVE values were ranged from 13.4 – 40.7% and 10.3 – 25.8%, respectively. The increasing allele was from TAM 112 for HD and PH, and from Duster for TW. Furthermore, we detected four consistent PH QTL located on chromosomes 2D (619.7 – 620.2 Mbps), 5D (338 – 350.8 Mbps), 6A (121.8 Mbps, 418.4 Mbps) that increased PH up to 3.2 cm, explained 3.5 – 21.8% of phenotype variations, and with LOD scores of 3.6 – 59.6. All three QTL had favorable alleles from Duster except *Qph.tamu.2D.620*. Two HD QTL were consistently detected on chromosomes 2B (57.6 Mbps) and 2D (37.4 – 38.7 Mbps) that increased HD up to 1.7 days with favorable alleles from Duster. Their LOD score and PVE value ranged 4.4 – 64.5 and 2.9 – 25.3%, respectively. Moreover, we detected two consistent QTL for SCW on chromosomes 5D (273.8 – 299.5 Mbps) and 7A (5.7 – 7.0 Mbps) with favorable alleles from Duster. Two SCW QTL had LOD scores of 3.6 – 22.8 and PVE values of 4.9 – 15%, which increased SCW up to 0.1 g. Nearly all above mentioned QTL had increasing alleles from Duster, while only one QTL for HD and two QTL for PH had favorable alleles from TAM 112 (**Table 1**).

### Pleiotropic QTL

The QTL analysis identified 30 pleiotropic QTL regions associated with at least two traits without strong correlations and 13 QTL regions in common with consistent QTL regions (**Tables 1**, **S6**). Pleiotropic QTL linked to yield and yield-related traits were detected on chromosomes 2B, 2D, 4D, 5D, 6A, 6D, and 7D. Among them, four QTL regions with large effects were classified as major pleiotropic QTL. The first QTL region was located on chromosome 2B between 57.5 and 61.6 Mbps with TAM 112 alleles that increased HI, TKW, and KWID, while HD was delayed by the Duster allele. The second QTL region was located on chromosome 2D at 37.1 – 38.7 Mbps, where the Duster alleles increased AREA, BMYLD, HI, KLEN, KWID, PERI, SPM, TKW, TW, and YLD, while TAM 112 allele increased HD, KPS, and PH. The third QTL region on chromosome 6A at 113.0 Mbps, where the TAM 112 alleles increased HI, KPS, and YLD, while Duster alleles increased HD and KWID. The fourth QTL region on chromosome 7D at 62.2 – 69.2 Mbps increased AREA, HI, KLEN, KWID, PERI, and TKW with TAM 112 alleles, while Duster alleles increased HD and KPS.

Moreover, six pleiotropic QTL regions with minor effects were also identified (**Figure 1** and **Tables 1**, **S6**). The pleiotropic QTL on chromosome 2B at 41.8 – 43.8 Mbps was related to HD, HI, and TW. On chromosome 2D at 42.9 – 43.7 Mbps, a QTL region was associated with BM, SPM, BMYLD, and YLD. Another QTL controlling SPM and YLD was identified on chromosome 4D at 64.2 Mbps. The QTL region located on chromosome 5D at 252.0 Mbps increased AREA, SL, KLEN, KWID, PERI, and TKW with Duster alleles but TAM 112 alleles increased SPM. Another QTL region on chromosome 6A at 583.2 – 586.7 Mbps increased TKW and TW with alleles from Duster. Furthermore, the QTL region on 6D at 110.4 – 115.7 Mbps increased KPS with the Duster allele while TAM 112 alleles increased TKW, AREA, and PH (**Figure 1** and **Tables 1**, **S6**).

### Epistatic interactions

A total of 185 epistatic interactions (EI) with a LOD score ≥ 10 were identified for fifteen yield, yield-related, kernel and agronomic traits from individual environments, within, and across mega-environments (**Figure S6** and **Table S7**). Among all EI, two interactions for AREA and one for each SPM, TW, and SCW explained 2.2 – 2.5% of the phenotypic variation. For YLD, a total of 16 interactions were identified, including two interactions involving the consistent QTL *Qyld.tamu.2D.3* that interacted with a locus on 2D at 605.0 Mbps. Its epistasis by environment interactions (AA x E) effects increased YLD by 9.0 g m^-2^ in 18BSP100. Meanwhile, this QTL also interacted with another locus on 7D at 598.7 Mbps and the AA x E effects increased YLD by 10.6 g m^-2^ in 18DMS. In addition, the interaction between SNPs on 1B at 171.7 Mbps and 7B at 60.5 Mbps had an epistatic effect which increased YLD by 4.6 g m^-2^. AA x E effects increased yield by 4.8 g m^-2^ in 19RRD with the TAM 112 allele, while it increased yield by 8.4 g m^-2^ in 18BI with the Duster allele. Although four EI were detected for the KPS, none of them were involved with mapped major QTL. Within 68 identified EI for TKW, six interactions were involved with five detected QTL (*Qtkw.tamu.2B.62*, *Qtkw.tamu.4A.84*, *Qtkw.tamu.4A.160, Qtkw.tamu.6B.640*, and *Qtkw.tamu.7D.69*). These interactions only explained 1.3 – 1.6% of the phenotypic variation and increased TKW up to 0.33 g. Among them, the maximum effects increased TKW up to 0.52 g with the Duster allele and up to 0.8 g with the TAM 112 allele. A total of 12 EI were identified associated with KLEN, including one interaction from the consistent QTL *Qklen.tamu.4A.84* and a QTL on 7A at 181.3 Mbps that increased KLEN by 0.033 mm. Moreover, only one KWID interaction was detected, with the QTL on 6B at 28.5 Mbps and 7B at 727.8 Mbps, but no major QTL was involved (**Figure S6** and **Table S7**).

For HD, nine EI were detected involving three previously detected QTL, including *Qhd.tamu.2B.42*, *Qhd.tamu.2B.58*, and *Qhd.tamu.2D.151*, while only *Qhd.tamu.2B.58* was a consistent QTL. These three EI only explained 1.7 – 2.0% of the phenotypic variation with maximum effects less than 0.65 days. Among 24 HI EI, one involved with previously detected QTL *Qhi.tamu.3D.590*. This interaction increased HI by 0.43% in 17BSP100. The maximum effect only increased HI by 0.8%. Moreover, 28 interactions were detected for PH, including one interaction between major QTL *Qph.tamu.5D.398* and a marker on 5B at 601.4 Mbps. The maximum effect increased PH by 1.06 cm with the TAM 112 allele. Overall, not many major QTL were involved in the EI and the observed interaction effects were not significantly high (**Figure S6** and **Table S7**).

### Haplotype analysis

To assess the performance of different haplotypes, RILs were grouped according to their all-allelic combinations. Marker haplotypes identified 46 RILs carrying two QTL (*Qyld.tamu.2D.3*, *Qyld.tamu.2D.37*) for YLD in both 17RRD and 19RRD (**Figures S7-A1, A2**). Both QTL on chromosome 2D had insignificant effects on YLD when present alone in 17RDD. However, the QTL combination significantly increased YLD in both environments when compared with no QTL presence. For KPS, 46 RILs were also identified with two QTL (*Qkps.tamu.2D.37*, *Qkps.tamu.6A.113*) in both 17BSP67 and 18BI environments (**Figures S7-B1, B2**). The haplotype with only 6A QTL did not show significant effects by itself but it significantly increased KPS in combination with 2D QTL when compared to lines without QTL. There were four TKW QTL (*Qtkw.tamu.2B.62*, *Qtkw.tamu.2B.785*, *Qtkw.tamu.2D.37*, *Qtkw.tamu.4A.84*) mapped in 17BSP100 (**Figure S7-C1**) and two TKW QTL (*Qtkw.tamu.2D.37*, *Qtkw.tamu.7D.68*) mapped in 18BI (**Figure S7-C2**). In 17BSP100, at least two QTL in different combinations had significantly higher TKW than those without QTL, except when *Qtkw.tamu.2B.62* combined with *Qtkw.tamu.2B.785*, and *Qtkw.tamu.2B.785* combined with *Qtkw.tamu.4A.84*.

In 18BI, the mean TKW of those lines with both QTL on 2D and 7D was significantly higher than those lines with only 2D or without QTL. For KWID, 30 RILs had two QTL (*Qkwid.tamu.2D.37* and *Qkwid.tamu.7D.68*) in 18BMS (**Figure S7-D1**). Haplotypes with only one QTL or both QTL did not show significant differences on KWID. However, they were significantly higher than the haplotype without QTL. Only 12 RILs carried all four favorable alleles (*Qklen.tamu.1A.325*, *Qklen.tamu.2B.781*, *Qklen.tamu.3A.664*, and *Qklen.tamu.7D.157*) for KLEN in all three environments, 18BSP100, 19BSP100, and 19BMS (**Figure S7-E1, E2, E3**). Combining any three QTL significantly increased KLEN across all environments, except the combination of 2B, 3A, and 7D in 19BSP100.

## Discussion

### Phenotype under multiple environments

Phenotype evaluation under multiple environments is crucial for identifying consistent genomic regions linked to yield-related traits (Ogbonnaya et al., 2017). In the current study, both irrigated and dryland management was included in the field experiments, coupled with broad geographic regions including Texas High Plains, Texas Rolling Plains, and Oklahoma South Central Plains, which laid the foundation for exploring the stability of detected QTL regions (**Tables S1, S6**). Environmental variance showed significance and contributed a large proportion of the total phenotypic variation for all evaluated traits, which indicated the high environmental diversity among trials. Except for BM, the genetic variance for all traits was also significant, which indicated genetic stability (**Table S2**). A moderate to high entry-mean based broad-sense heritability (0.51 – 0.95) showed that most traits were selectable, except BM (0.10). The low heritability of BM was consistent with a previous study, which indicated BM was a low selectable trait (Yang et al., 2020). Since yield is a complex trait usually controlled by many minor effect genes and significantly influenced by environments (Li et al., 2007), the heritability of YLD (0.74) was comparatively high. The dissection of yield into yield components generally improves selection efficiency. Specific to this study, QTL for TKW, KPS, and SPM showed a higher additive effect proportion of the PVE compared to YLD and BMYLD (**Figures S5A, F–H** and **Table S6**). Correspondingly, the heritability of all three yield components was higher than the heritability of yield. The heritability estimates were highest for TKW followed by KPS and SPM, which agreed with a previous study (Guan et al., 2018). The further dissection of TKW into kernel traits had increased the heritability of KLEN (**Table S2**). The QTL for KWID and KLEN had higher additive PVE proportion than TKW (**Figures S5H, N, O** and **Table S6**).

The G x E is an essential source of phenotypic variation, providing important adaptability for plants in changing environments (Wang et al., 2014). Except for SPM, the observed G x E variances were highly significant for all traits due to the diverse phenotyping field environments (**Table S2**), indicating more environmentally stable genomic regions may control SPM. Environment-specific QTL and related G x E effects were detected with the MET analysis (**Table S6**). For instance, the YLD QTL on 2D at 2.5 – 3.3 Mbps was only mapped in three dryland environments (17RRD, 17CH and 19RRD), which is an indication of a specific G x E interaction. Those environment-specific QTL will be valuable to breeders for environment targeted gene deployment.

### Pyramiding favorable alleles for yield-related traits

Wheat yield can be partitioned into three main components including SPM, KPS, and TKW (Cao et al., 2020). Understanding the relationship among yield components is essential for better gene deployment or for pyramiding favorable alleles associated with different yield components. Generally, overall yield improvement requires increasing either kernel number per unit area (KN) or TKW (Sukumaran et al., 2018; Golan et al., 2019). However, KN and TKW are usually negatively correlated (Kuchel et al., 2007; Guan et al., 2018). The compensation effects between these two factors were also observed. For example, the pleiotropic QTL region on 2D at 37.1 – 38.7 Mbps had Duster alleles that increased TKW, SPM and YLD, while the TAM 112 allele increased KPS. Reversely, the pleiotropic QTL region on 7D at 62.2 – 69.2 Mbps had TAM 112 alleles increasing TKW, while Duster alleles increased KPS. The introgression of a different combination of these alleles may improve KN and/or TKW and relieve the unfavorable allele effect, which needs to be further verified. In addition, some QTL regions improved both yield and yield components without contrary favorable alleles from both parents, including the QTL region on chromosome 6A at 113 Mbps increased both YLD and KPS with the TAM 112 allele, and the QTL region on 2D at 42.9 – 43.7 Mbps increased both YLD and SPM with the Duster allele. Moreover, the improvement of YLD also can be achieved by increasing HI, such as the QTL region on 4D at 47.6 Mbps increased YLD and HI together with the Duster allele. These results indicated the potential to enhance YLD through synergistically improving TKW, KPS, SPM, and HI. In addition, we observed TKW colocalized with KWID on 1D at 135.3 – 137.8 Mbps, 2B at 61.6 and 184.3 Mbps, 3B at 813.4 – 814.4 Mbps, and 6B at 549.7 and 615.5 Mbps. TKW colocalized with KLEN on 2B at 781.2 – 784.7 Mbps, 2D at 71.9 – 72.7 Mbps, and 5D at 196.9 Mbps. Both KLEN and KWID colocalized with TKW on 1A at 590.8 – 592.1 Mbps, 2D at 37.1 – 38.7 Mbps, 5D at 252.0 Mbps, and 7D at 64.4 – 69.2 Mbps. These results indicated the potentially diverse mechanism for TKW determination.

The SNP haplotype analysis of yield and yield component traits estimated the potential effect of different allelic combinations at two, three and/or more loci. The SNP haplotype results suggested that more loci combinations had greater effects on traits including YLD, KPS, TKW, KLEN, and KWID and that gene pyramiding significantly improved performance as compared to the RILs without QTL. However, these QTL combinations need to be validated in other genetic backgrounds and diverse environments.

### Agronomic traits related to adaptability

Heading date determined the adaption of wheat to broad geographic locations and diverse climatic environments, which was also a critical agronomic trait in dry climate (Zanke et al., 2014). In the U.S Southern High Plains, drought is major stress limiting wheat yield (Xue et al., 2014). The early heading was favorable for reducing the time exposed to drought stress during the sensitive flowering and post-anthesis grain filling periods, hence increasing the TKW, TW, and HI in dryland environments (Shavrukov et al., 2017). Similarly, we identified three QTL regions consistently associated with HD. All of them had alleles from the early heading parent that increased at least one of YLD, TKW, HI, and TW.

Plant height is another important agronomic trait correlated with yield. The green revolution introduced the dwarf allele of *Rht1* into wheat cultivars that significantly reduced PH and improved both the HI and YLD (Allan, 1989; Hedden, 2003). However, under drought and heat stress conditions, the taller wheat usually had higher grain yield due to more biomass, longer coleoptile, and higher growth vigor that were favorable for a better yield performance (Jatayev et al., 2020). In our study, PH was negatively correlated with yield and yield components under both dry and irrigated environments except in 18BSP100 and 17BSP100. Except for the QTL on 2D at 37.4 Mbps, most of the identified PH QTL were physically located far away from the QTL linked to yield and yield-related traits. The only exception, *Qph.tamu.2D.37* colocalized with yield-related traits, and may be explained by the pleiotropic effect of *Ppd-D1*. *Qph.tamu.2D.37* was physically close to *Ppd-D1*, which was reported for its strong pleiotropic effect on all yield-related traits and PH (Guo et al., 2010). The diagnostic marker analysis also proved that Duster carried the insensitive *Ppd-D1a* allele, and had early HD (Beales et al., 2007). Moreover, the identified PH loci other than *Qph.tamu.2D.37* may not affect yield, which is consistent with the recently cloned *Rht8* gene, which does not influence coleoptile elongation and yield components like KPS and TKW (Chai et al., 2022).

### QTL comparisons

Yield-related traits are important in wheat breeding (Hu et al., 2020). Since 1900, yield-related traits were intensively selected and improved for the higher KPS, TKW, and HI (Calderini et al., 1995). Previous studies characterized numerous QTL and genes associated with yield-related traits across multiple environments on all 21 wheat chromosomes (Assanga et al., 2017; Cao et al., 2020; Yang et al., 2020; Dhakal et al., 2021a). To evaluate the reliability and novelty of those QTL, we compared the physical positions of QTL identified in this study with previously reported QTL and genes based on the IWGSC reference genome version 1.0.

For yield-related traits, the genome-wide association study (GWAS) of CIMMYT spring bread wheat conducted by Sehgal et al. (2020) identified a yield QTL that shared the same physical location with *Qbmyld.tamu.2B.75*. On chromosome 2B, between 57.5 and 61.6 Mbps, we mapped a major pleiotropic QTL associated with TKW, KWID, HD, HI, and SHGW. This genomic region is at the proximal side of the photoperiod gene *Ppd-B1*, which is known for its influence on YLD, HD, and TKW (Mohler et al., 2004; Maphosa et al., 2014). The previously reported gene *TaDA1* located on chromosome 2D at 8.3 Mbps, had effects on TKW, KLEN, and KWID (Liu et al., 2020a). *Qyld.tamu.2D.3* was an environment-specific QTL only detected under dryland environments in Oklahoma and Chillicothe in Texas. Although the physical position of *Qyld.tamu.2D.3* is in the close distal side of *TaDA1*, the yield components and kernel traits were not evaluated under the abovementioned environments. Hence those traits still need to be explored in future studies to see if *Qyld.tamu.2D.3* also affects TKW and kernel traits. The *Qbmyld.tamu.2D.37* in the proximal side of photoperiod gene *Ppd-D1* at 33.9 Mbps. The diagnostic marker analysis also detected Duster has an insensitive *Ppd-D1a* allele. Previous studies revealed that different haplotypes of *Ppd-D1* under multiple environments influenced YLD, KPS, TKW, HI, HD, and PH (Guo et al., 2010; Chen et al., 2018), which were consistent with the result from this study. *Qyld.tamu.6A.113* was colocalized with *Qkwid.tamu.6A.113* and *Qkps.tamu.6A.113*. Several studies reported loci linked to YLD, KWID, and KPS located at similar physical positions, proving the reliability of this QTL region as a consistent and pleiotropic QTL (Assanga et al., 2017; Li et al., 2019a; Li et al., 2019b; Duan et al., 2020; Liu et al., 2020c; Dhakal et al., 2021b). In the Nongda3338/Jingdong6 doubled haploid (DH) population, Guan et al. (2018) identified a stable SPM QTL *QEp.cau-4D.1* which overlapped with the *Qspm.tamu.4D.64* identified in this study. The peak marker of *Qspm.tamu.5D.252* was only 2 Mbps proximal to the peak marker of the SPM QTL detected by Li et al. (2020). Moreover, the physical position of *Qspm.tamu.7A.4* overlapped with the locus linked to SPM in a population of 246 F_8_ RILs derived from the cross of Zhou 8425B/Chinese Spring (Gao et al., 2015). In addition, we detected a stable QTL region related to KPS, HI, TKW, KLEN, KWID, AREA, PERI, and HD, which was located on 7D between 64.4 and 69.2 Mbps. This genomic region contains *FT-D1*, a homolog gene of Arabidopsis *FLOWERING LOCUS T* that regulates wheat flowering and has been frequently reported by many previous studies (Sun et al., 2017; Li et al., 2019; Ward et al., 2019; Chen et al., 2020; Hu et al., 2020; Liu et al., 2020b; Liu et al., 2020c; Yang et al., 2020; Dhakal et al., 2021a; Isham et al., 2021). Isham et al. (2021) reported that *FT-D1* had an effect on TKW and KPS, and a recent study by Chen et al. (2022) further proved its effects on HD and KPS through gene editing. Those results were consistent with the traits associated with the *FT-D1* locus detected in this study. The diagnostic marker Vrn-D3-KASP was used to test both parents, which showed TAM 112 carried early heading and Duster carried late heading allele. Moreover, the QTL region on 7D between 107 and 109.8 Mbps linked to KLEN and PERI co-localized with the KLEN QTL detected in three wheat RIL populations (Li et al., 2018).

For TKW and kernel traits, *Qklen.tamu.1A.517* and *Qkwid.tamu.1A.591* had the physical position identical to KLEN and KWID QTL mapped in 163 bread wheat lines (Sun et al., 2017). The TKW QTL located on 2B at 136.9 Mbps, TKW and KWID QTL located on 2B at 184.3 Mbps, AREA and KLEN QTL located on 2B at 647.6 Mbps, and TKW and KLEN QTL located on 2B at 781.2 – 784.7 Mbps shared similar physical locations with previously reported QTL (Assanga et al., 2017; Sun et al., 2017; Zhang et al., 2018; Ma et al., 2019; Rahimi et al., 2019; Corsi et al., 2021). Besides, on chromosome 2D between 71.9 and 72.7 Mbps, a QTL region controlling TKW, KLEN, PERI, and AREA was close to previously reported QTL associated with TKW, KLEN, and AREA (Ma et al., 2018; Ma et al., 2019; Liu et al., 2020c; Qu et al., 2021). The genomic region on chromosome 4A between 83.8 and 90.5 Mbps harbored a QTL cluster associated with TKW, KLEN, PERI, and AREA, which has also been reported for association with TKW and KLEN (Sun et al., 2017; Ren et al., 2021). QTL associated with KWID was identified on chromosome 4A at 122.3 Mbps and was physically close to a KWID-related QTL at 120.6 Mbps reported by Sun et al. (2017).

For PH, QTL on 5D at 338.0 Mbps and on 6A at 122 Mbps were also previously mapped (Li et al., 2019b). Another consistent QTL linked to PH on chromosome 6A at 418.4 Mbps was colocalized with the previously identified dwarf gene *Rht18* and *Rht24b*. Both genes affect the expression level of gibberellin (GA) 2-oxidase, TaGA2ox-A9 (Ford et al., 2018; Tian et al., 2021). We found that PH QTL also influenced KPS, HI, AREA, and KLEN. In addition, a locus linked to HD shared the exact physical location with *Qhd.tamu.2B.42* in TAM 112/TAM 111 population (Dhakal et al., 2021a).

After further comparing with the previously identified stable QTL and genes recently summarized by Cao et al. (2020), several novel genomic regions with stable effects were determined in this study. A major QTL region linked to KLEN on 1A at 324.6 Mbps was detected under all six evaluated individual environments, and across or within mega environments. The favorable allele was from TAM 112, consistent with TAM 112 having a higher KLEN than Duster. A major gene may underlie this QTL region that accounts for the consistent kernel shape difference between parents. Another novel QTL region was on 5D at 196.9 Mbps and had pleiotropic effects on TKW, KLEN, PERI, AREA, and SHDW, with favorable alleles from Duster. Moreover, the QTL associated with TKW on 6B at 138.7 Mbps is also a novel locus, with favorable alleles from TAM 112.

## Conclusion

In this study, a set of 18 yield-related, and agronomic traits were evaluated in the TAM 112/Duster RIL population which was tested in 13 environments for three crop years. Detected QTL included 21 consistent and 30 pleiotropic QTL regions with 10 in common. Three genes, on chromosomes 2B (*Ppd-B1*), 2D (*Ppd-D1*), and 7D (*FT-D1*), aligned with three major consistent and pleiotropic QTL regions, increasing yield and yield-related traits.

Six consistent QTL for YLD, TKW, KLEN, KPS, and PH have additive effects that constantly explained more than 5% of the phenotypic variations, including *Qyld.tamu.2D.3*, *Qtkw.tamu.2B.137*, *Qklen.tamu.1A.325*, *Qkps.tamu.6A.113*, *Qph.tamu.6A.122*, and *Qph.tamu.6A.418*. One novel KLEN QTL on 1A at 324.6 Mbps appeared in all testing environments, which might be a valuable locus for exploring the genetic mechanism of KLEN. These QTL could be potentially used for future breeding applications. Most identified pleiotropic QTL regions had significant compensation effects on yield components. The majority of consistent QTL showed a higher portion of additive effects which is selectable. In addition, no significant epistatic interactions were observed on major consistent and pleotropic QTL. These results will be useful on understanding the genetic basis of yield and agronomic traits, pyramiding the favorable alleles through MAS, and moving forward with any future fine-mapping studies. The RILs carrying most favorable alleles can be directly used in as germplasms in the breeding programs.

## Data availability statement

The original contributions presented in the study are publicly available. This data can be found here: Texas Data Repository, https://dataverse.tdl.org/dataset.xhtml?persistentId=doi:10.18738/T8/O8WIU3.

## Author contributions

ZW, SL, YR prepared manuscript and analyzed data. ZW, SD, MC collected phenotype data. SL, YR, SY, GB revised manuscript. SL, JR, AI, QX, DH applied funding, gave suggestions on data analysis, and helped with field management. AB, PS, GB, SW conducted GBS library preparation, DNA sequencing, SNP calling, and genotype data filtering. FM, WH, JA, XM, JB, SB managed field trials. All authors contributed to the article and approved the submitted version.
