# Supplementary material for: QTL mapping of yield components and kernel traits in wheat cultivars TAM 112 and Duster
Source: Front Plant Sci. 2022 Dec 7;13:1057701. doi: 10.3389/fpls.2022.1057701 (PMC9768232; doi:10.3389/fpls.2022.1057701)
Supplement: Supplementary file 1 [file DataSheet_1.pdf]

Supplement figure S1: Boxplot of yield, kernel, and agronomy traits. Environments: Texas A&M AgriLife Research stations in Bushland, TX, irrigated land in 2018 (18BI), Bushland middle school dryland in 2017, 2018, 2019 (17BMS, 18BMS, 19BMS). Bushland south pivot irrigated land in 2017 with 67% and 100% irrigation levels (17BSP67, 17BSP100), in 2018, 2019 with 100% irrigation level (18BSP100, 19BSP100). Chillicothe dryland in 2017 (17CH). Dumas irrigated in 2018 (18DMS). Noble Foundation Dupy Farm in 2018 (18NFD), Red River Research and Demonstration Farm in 2017, 2019 (17RRD, 19RRD). Traits: A) grain yield form whole plot (YLD, g m^{-2}); B) dry weight of biomass sample (BM, g m^{-2}); C) grain yield from biomass sample (BMYLD, g m^{-2}); D) test weight (TW, Kg m^{-3}); E) harvest index (HI, %); F) kernel spike^{-1} (KPS, kernel spike^{-1}); G) spike m^{-2} (SPM, spike m^{-2}); H) thousand kernel weight (TKW, g); I) single head dry weight (SHDW, mg); J) single head grain weight (SHGW, mg); K) single culm weight (SCW, g); L) kernel area (AREA, mm^2); M) kernel perimeter (PERI, mm); N) kernel length (KLEN, mm); O) kernel width (KWID, mm); P) spike length (SL, cm); Q) heading date (HD, days); R) plant height (PH, cm).

(A)

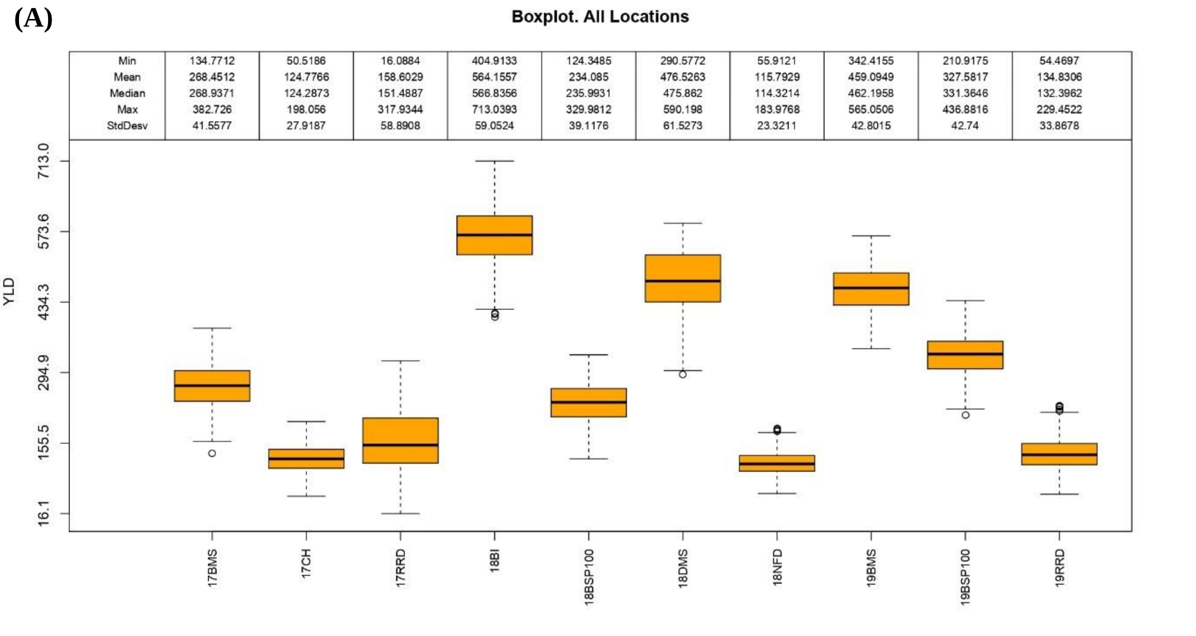

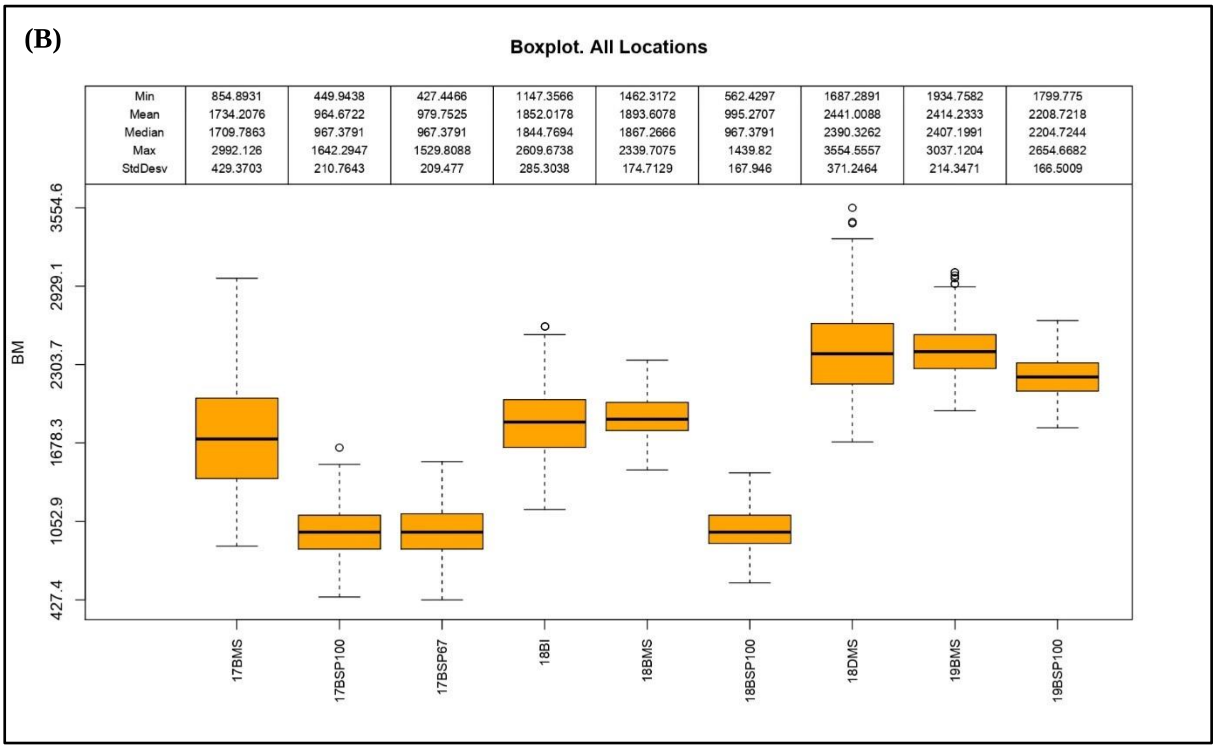
(B)
Boxplot. All Locations
Min 854.8931 449.9438 427.4466 1147.3566 1462.3172 562.4297 1687.2891 1934.7582 1799.775
Mean 1734.2076 964.6722 979.7525 1852.0178 1893.6078 995.2707 2441.0088 2414.2333 2208.7218
Median 1709.7863 967.3791 967.3791 1844.7694 1867.2666 967.3791 2390.3262 2407.1991 2204.7244
Max 2992.126 1642.2947 1529.8088 2609.6738 2339.7075 1439.82 3554.5557 3037.1204 2654.6682
StdDesv 429.3703 210.7643 209.477 285.3038 174.7129 167.946 371.2464 214.3471 166.5009
BM
3554.6
2929.1
2303.7
1678.3
1052.9
427.4
17BMS
17BSP100
17BSP67
18BI
18BMS
18BSP100
18DMS
19BMS
19BSP100

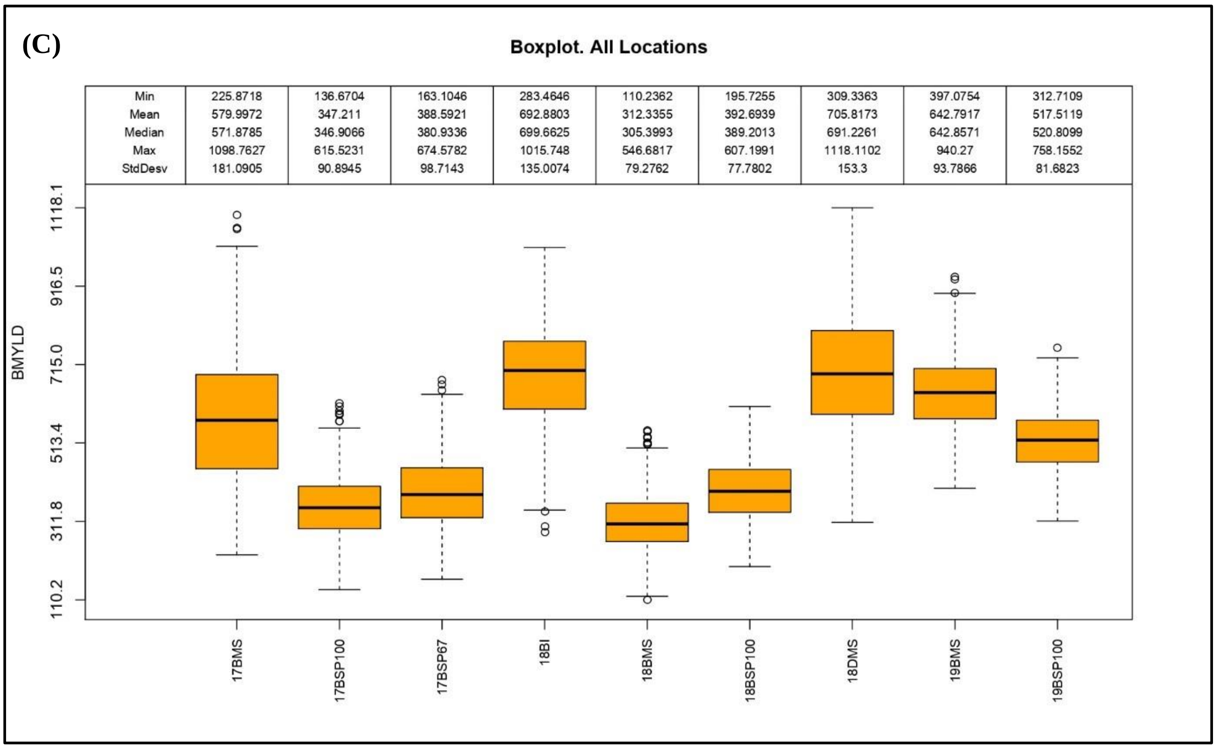
(C)
Boxplot. All Locations
Min 225.8718 136.6704 163.1046 283.4646 110.2362 195.7255 309.3363 397.0754 312.7109
Mean 579.9972 347.211 388.5921 692.8803 312.3355 392.6939 705.8173 642.7917 517.5119
Median 571.8785 346.9066 380.9336 699.6625 305.3993 389.2013 691.2261 642.8571 520.8099
Max 1098.7627 615.5231 674.5782 1015.748 546.6817 607.1991 1118.1102 940.27 758.1552
StdDesv 181.0905 90.8945 98.7143 135.0074 79.2762 77.7802 153.3 93.7866 81.6823
BMYLD
1118.1
916.5
715.0
513.4
311.8
110.2
17BMS
17BSP100
17BSP67
18BI
18BMS
18BSP100
18DMS
19BMS
19BSP100

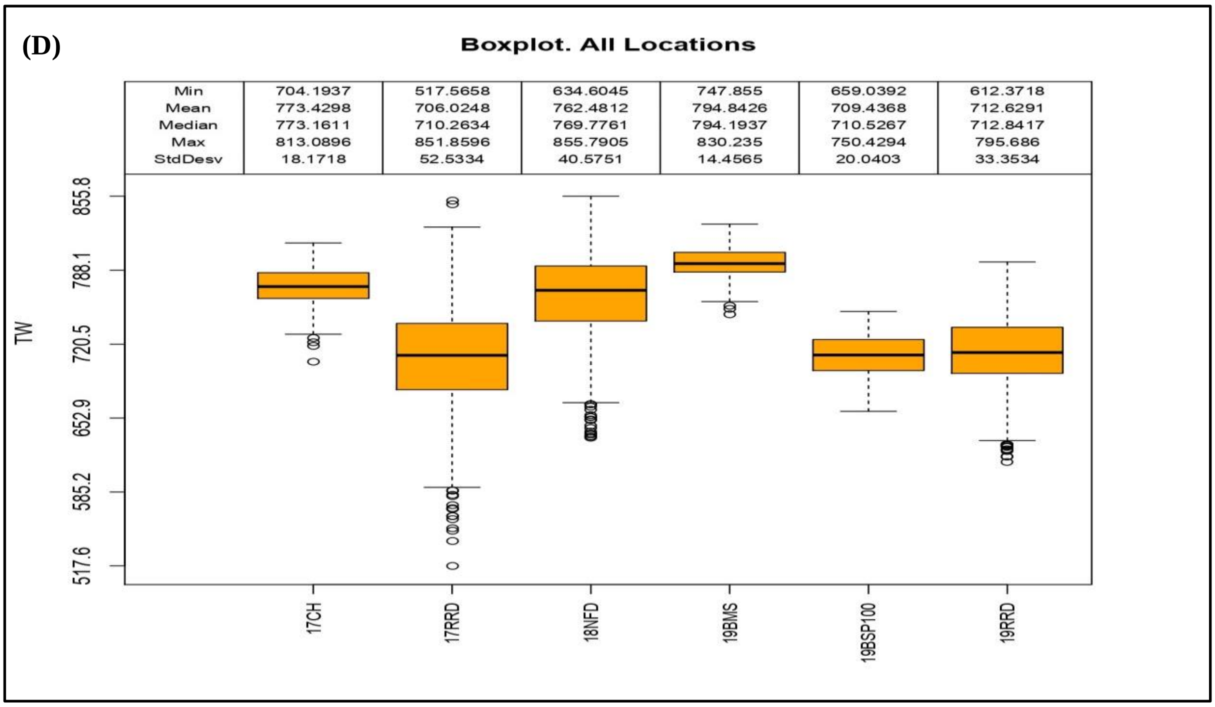
(D)
Boxplot. All Locations
Min 704.1937 517.5658 634.6045 747.855 659.0392 612.3718
Mean 773.4298 706.0248 762.4812 794.8426 709.4368 712.6291
Median 773.1611 710.2634 769.7761 794.1937 710.5267 712.8417
Max 813.0896 851.8596 855.7905 830.235 750.4294 795.686
StdDesv 18.1718 52.5334 40.5751 14.4565 20.0403 33.3534
TW
855.8
788.1
720.5
652.9
585.2
517.6
17CH
17RRD
18NFD
19BMS
19BSP100
19RRD

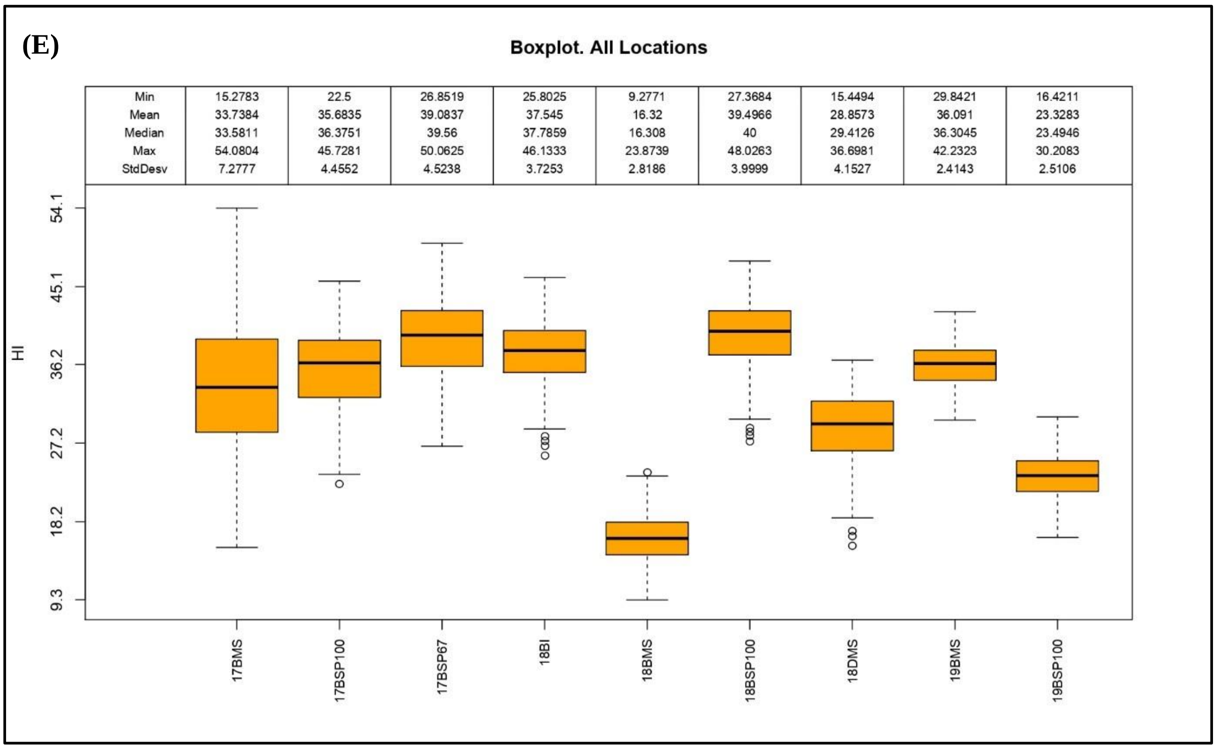
(E)
Boxplot. All Locations
Min 15.2783 22.5 26.8519 25.8025 9.2771 27.3684 15.4494 29.8421 16.4211
Mean 33.7384 35.6835 39.0837 37.545 16.32 39.4966 28.8573 36.091 23.3283
Median 33.5811 36.3751 39.56 37.7859 16.308 40 29.4126 36.3045 23.4946
Max 54.0804 45.7281 50.0625 46.1333 23.8739 48.0263 36.6981 42.2323 30.2083
StdDesv 7.2777 4.4552 4.5238 3.7253 2.8186 3.9999 4.1527 2.4143 2.5106
HI
54.1
45.1
36.2
27.2
18.2
9.3
17BMS
17BSP100
17BSP67
18BI
18BMS
18BSP100
18DMS
19BMS
19BSP100

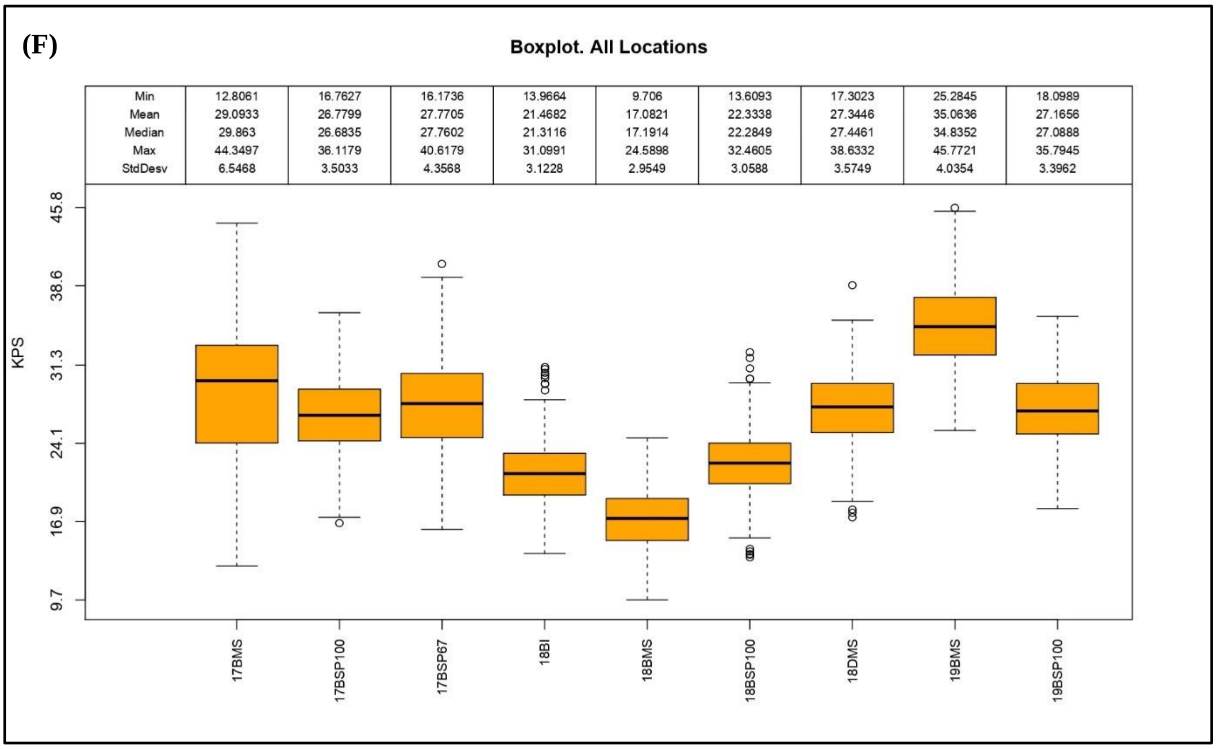
(F)
Boxplot. All Locations
Min 12.8061 16.7627 16.1736 13.9664 9.706 13.6093 17.3023 25.2845 18.0989
Mean 29.0933 26.7799 27.7705 21.4682 17.0821 22.3338 27.3446 35.0636 27.1656
Median 29.863 26.6835 27.7602 21.3116 17.1914 22.2849 27.4461 34.8352 27.0888
Max 44.3497 36.1179 40.6179 31.0991 24.5898 32.4605 38.6332 45.7721 35.7945
StdDesv 6.5468 3.5033 4.3568 3.1228 2.9549 3.0588 3.5749 4.0354 3.3962
KPS
45.8
38.6
31.3
24.1
16.9
9.7
17BMS
17BSP100
17BSP67
18BI
18BMS
18BSP100
18DMS
19BMS
19BSP100

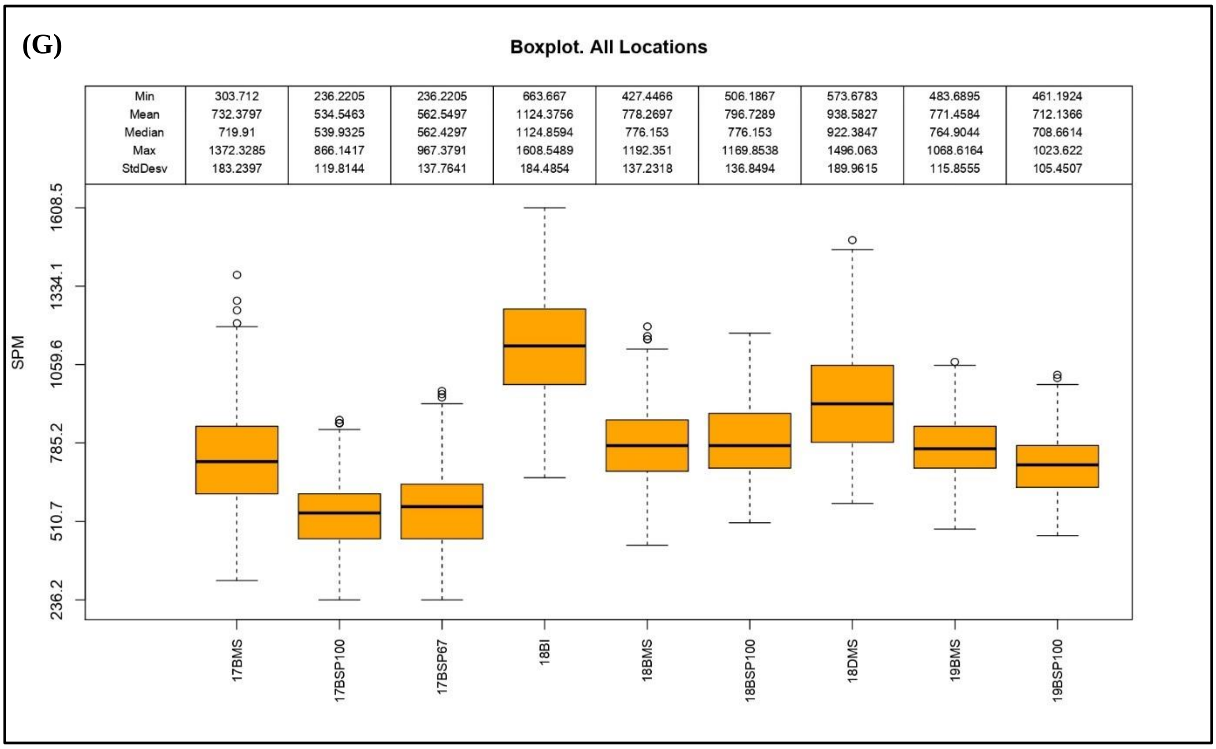
(G)
Boxplot. All Locations
Min 303.712 236.2205 236.2205 663.667 427.4466 506.1867 573.6783 483.6895 461.1924
Mean 732.3797 534.5463 562.5497 1124.3756 778.2697 796.7289 938.5827 771.4584 712.1366
Median 719.91 539.9325 562.4297 1124.8594 776.153 776.153 922.3847 764.9044 708.6614
Max 1372.3285 866.1417 967.3791 1608.5489 1192.351 1169.8538 1496.063 1068.6164 1023.622
StdDesv 183.2397 119.8144 137.7641 184.4854 137.2318 136.8494 189.9615 115.8555 105.4507
SPM
1608.5
1334.1
1059.6
785.2
510.7
236.2
17BMS
17BSP100
17BSP67
18BI
18BMS
18BSP100
18DMS
19BMS
19BSP100

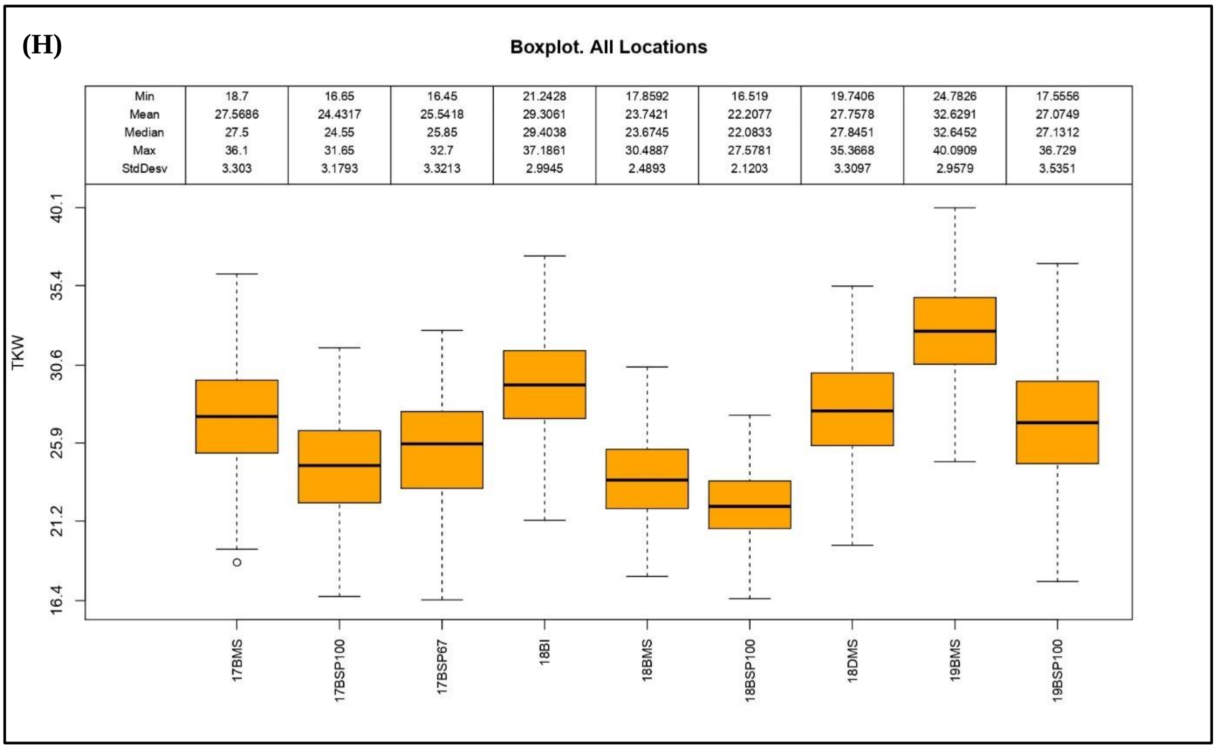
(H)
Boxplot. All Locations
Min 18.7 16.65 16.45 21.2428 17.8592 16.519 19.7406 24.7826 17.5556
Mean 27.5686 24.4317 25.5418 29.3061 23.7421 22.2077 27.7578 32.6291 27.0749
Median 27.5 24.55 25.85 29.4038 23.6745 22.0833 27.8451 32.6452 27.1312
Max 36.1 31.65 32.7 37.1861 30.4887 27.5781 35.3668 40.0909 36.729
StdDesv 3.303 3.1793 3.3213 2.9945 2.4893 2.1203 3.3097 2.9579 3.5351
TKW
40.1
35.4
30.6
25.9
21.2
16.4
17BMS
17BSP100
17BSP67
18BI
18BMS
18BSP100
18DMS
19BMS
19BSP100

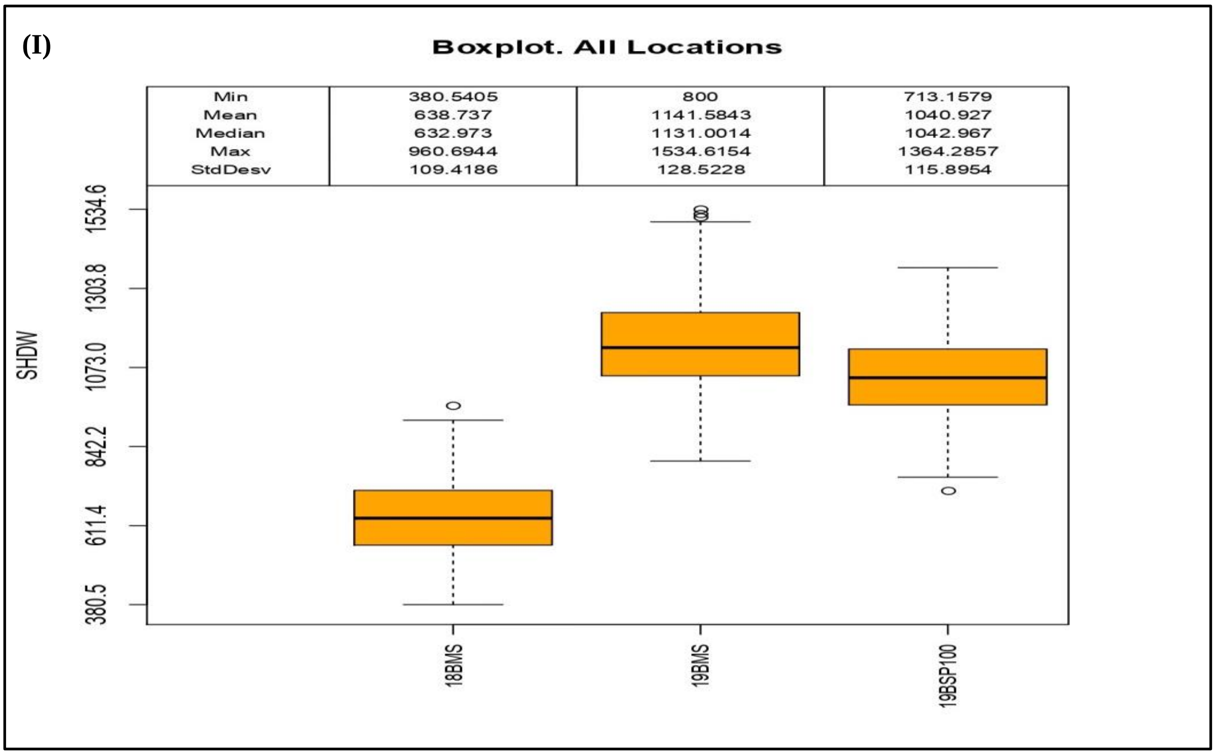
(I)
Boxplot. All Locations
Min 380.5405 800 713.1579
Mean 638.737 1141.5843 1040.927
Median 632.973 1131.0014 1042.967
Max 960.6944 1534.6154 1364.2857
StdDesv 109.4186 128.5228 115.8954
SHDW
1534.6
1303.8
1073.0
842.2
611.4
380.5
18BMS
19BMS
19BSP100

(J)

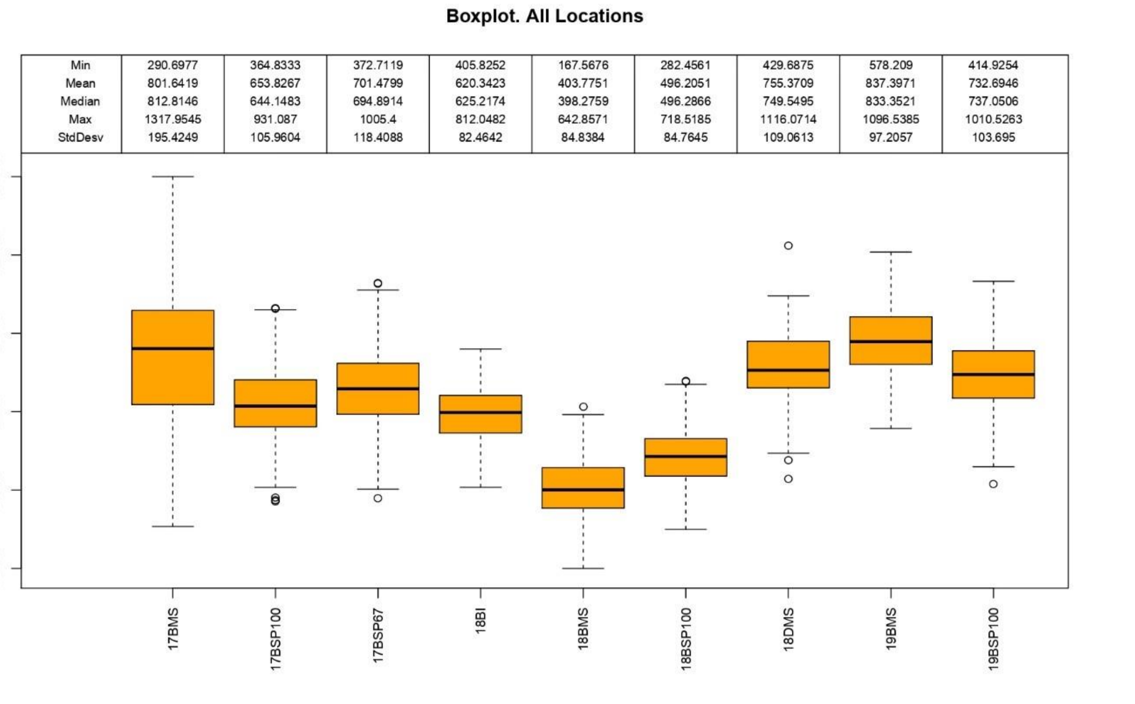
Boxplot. All Locations
Min 290.6977 364.8333 372.7119 405.8252 167.5676 282.4561 429.6875 578.209 414.9254
Mean 801.6419 653.8267 701.4799 620.3423 403.7751 496.2051 755.3709 837.3971 732.6946
Median 812.8146 644.1483 694.8914 625.2174 398.2759 496.2866 749.5495 833.3521 737.0506
Max 1317.9545 931.087 1005.4 812.0482 642.8571 718.5185 1116.0714 1096.5385 1010.5263
StdDesv 195.4249 105.9604 118.4088 82.4642 84.8384 84.7645 109.0613 97.2057 103.695
SHGW
1318.0
1087.9
857.8
627.7
397.6
167.6
17BMS
17BSP100
17BSP67
18BI
18BMS
18BSP100
18DMS
19BMS
19BSP100

(K)

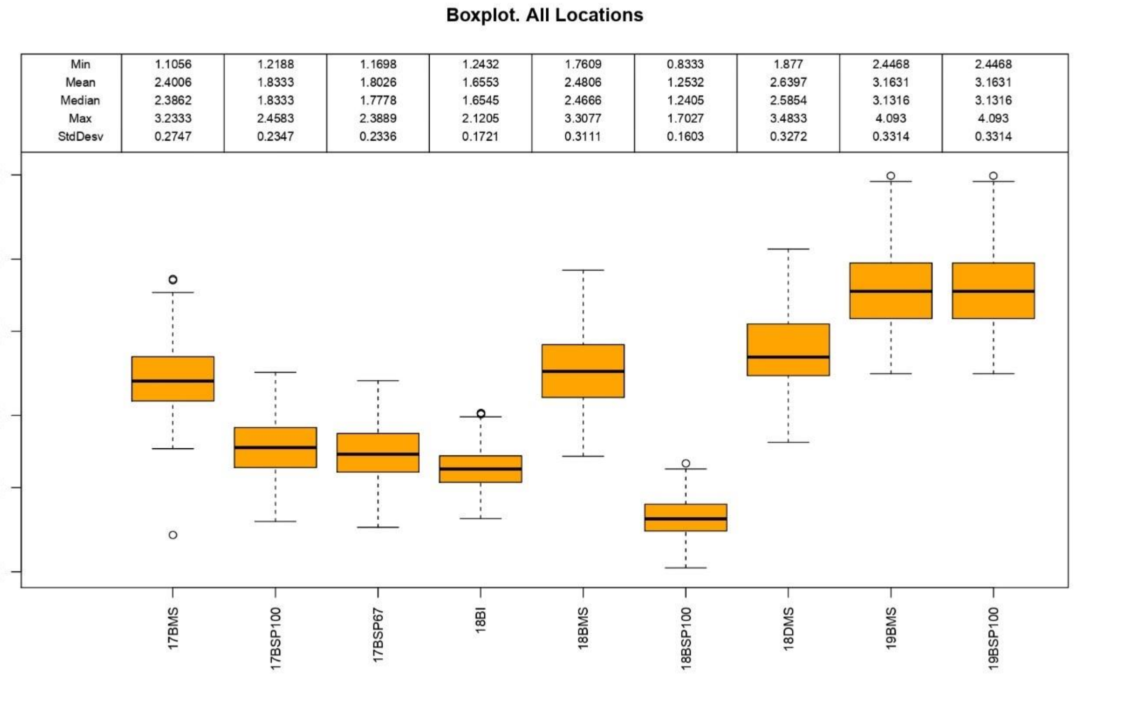
Boxplot. All Locations
Min 1.1056 1.2188 1.1698 1.2432 1.7609 0.8333 1.877 2.4468 2.4468
Mean 2.4006 1.8333 1.8026 1.6553 2.4806 1.2532 2.6397 3.1631 3.1631
Median 2.3862 1.8333 1.7778 1.6545 2.4666 1.2405 2.5854 3.1316 3.1316
Max 3.2333 2.4583 2.3889 2.1205 3.3077 1.7027 3.4833 4.093 4.093
StdDesv 0.2747 0.2347 0.2336 0.1721 0.3111 0.1603 0.3272 0.3314 0.3314
SCW
4.1
3.4
2.8
2.1
1.5
0.8
17BMS
17BSP100
17BSP67
18BI
18BMS
18BSP100
18DMS
19BMS
19BSP100

(L)

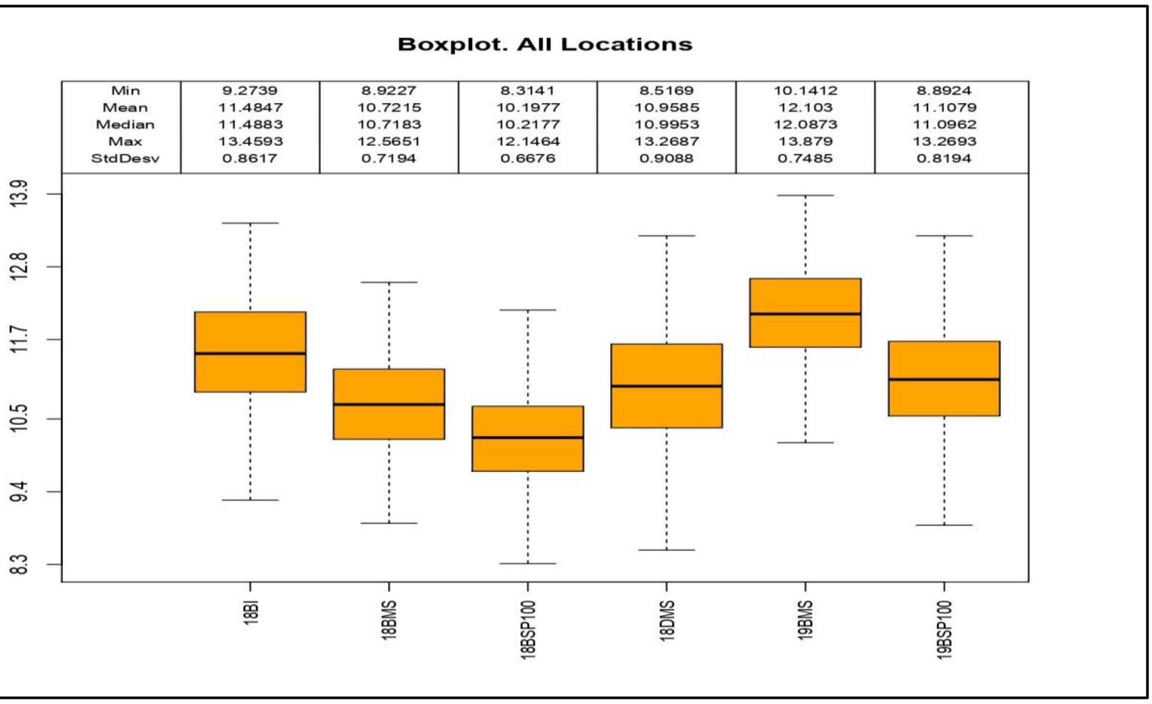
Boxplot. All Locations
Min 9.2739 8.9227 8.3141 8.5169 10.1412 8.8924
Mean 11.4847 10.7215 10.1977 10.9585 12.103 11.1079
Median 11.4883 10.7183 10.2177 10.9953 12.0873 11.0962
Max 13.4593 12.5651 12.1464 13.2687 13.879 13.2693
StdDesv 0.8617 0.7194 0.6676 0.9088 0.7485 0.8194
AREA
13.9
12.8
11.7
10.5
9.4
8.3
18BI
18BMS
18BSP100
18DMS
19BMS
19BSP100

(M)

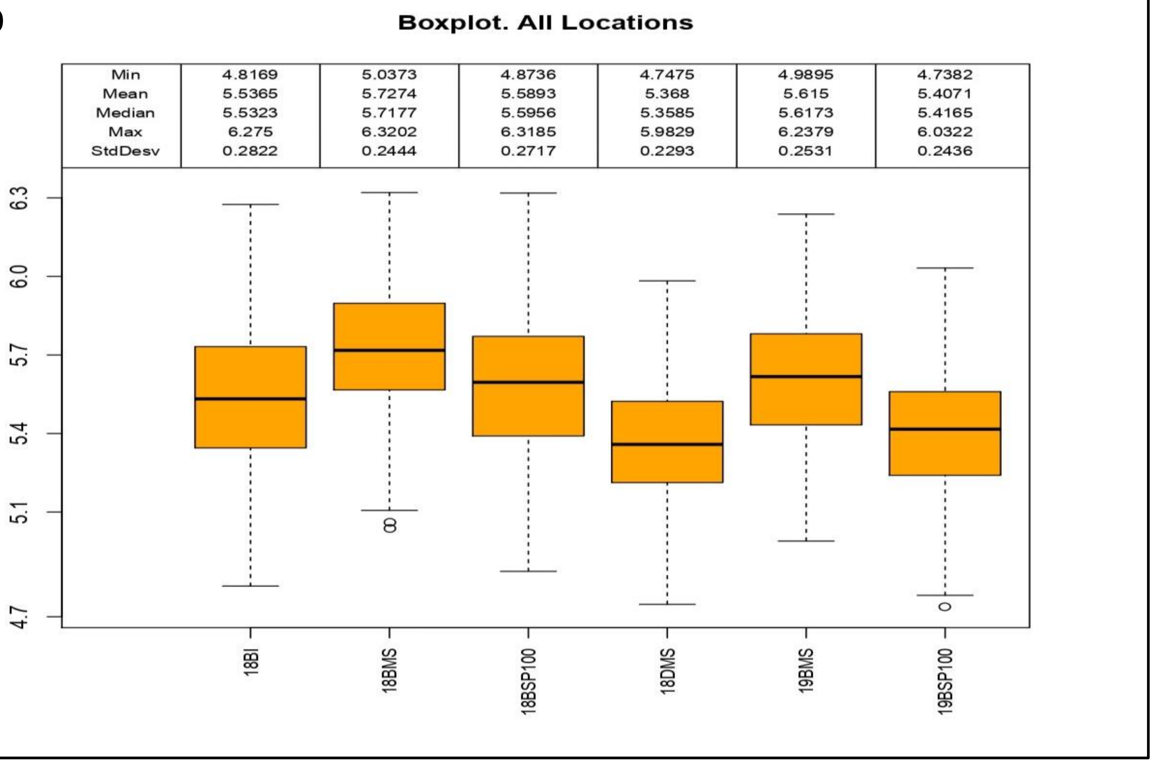
Boxplot. All Locations
Min 4.8169 5.0373 4.8736 4.7475 4.9895 4.7382
Mean 5.5365 5.7274 5.5893 5.368 5.615 5.4071
Median 5.5323 5.7177 5.5956 5.3585 5.6173 5.4165
Max 6.275 6.3202 6.3185 5.9829 6.2379 6.0322
StdDesv 0.2822 0.2444 0.2717 0.2293 0.2531 0.2436
KLEN
6.3
6.0
5.7
5.4
5.1
4.7
18BI
18BMS
18BSP100
18DMS
19BMS
19BSP100

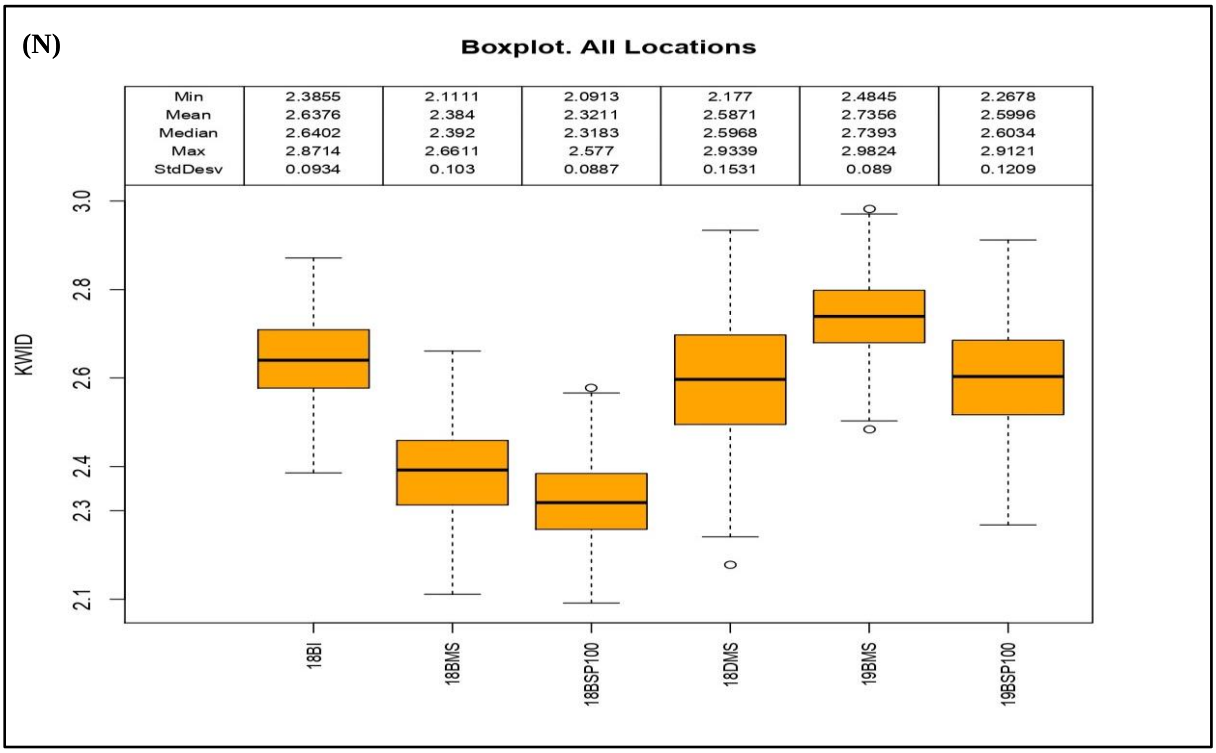
(N)
Boxplot. All Locations
Min 2.3855 2.1111 2.0913 2.177 2.4845 2.2678
Mean 2.6376 2.384 2.3211 2.5871 2.7356 2.5996
Median 2.6402 2.392 2.3183 2.5968 2.7393 2.6034
Max 2.8714 2.6611 2.577 2.9339 2.9824 2.9121
StdDesv 0.0934 0.103 0.0887 0.1531 0.089 0.1209
KWID
3.0
2.8
2.6
2.4
2.3
2.1
18BI
18BMS
18BSP100
18DMS
19BMS
19BSP100

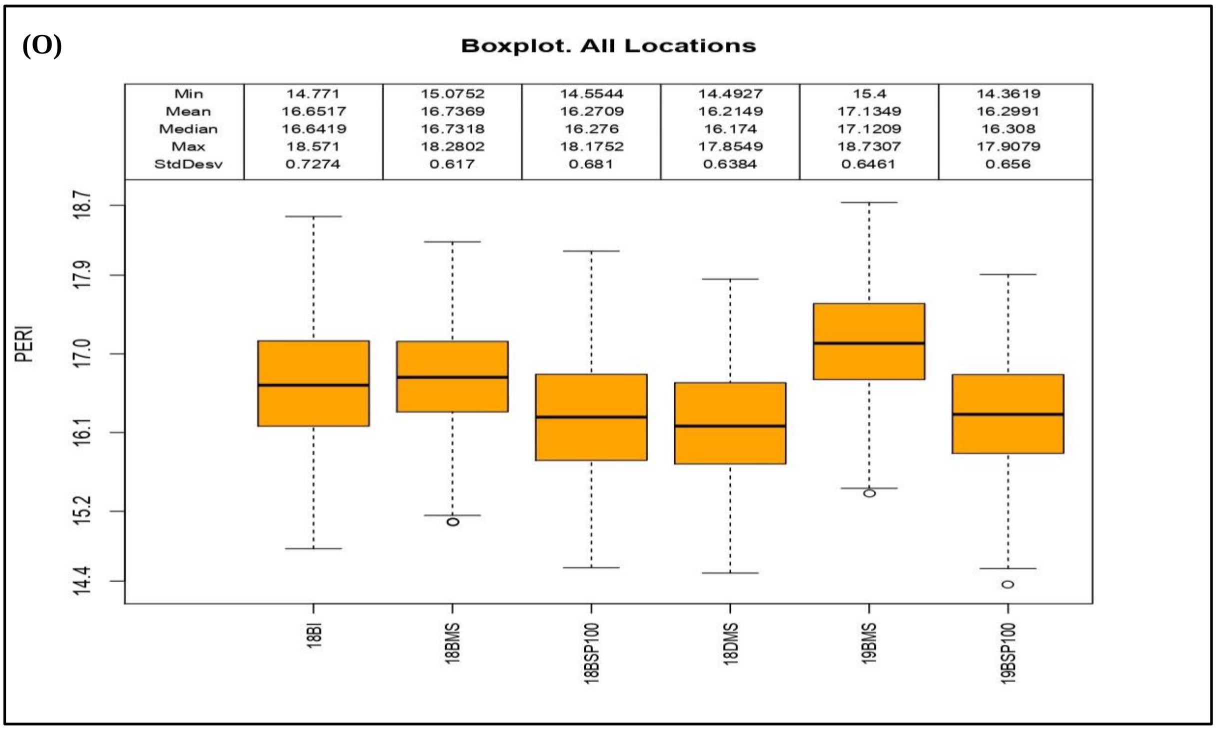
(O)
Boxplot. All Locations
Min 14.771 15.0752 14.5544 14.4927 15.4 14.3619
Mean 16.6517 16.7369 16.2709 16.2149 17.1349 16.2991
Median 16.6419 16.7318 16.276 16.174 17.1209 16.308
Max 18.571 18.2802 18.1752 17.8549 18.7307 17.9079
StdDesv 0.7274 0.617 0.681 0.6384 0.6461 0.656
PERI
18.7
17.9
17.0
16.1
15.2
14.4
18BI
18BMS
18BSP100
18DMS
19BMS
19BSP100

(P)

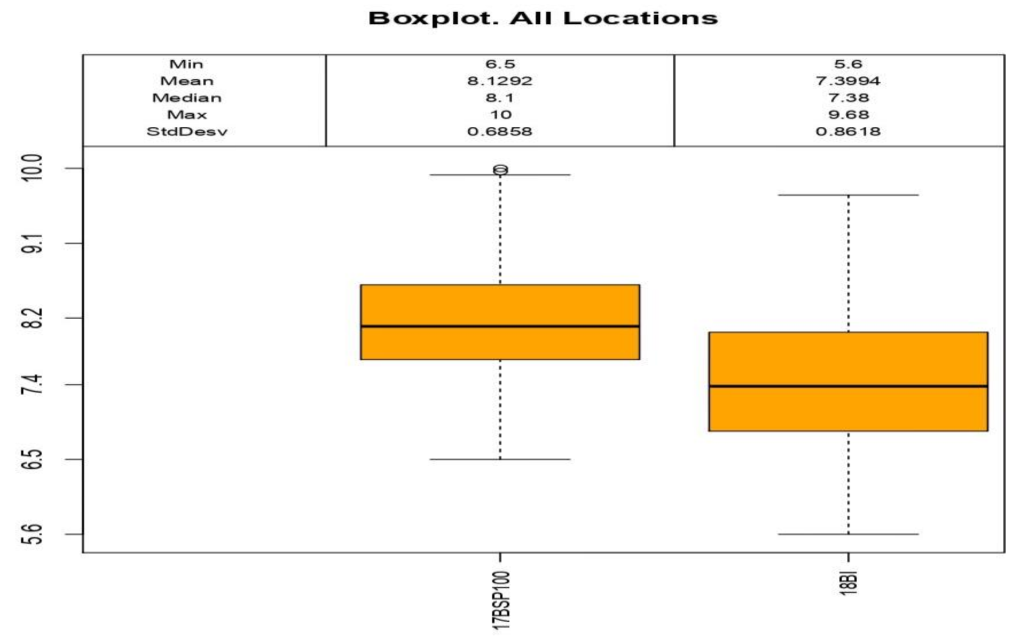

Boxplot. All Locations
Min 6.5 5.6
Mean 8.1292 7.3994
Median 8.1 7.38
Max 10 9.68
StdDesv 0.6858 0.8618
HL
10.0
9.1
8.2
7.4
6.5
5.6
17BSP100
18BI

(Q)

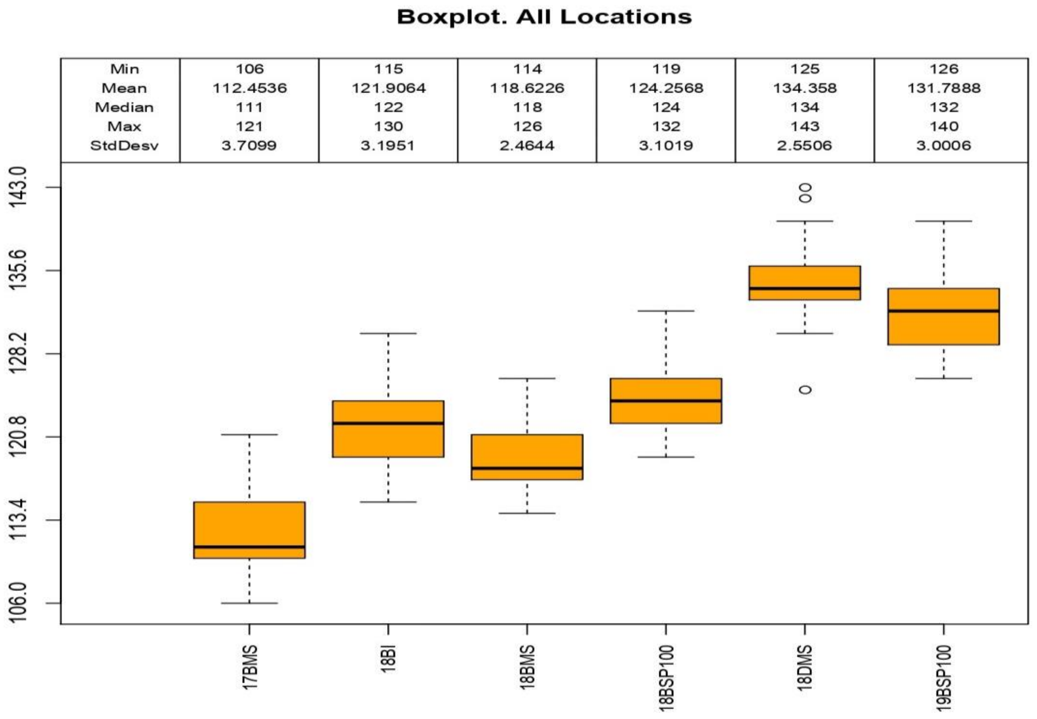

Boxplot. All Locations
Min 106 115 114 119 125 126
Mean 112.4536 121.9064 118.6226 124.2568 134.358 131.7888
Median 111 122 118 124 134 132
Max 121 130 126 132 143 140
StdDesv 3.7099 3.1951 2.4644 3.1019 2.5506 3.0006
HD
143.0
135.6
128.2
120.8
113.4
106.0
17BMS
18BI
18BMS
18BSP100
18DMS
19BSP100

(R)

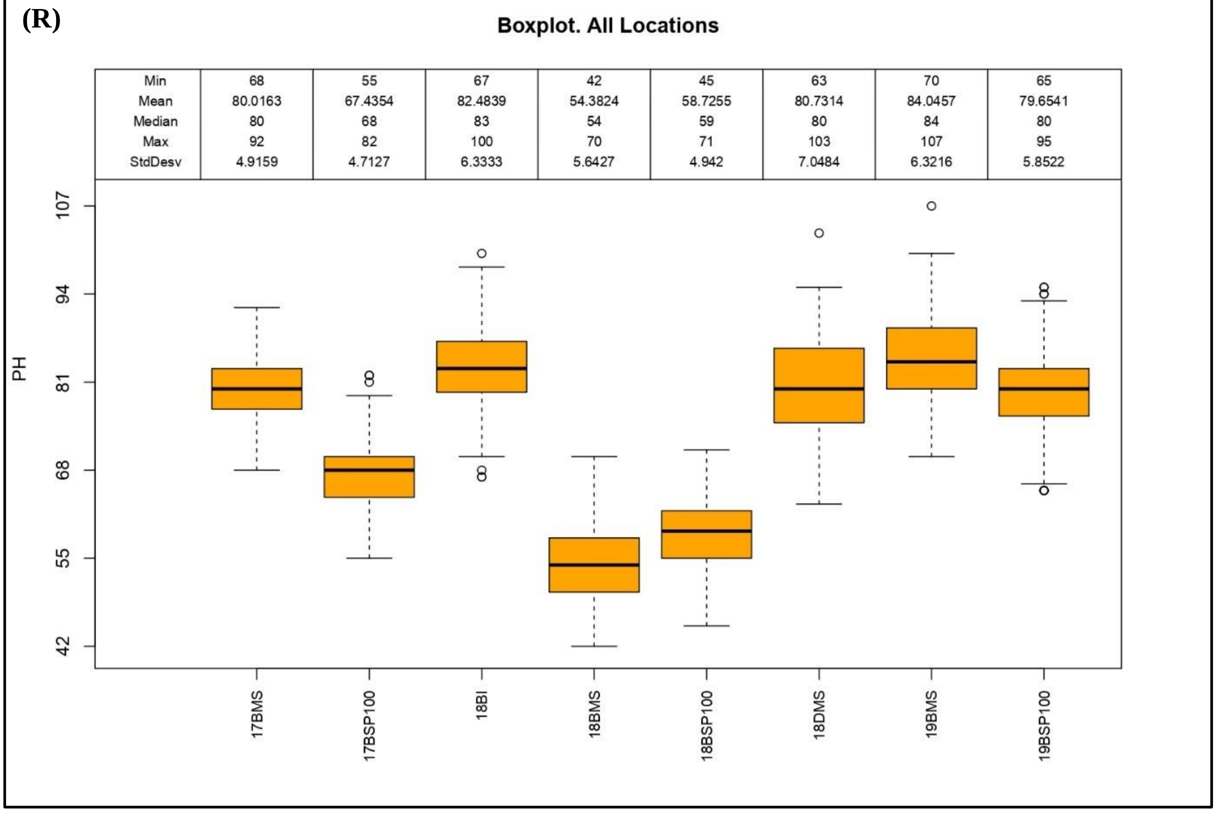

Boxplot. All Locations
Min 68 55 67 42 45 63 70 65
Mean 80.0163 67.4354 82.4839 54.3824 58.7255 80.7314 84.0457 79.6541
Median 80 68 83 54 59 80 84 80
Max 92 82 100 70 71 103 107 95
StdDesv 4.9159 4.7127 6.3333 5.6427 4.942 7.0484 6.3216 5.8522
PH
107
94
81
68
55
42
17BMS
17BSP100
18BI
18BMS
18BSP100
18DMS
19BMS
19BSP100

Supplemental figure S2: Dendrogram and biplot of yield, kernel, and agronomy traits, for megaenvironments classification. Environments: Texas A&M AgriLife Research stations in Bushland, TX, irrigated land in 2018 (18BI), Bushland middle school dryland in 2017, 2018, 2019 (17BMS, 18BMS, 19BMS). Bushland south pivot irrigated land in 2017 with 67% and 100% irrigation levels (17BSP67, 17BSP100), in 2018, 2019 with 100% irrigation level (18BSP100, 19BSP100). Chillicothe dryland in 2017 (17CH). Dumas irrigated in 2018 (18DMS). Noble Foundation Dupy Farm in 2018 (18NFD), Red River Research and Demonstration Farm in 2017, 2019 (17RRD, 19RRD). Mega-environments (MEs) for: A) YLD: ME1(18BI, 19BMS, 19BSP100), ME2(17BMS, 17CH, 19RRD), ME3(17RRD, 18BSP100, 18NFD); B) BMYLD: ME1(17BSP67, 18BSP100), ME2(17BMS, 17BSP100, 19BSP100); C) HI: ME1(18BI, 19BMS), ME2(17BSP67, 17BSP100, 19BSP100), ME3(17BMS, 18BSP100); D) KPS: ME1(17BMS, 17BSP100, 17BSP67, 19BSP100), ME2(18BI, 18BMS, 19BMS); E) SPM: ME1(19BMS, 19BSP100), ME2(17BMS, 18BI, 18BMS), ME3(17BSP67, 17BSP100); F) TKW: ME1(17BMS, 17BSP67, 17BSP100, 19BSP100), ME2(18BI, 18BMS, 19BMS); G) KLEN: ME1(19BMS, 19BSP100), ME2(18BI, 18BMS); H) KWID: ME1(18BI, 18BMS), ME2(19BMS, 19BSP100). Traits: A) Grain yield from whole plot (YLD); B) grain yield from biomass sample (BMYLD); C) harvest index (HI); D) kernel spike^{-1} (KPS); E) spike m^{-2} (SPM); F) thousand kernel weight (TKW); G) kernel length (KLEN); H) kernel width (KWID).

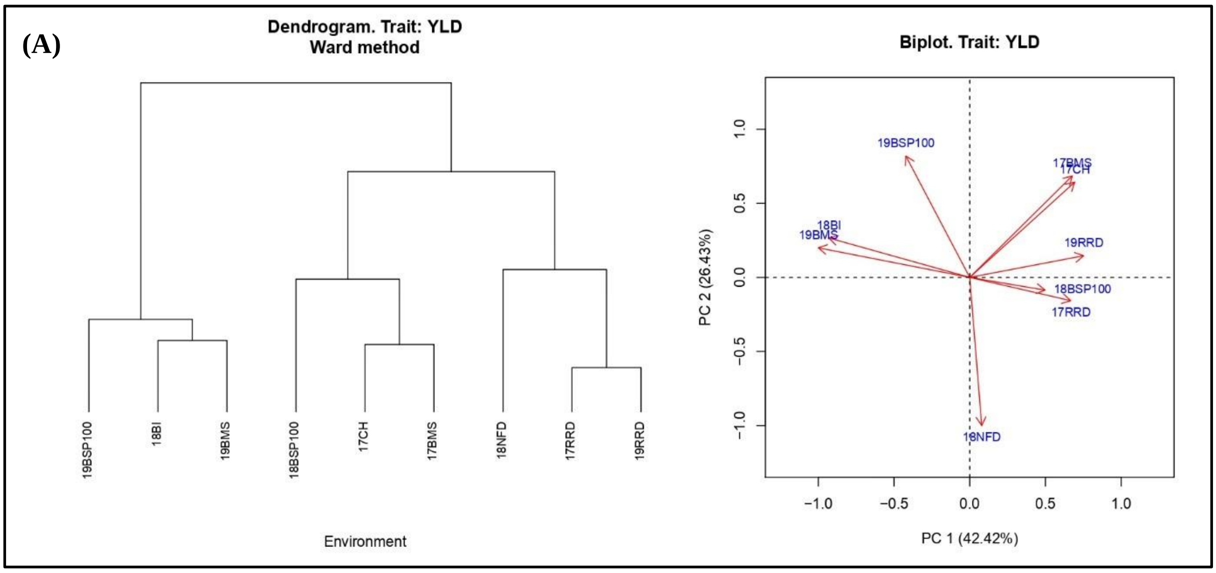

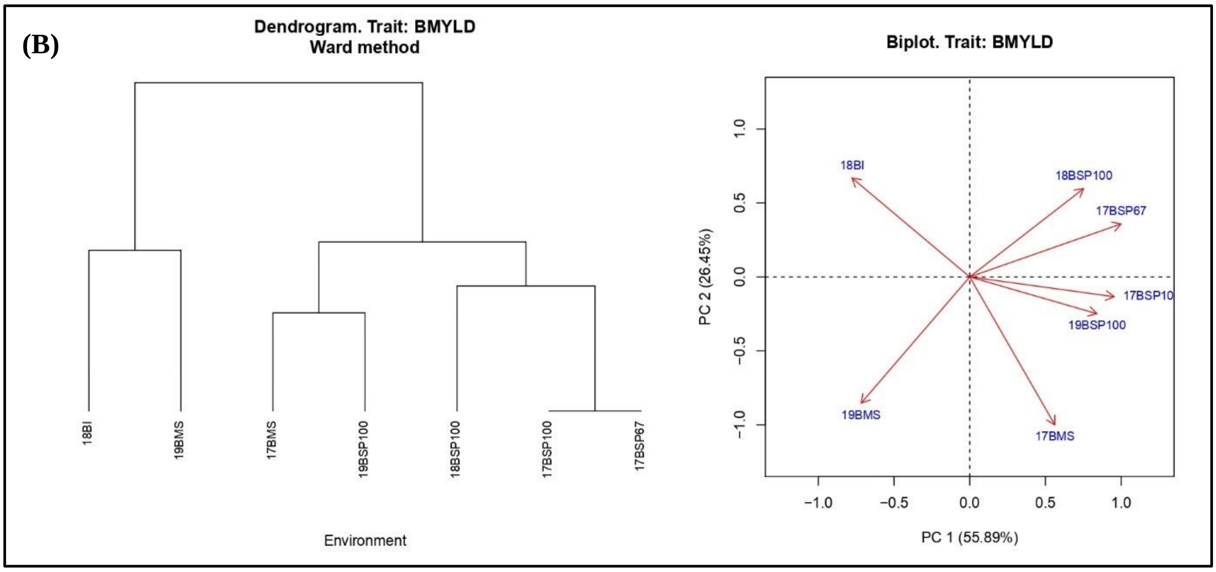

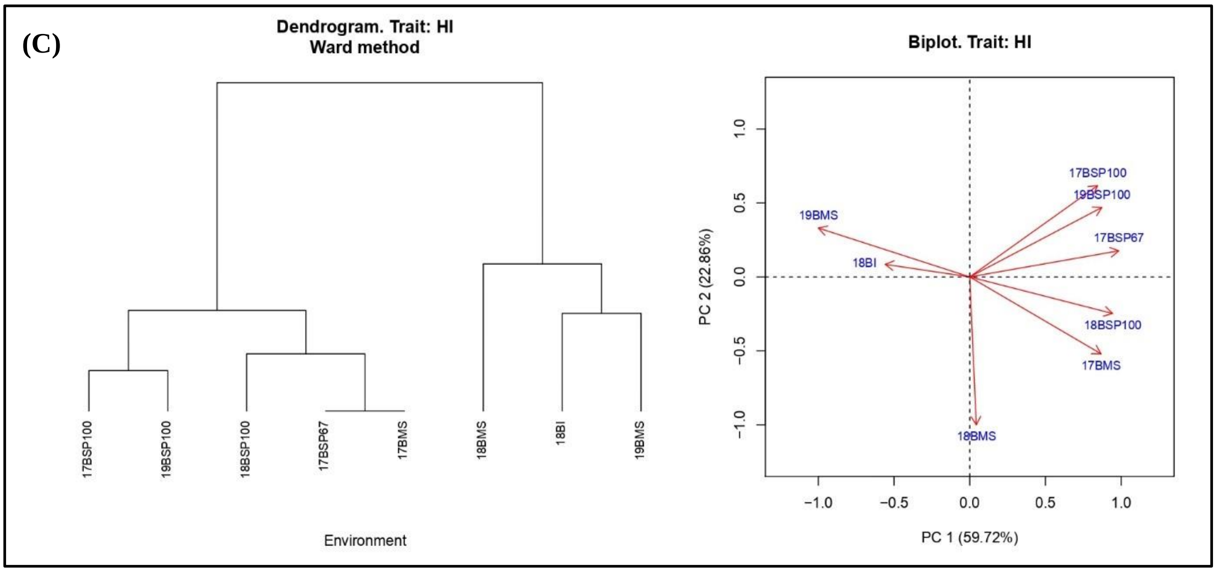
(C)
Dendrogram. Trait: HI
Ward method
17BSP100
19BSP100
18BSP100
17BSP67
17BMS
18BMS
18BI
19BMS
Environment
Biplot. Trait: HI
PC 2 (22.86%)
PC 1 (59.72%)
17BSP100
19BSP100
19BMS
17BSP67
18BI
18BSP100
17BMS
18BMS
−1.0
−0.5
0.0
0.5
1.0

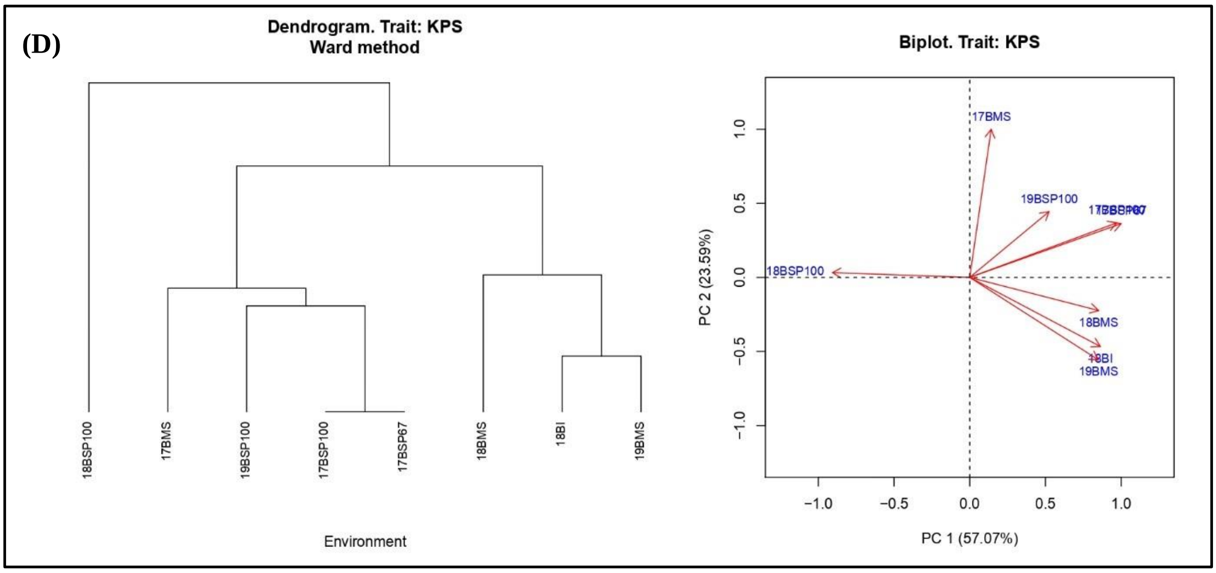
(D)
Dendrogram. Trait: KPS
Ward method
18BSP100
17BMS
19BSP100
17BSP100
17BSP67
18BMS
18BI
19BMS
Environment
Biplot. Trait: KPS
PC 2 (23.59%)
PC 1 (57.07%)
17BMS
19BSP100
18BSP100
18BMS
18BI
19BMS
−1.0
−0.5
0.0
0.5
1.0

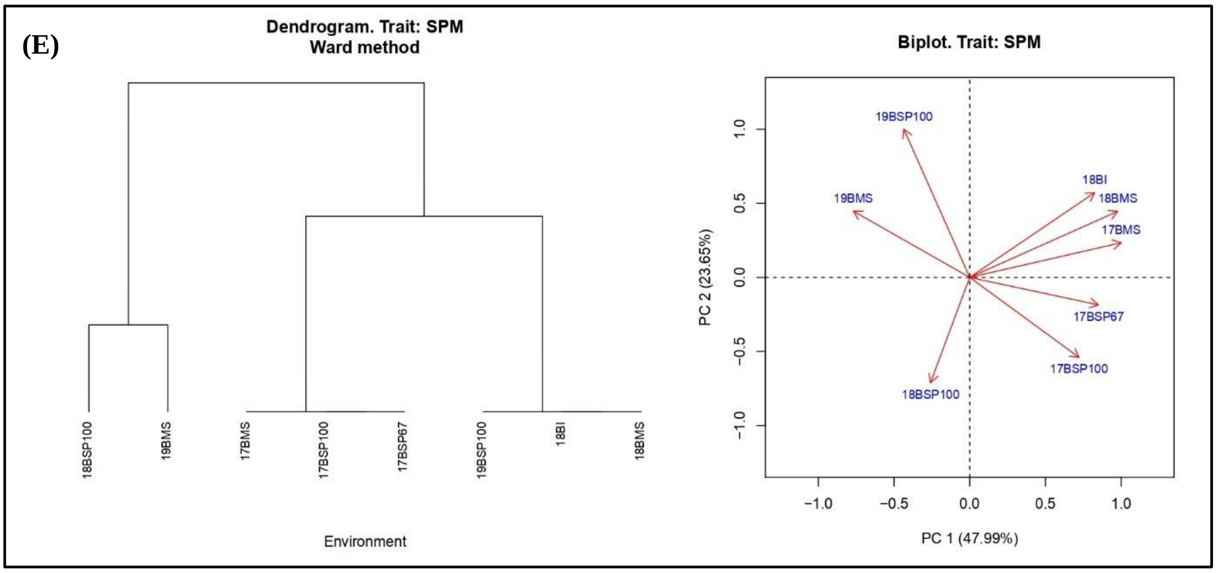
(E)
Dendrogram. Trait: SPM
Ward method
18BSP100
19BMS
17BMS
17BSP100
17BSP67
19BSP100
18BI
18BMS
Environment
Biplot. Trait: SPM
PC 2 (23.65%)
PC 1 (47.99%)
19BSP100
18BI
19BMS
18BMS
17BMS
17BSP67
17BSP100
18BSP100
−1.0
−0.5
0.0
0.5
1.0

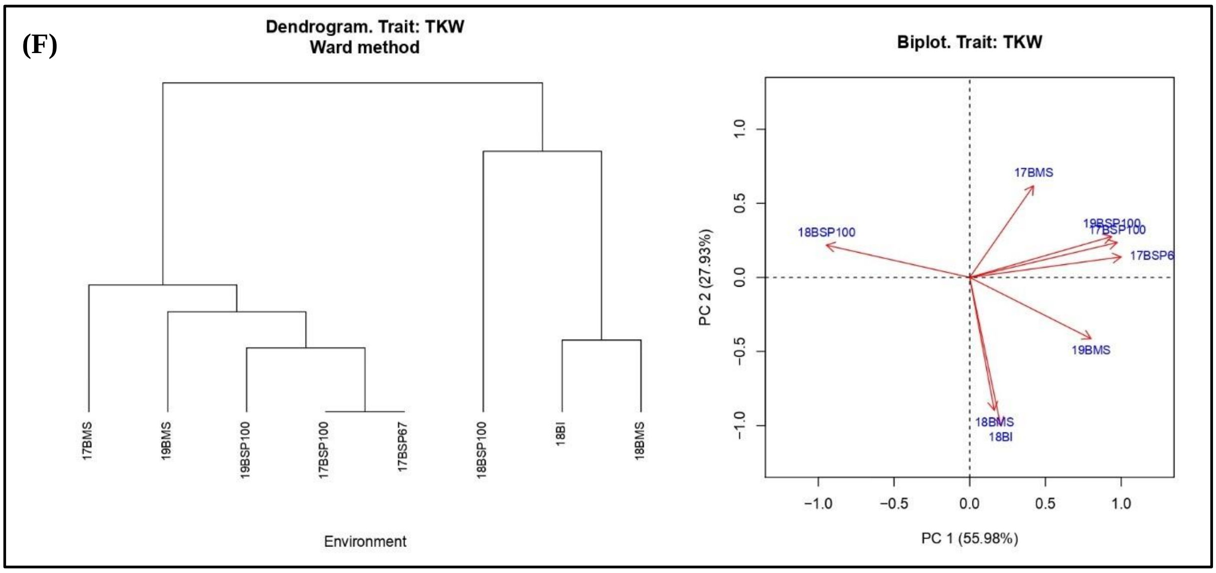
(F)
Dendrogram. Trait: TKW
Ward method
17BMS
19BMS
19BSP100
17BSP100
17BSP67
18BSP100
18BI
18BMS
Environment
Biplot. Trait: TKW
17BMS
18BSP100
19BSP100
17BSP100
17BSP6
19BMS
18BMS
18BI
PC 2 (27.93%)
PC 1 (55.98%)

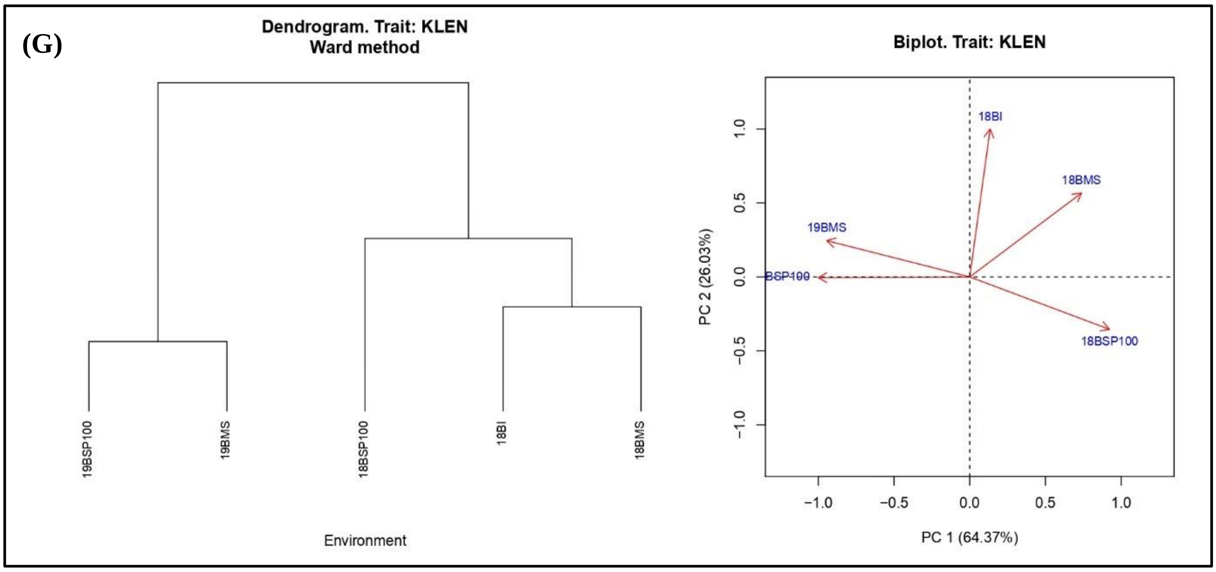
(G)
Dendrogram. Trait: KLEN
Ward method
19BSP100
19BMS
18BSP100
18BI
18BMS
Environment
Biplot. Trait: KLEN
18BI
18BMS
19BMS
BSP100
18BSP100
PC 2 (26.03%)
PC 1 (64.37%)

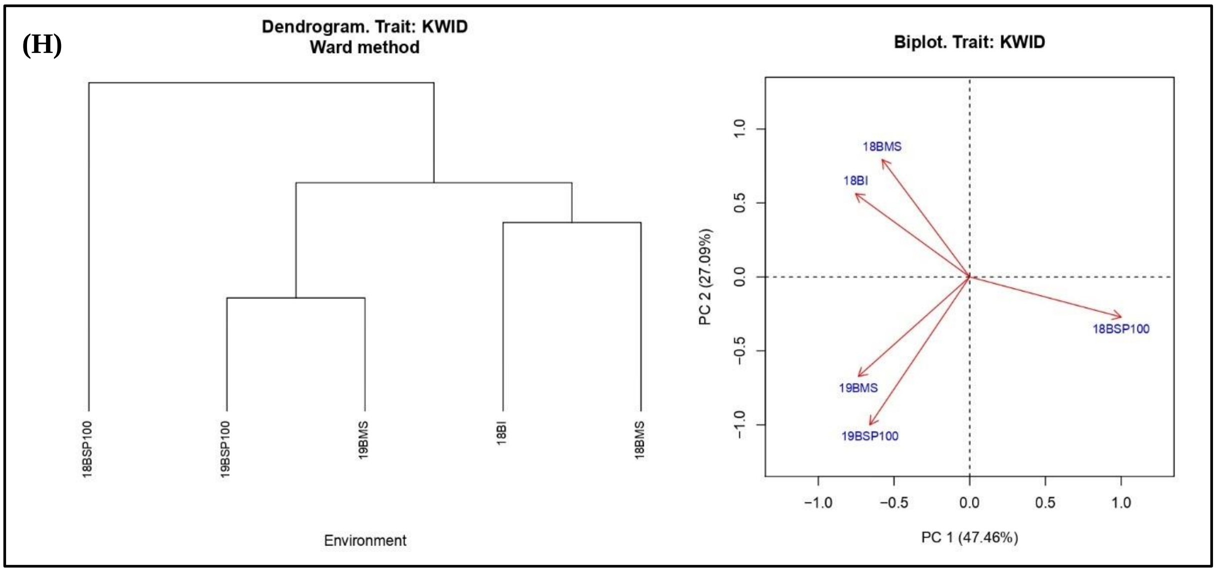
(H)
Dendrogram. Trait: KWID
Ward method
18BSP100
19BSP100
19BMS
18BI
18BMS
Environment
Biplot. Trait: KWID
18BMS
18BI
18BSP100
19BMS
19BSP100
PC 2 (27.09%)
PC 1 (47.46%)

Supplemental figure S3: SNP density maps. (A) Based on genetic distance (cM) by linkage groups; (B) Based on physical distance (Mbps) by linkage groups; (C) Based on physical distance (Mbps) by chromosomes.

(A)

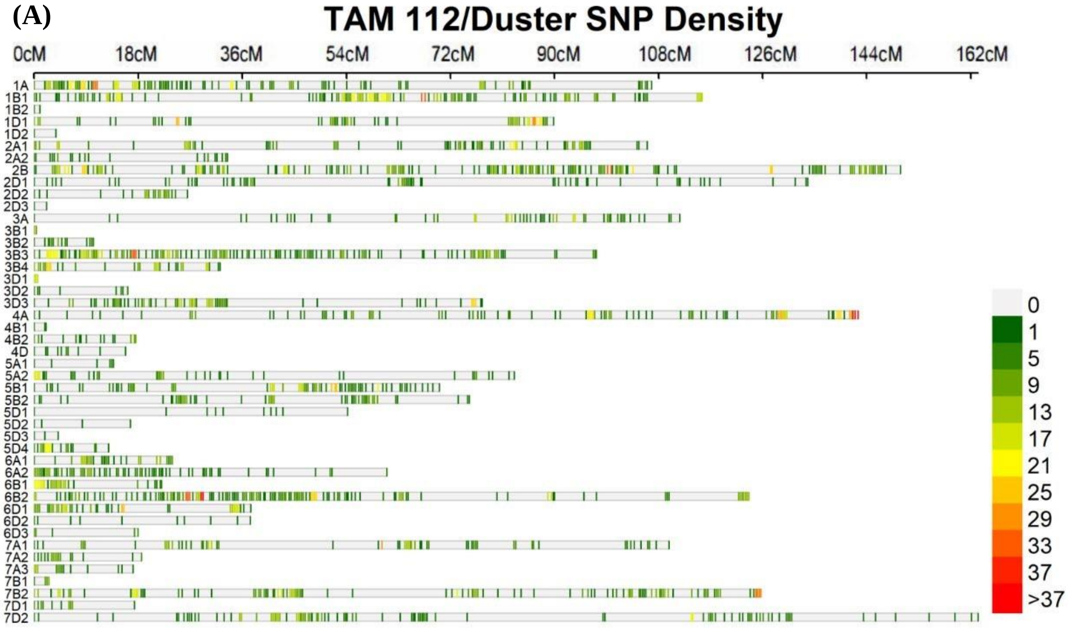

(B)

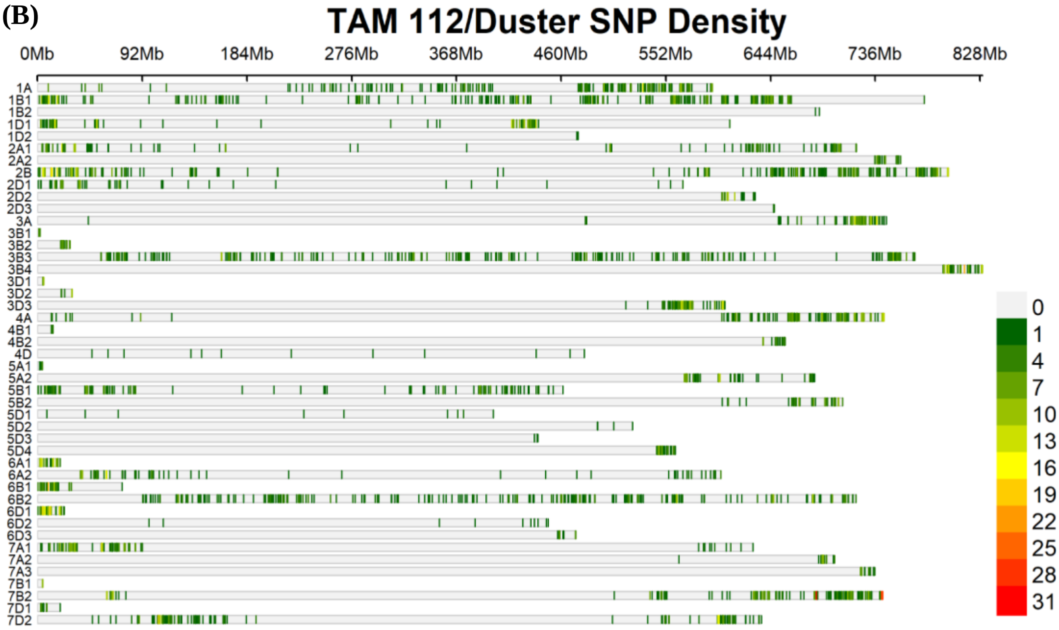

(C)

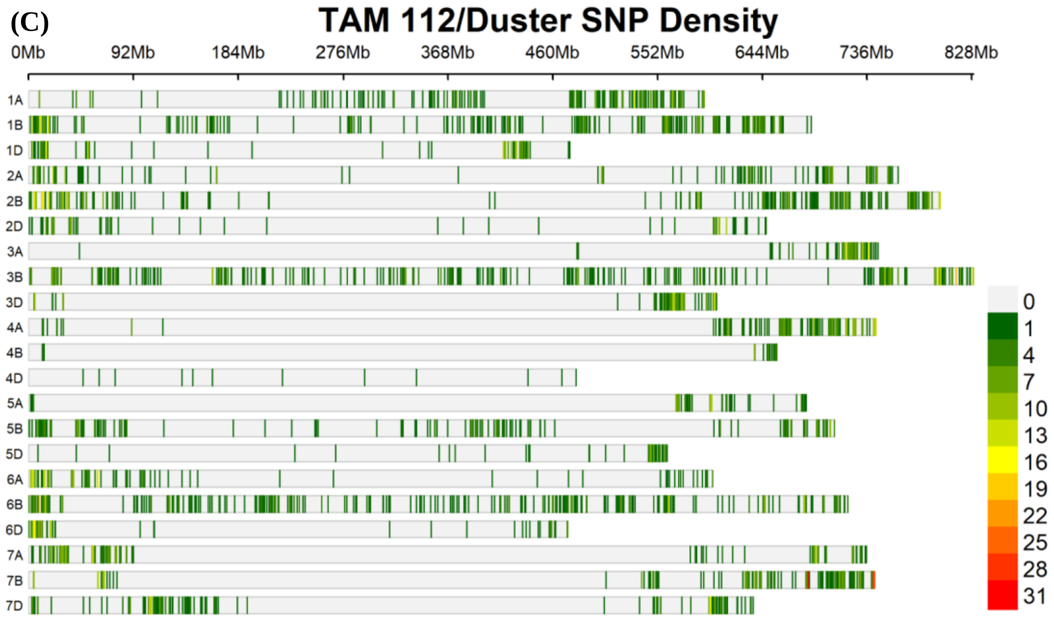

Supplemental figure S4: Whole genome significance profile of LOD score and additive effect for yield, seed, and agronomy traits quantitative trait loci under individual environment. For the whole panel, Xaxis shows the genetic position of markers from 45 linkage groups (cM). Chromosome segment name shows on top of graph, traits are partitioned with different color. The upper graph shows the LOD score on the Yaxis, with dashed line as LOD threshold. The lower graph shows the additive effect on the Y axis, the positive additive effect has favorable allele from TAM 112, the negative additive effect has favorable allele from Duster. Traits: 1) grain yield form whole plot (YLD, g m^{-2}); 2) dry weight of biomass sample (BM, g m^{-2}); 3) grain yield from biomass sample (BMYLD, g m^{-2}); 4) test weight (TW, Kg m^{-3}); 5) harvest index (HI, %); 6) kernel $spike^{-1}$ (KPS, kernel $spike^{-1}$); 7) spike m^{-2} (SPM, spike m^{-2}); 8) thousand kernel weight (TKW, g); 9) single head dry weight (SHDW, mg); 10) single head grain weight (SHGW, mg); 11) single culm weight (SCW, g); 12) kernel area (AREA, mm^2); 13) kernel perimeter (PERI, mm); 14) kernel length (KLEN, mm); 15) kernel width (KWID, mm); 16) spike length (SL, cm); 17) heading date (HD, days); 18) plant height (PH, cm). Individual environments: A) 17BMS; B) 17BSP67; C) 17BSP100; D) 17CH; E) 17RRD; F) 18BI; G) 18BMS; H) 18BSP100; I) 18DMS; J) 18NDF; K) 19BMS; L) 19BSP100; M) 19RRD.

(A)

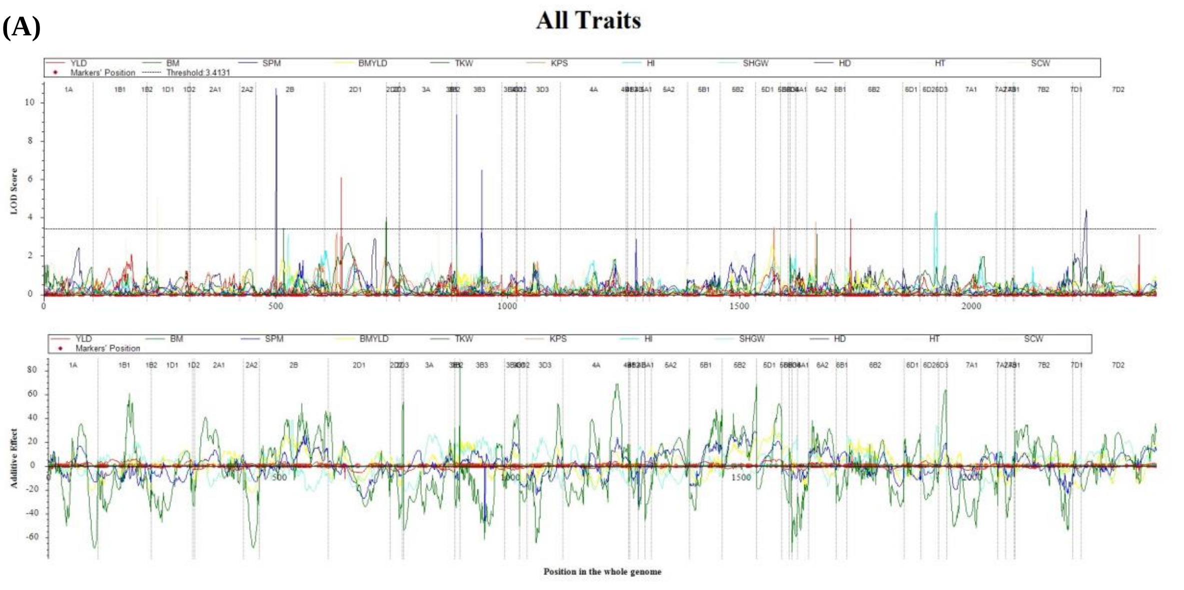
All Traits
YLD
BM
SPM
BMYLD
TKW
KPS
HI
SHGW
HD
HT
SCW
Markers' Position
Threshold:3.4131
LOD Score
Additive Effect
Position in the whole genome

(B)

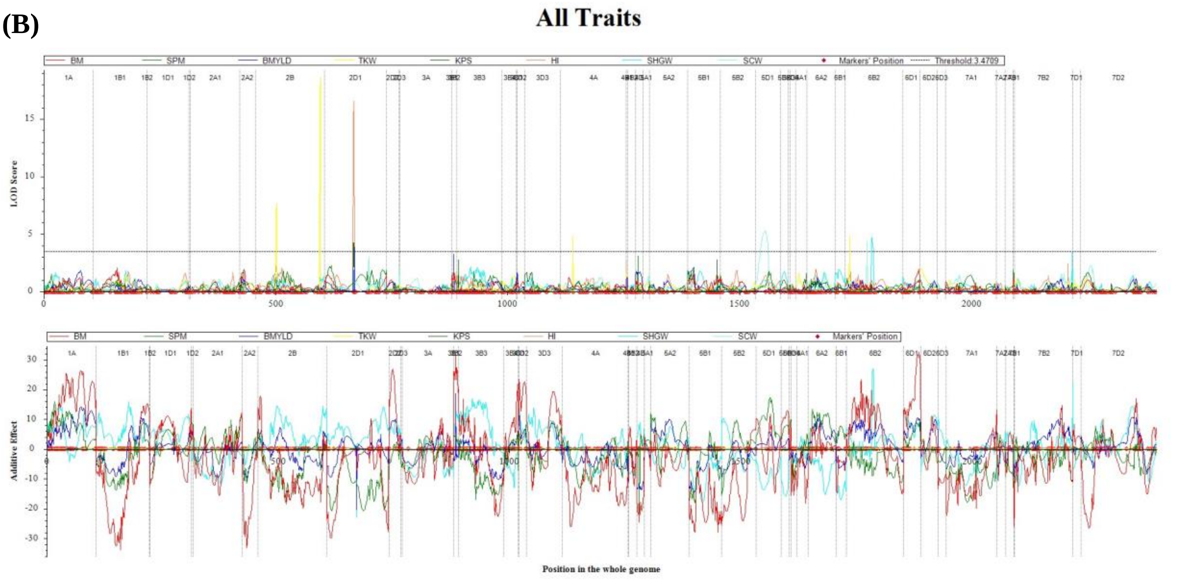
All Traits
BM
SPM
BMYLD
TKW
KPS
HI
SHGW
SCW
Markers' Position
Threshold:3.4709
LOD Score
Additive Effect
Position in the whole genome

(C)

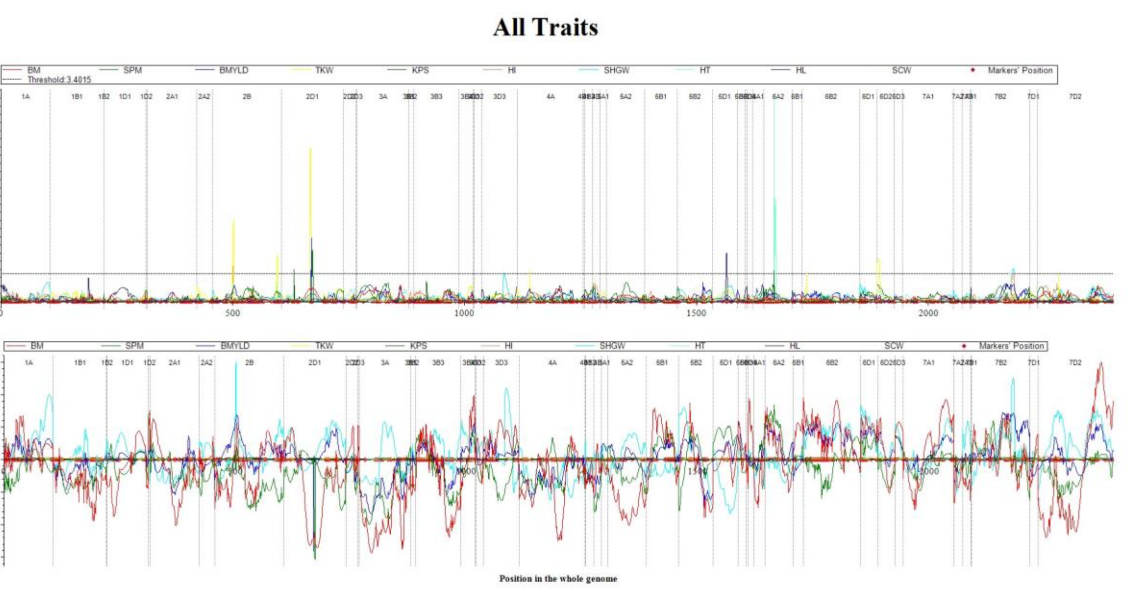

All Traits
BM
SPM
BMYLD
TKW
KPS
HI
SHGW
HT
HL
SCW
Markers' Position
Threshold:3.4015
LOD Score
Additive Effect
Position in the whole genome

(D)

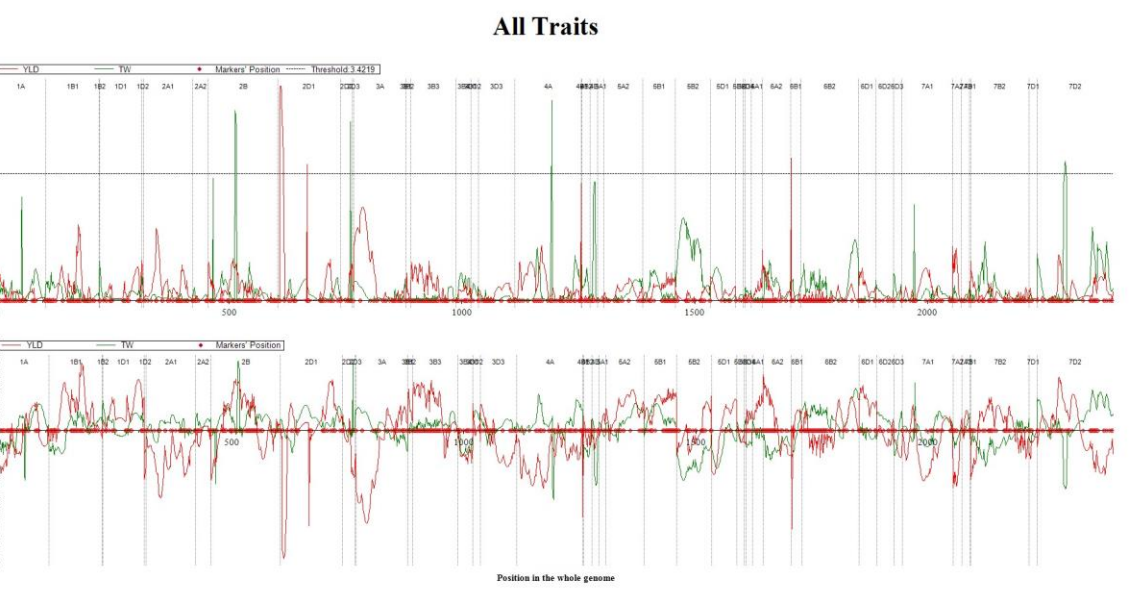

All Traits
YLD
TW
Markers' Position
Threshold:3.4219
LOD Score
Additive Effect
Position in the whole genome

(E)

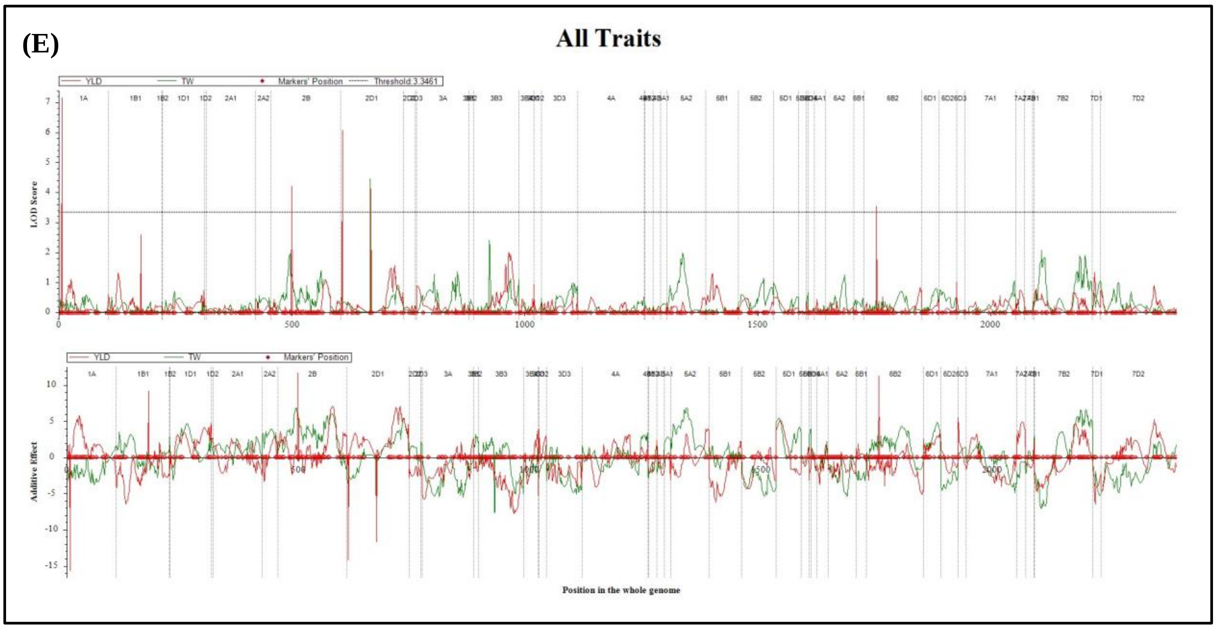
All Traits
YLD
TW
Markers' Position
Threshold:3.3461
LOD Score
Additive Effect
Position in the whole genome

(F)

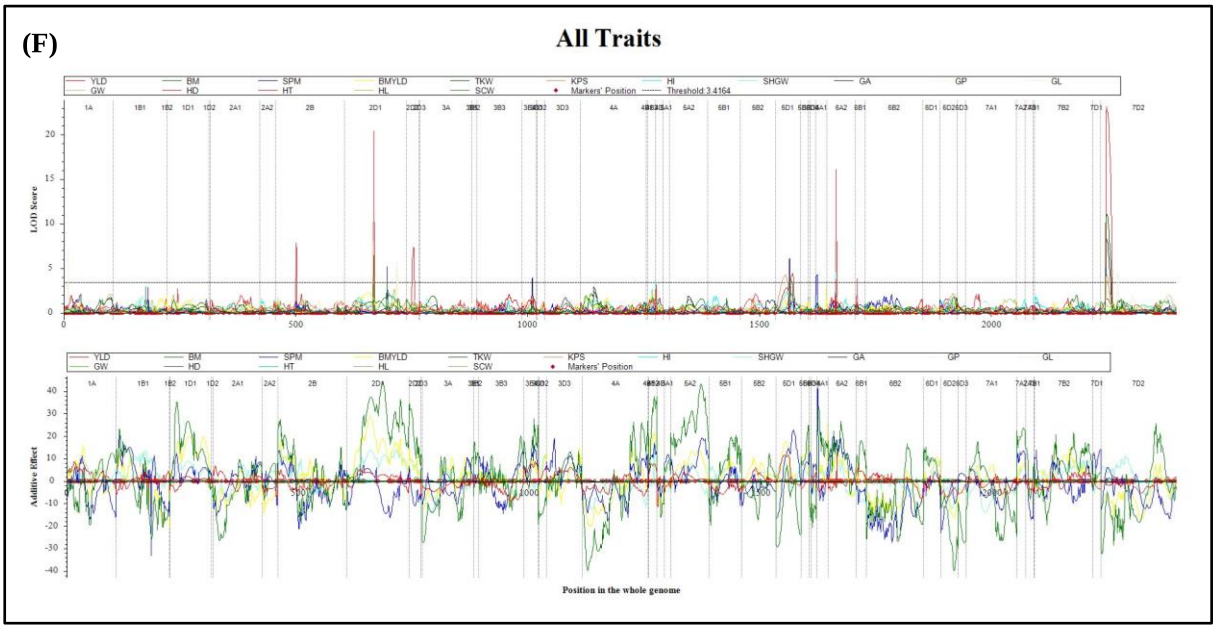
All Traits
YLD
BM
SPM
BMYLD
TKW
KPS
HI
SHGW
GA
GP
GL
GW
HD
HT
HL
SCW
Markers' Position
Threshold:3.4164
LOD Score
Additive Effect
Position in the whole genome

(G)

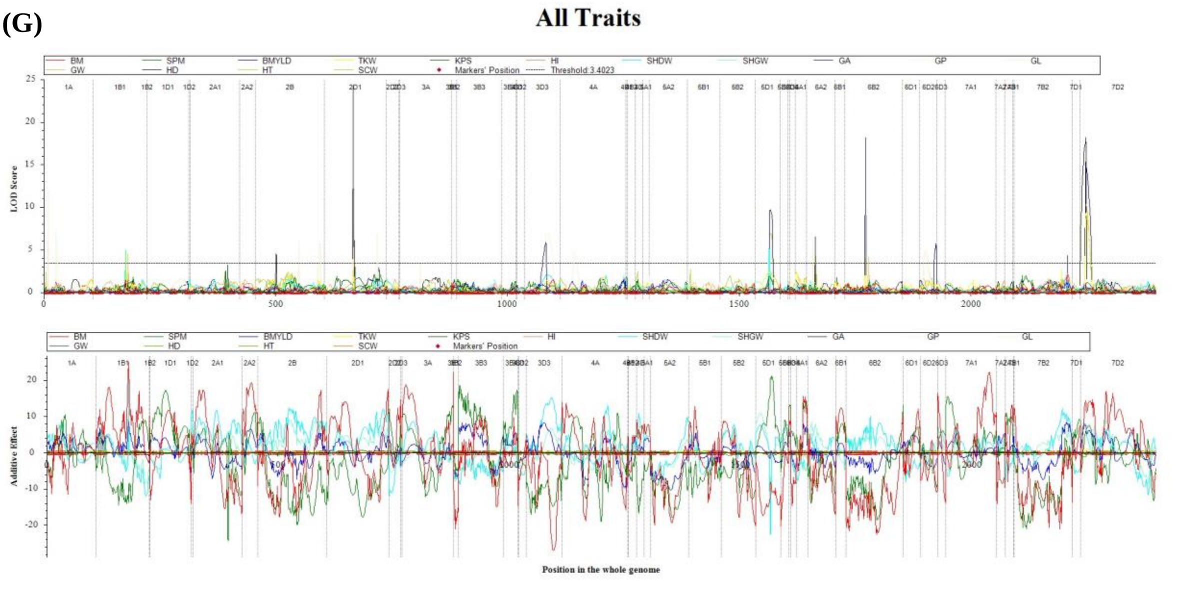
All Traits
BM
SPM
BMYLD
TKW
KPS
HI
SHDW
SHGW
GA
GP
GL
GW
HD
HT
SCW
Markers' Position
Threshold:3.4023
LOD Score
Additive Effect
Position in the whole genome

(H)

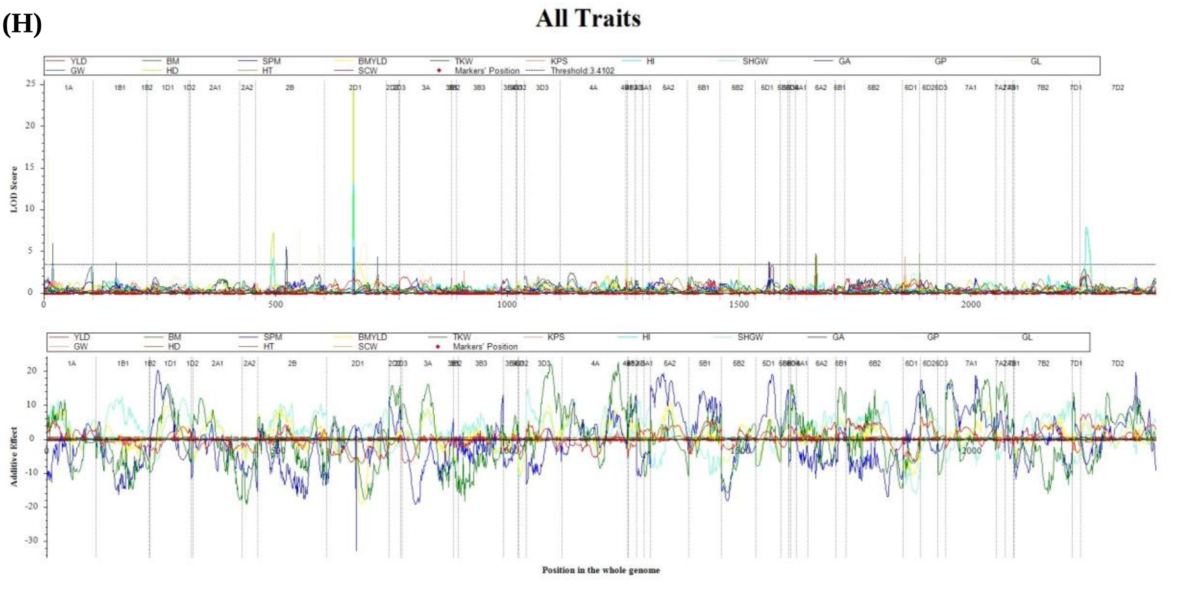
All Traits
YLD
BM
SPM
BMYLD
TKW
KPS
HI
SHGW
GA
GP
GL
GW
HD
HT
SCW
Markers' Position
Threshold:3.4102
LOD Score
Additive Effect
Position in the whole genome

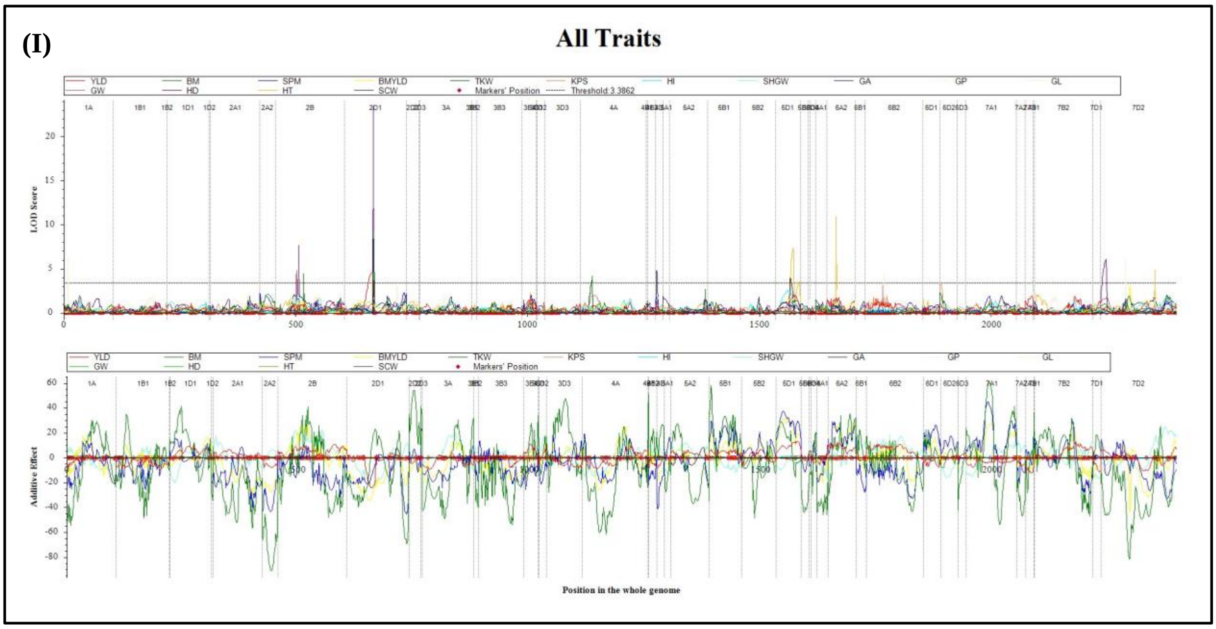
(I)
All Traits
YLD BM SPM BMYLD TKW KPS HI SHGW GA GP GL
GW HD HT SCW Markers' Position Threshold:3.3862
LOD Score
Additive Effect
Position in the whole genome

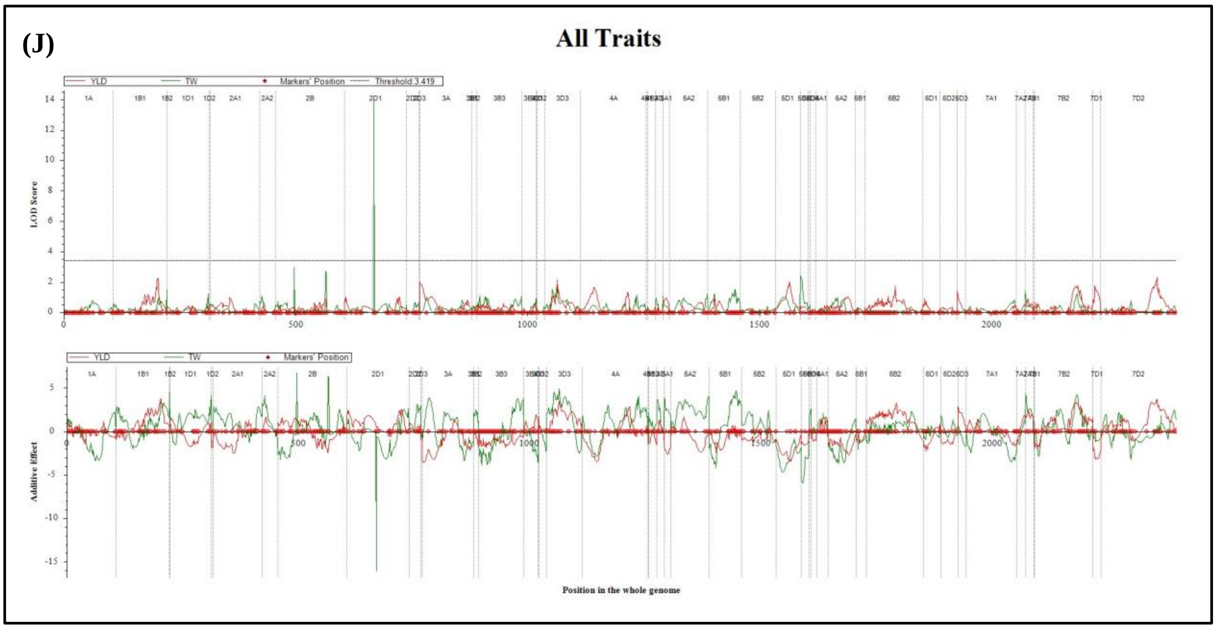
(J)
All Traits
YLD TW Markers' Position Threshold:3.419
LOD Score
Additive Effect
Position in the whole genome

(K)

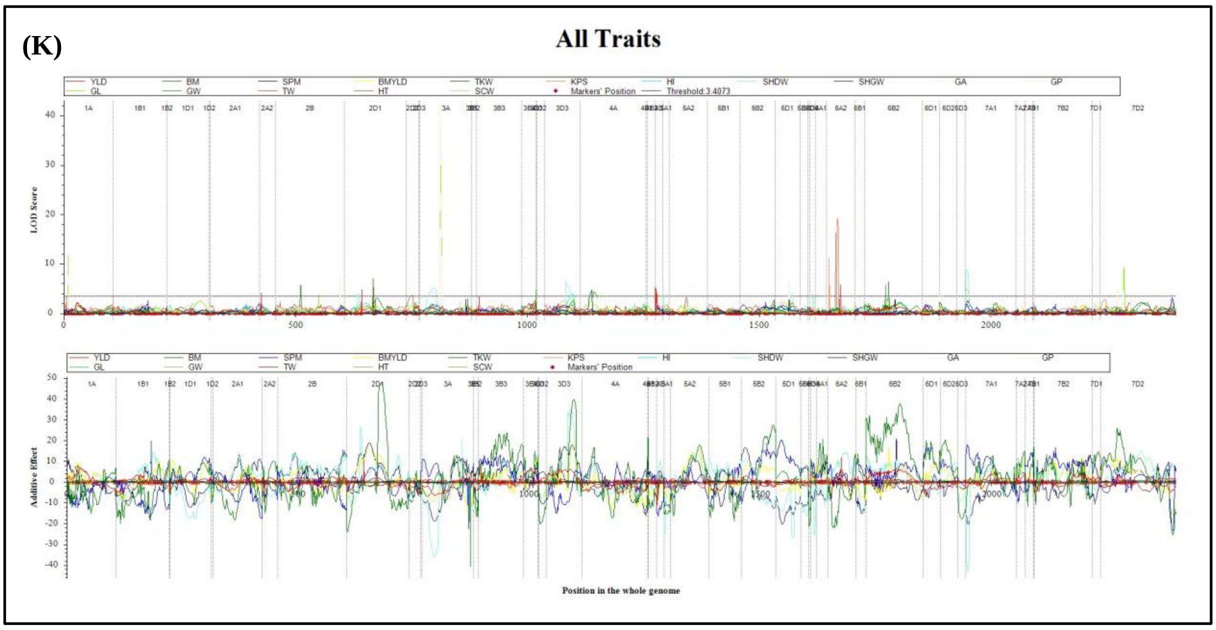
All Traits
YLD BM SPM BMYLD TKW KPS HI SHDW SHGW GA GP
GL GW TW HT SCW Markers' Position Threshold:3.4073
LOD Score
Additive Effect
Position in the whole genome

(L)

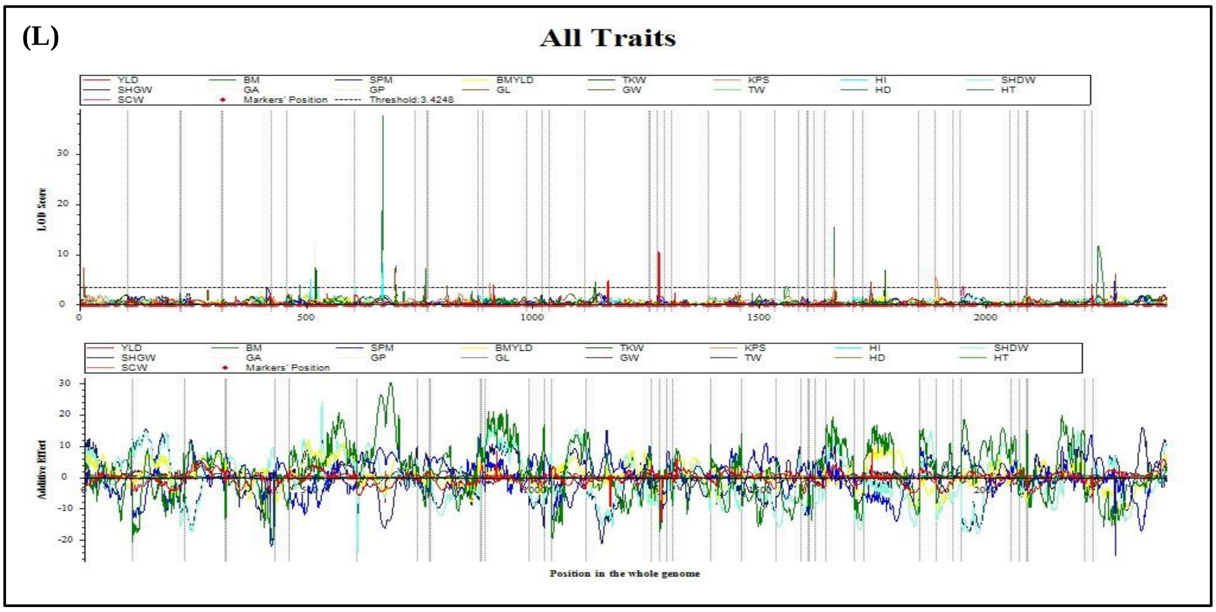
All Traits
YLD BM SPM BMYLD TKW KPS HI SHDW
SHGW GA GP GL GW TW HD HT
SCW Markers' Position Threshold:3.4248
LOD Score
Additive Effect
Position in the whole genome

Supplemental figure S5: The whole genome significance profile of LOD(A), LOD(AbyE) score and additive effects for yield, seed, and agronomy traits quantitative trait loci across all individual environments. For the whole panel, X-axis shows the genetic position of markers from 45 linkage groups (cM), chromosome segment name shows on top of graph, traits are partitioned with different color. The upper graph shows the LOD score on the Y-axis, the additive effect LOD(A) shows with red line, the additive by environment LOD(AbyE) shows with green color. The lower graph shows the additive effect on the Y axis, the positive additive effect has favorable allele from TAM 112, the negative additive effect has favorable allele from Duster. Traits: A) grain yield form whole plot (YLD, g m^{-2}); B) dry weight of biomass sample (BM, g m^{-2}); C) grain yield from biomass sample (BMYLD, g m^{-2}); D) test weight (TW, Kg m^{-3}); E) harvest index (HI, %); F) kernel $spike^{-1}$ (KPS, kernel $spike^{-1}$); G) spike m^{-2} (SPM, spike m^{-2}); H) thousand kernel weight (TKW, g); I) single head dry weight (SHDW, mg); J) single head grain weight (SHGW, mg);
K) single culm weight (SCW, g); L) kernel area (AREA, mm^2); M) kernel perimeter (PERI, mm); N) kernel length (KLEN, mm); O) kernel width (KWID, mm); P) spike length (SL, cm); Q) heading date (HD, days); R) plant height (PH, cm).

(A)

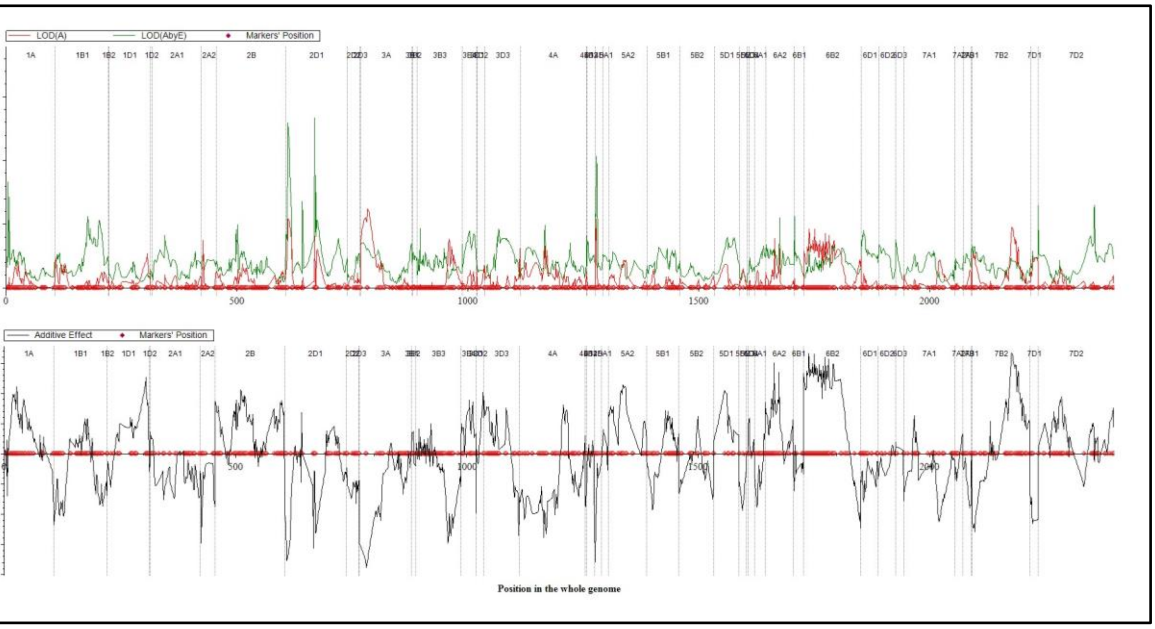

(B)

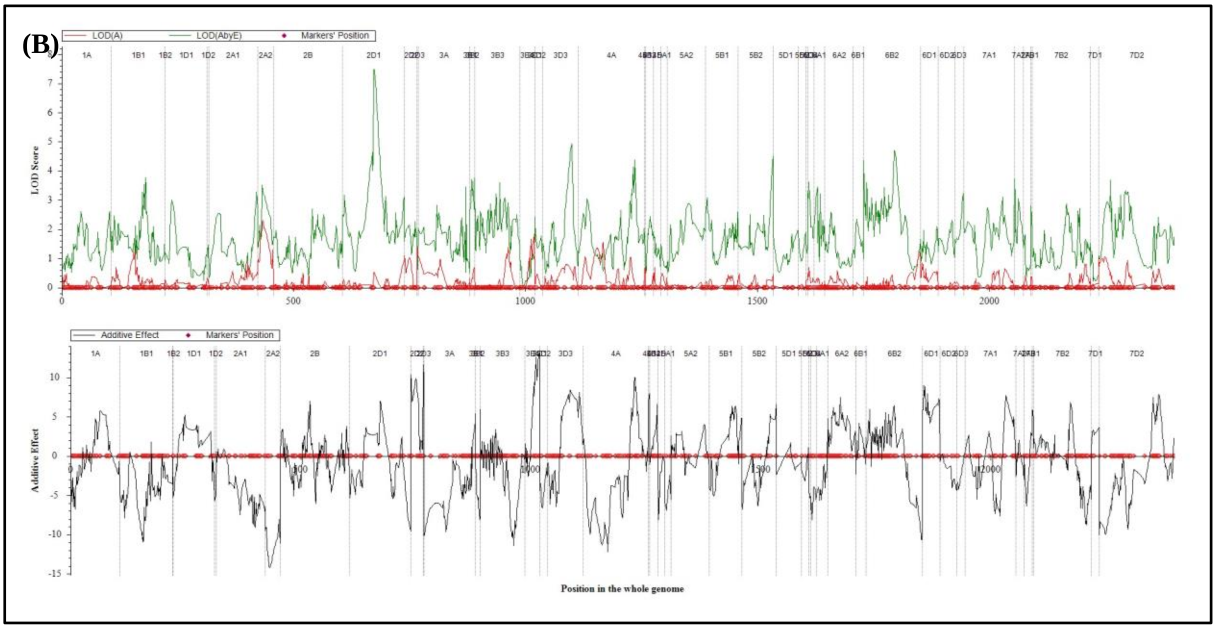

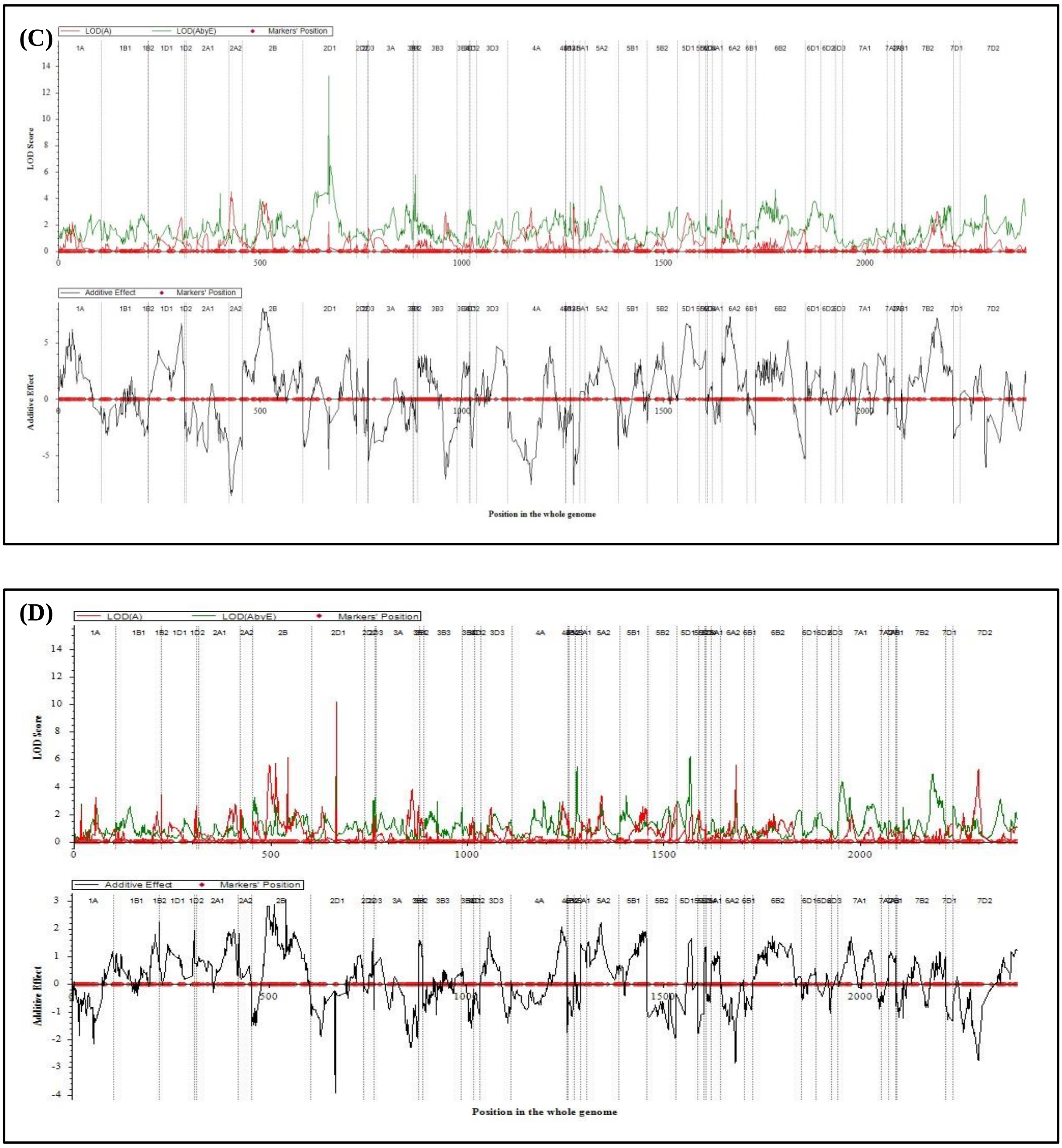
(C)
LOD(A)
LOD(AbyE)
Markers' Position
LOD Score
Additive Effect
Position in the whole genome
(D)
LOD(A)
LOD(AbyE)
Markers' Position
LOD Score
Additive Effect
Position in the whole genome

(E)

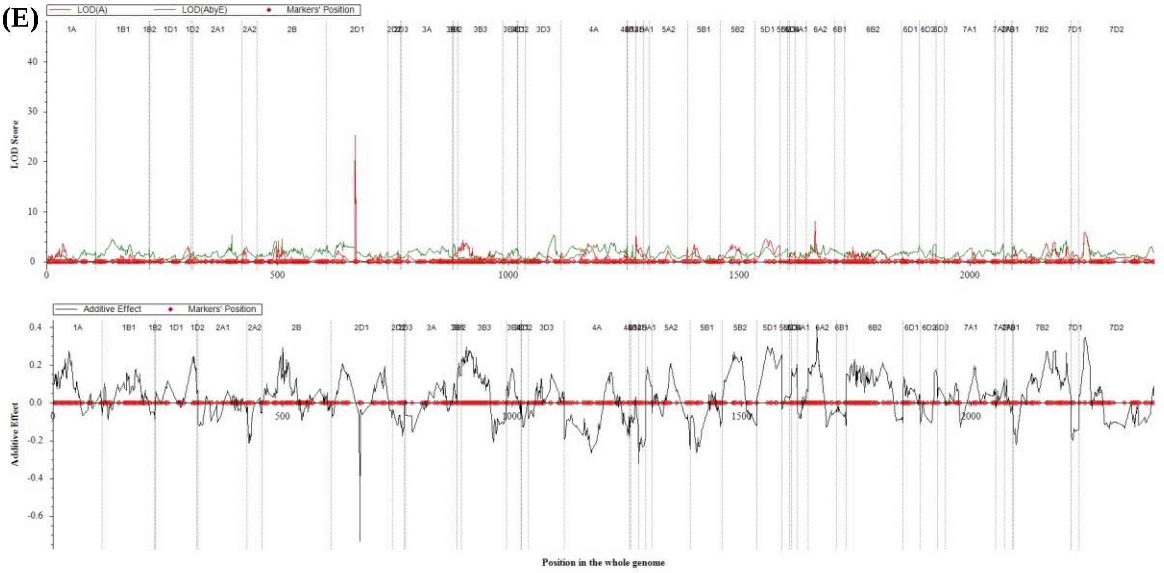
LOD(A)
LOD(AbyE)
Markers' Position
LOD Score
Additive Effect
Markers' Position
Additive Effect
Position in the whole genome

(F)

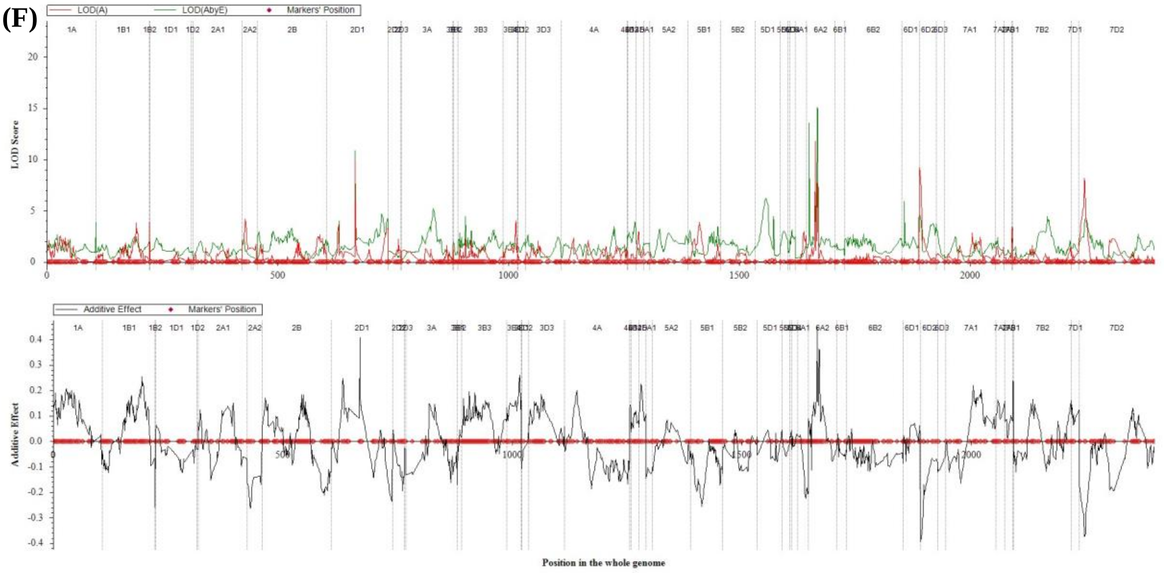
LOD(A)
LOD(AbyE)
Markers' Position
LOD Score
Additive Effect
Markers' Position
Additive Effect
Position in the whole genome

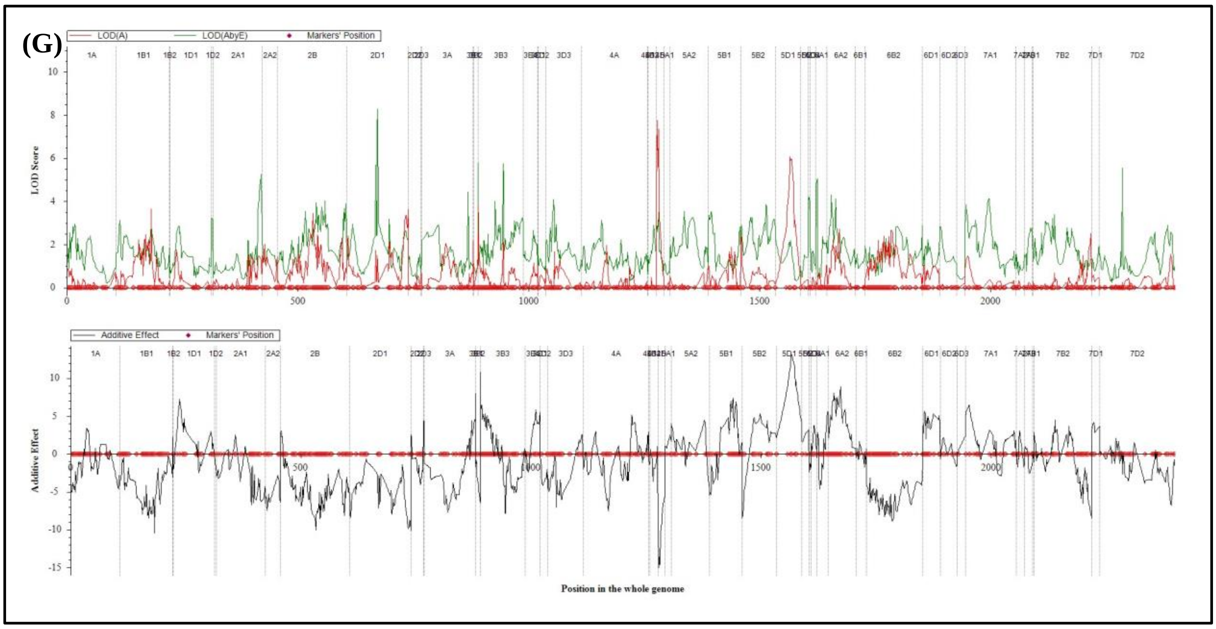
(G)
LOD(A)
LOD(AbyE)
Markers' Position
LOD Score
0
500
1000
1500
2000
Additive Effect
Markers' Position
Additive Effect
Position in the whole genome

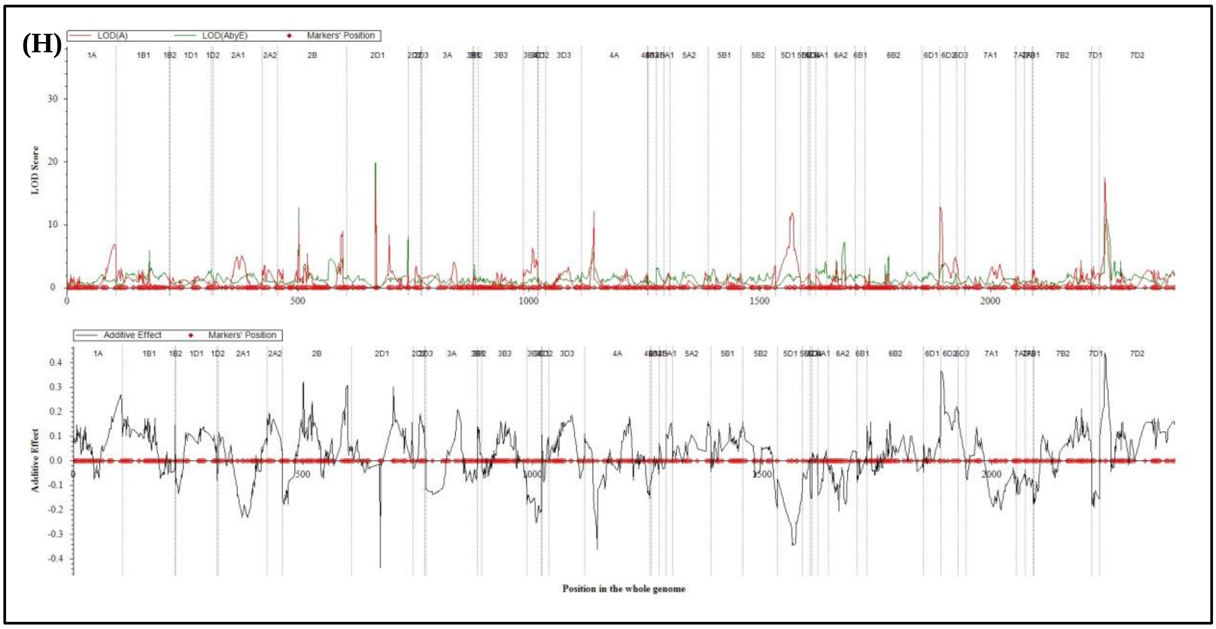
(H)
LOD(A)
LOD(AbyE)
Markers' Position
LOD Score
0
500
1000
1500
2000
Additive Effect
Markers' Position
Additive Effect
Position in the whole genome

(I)

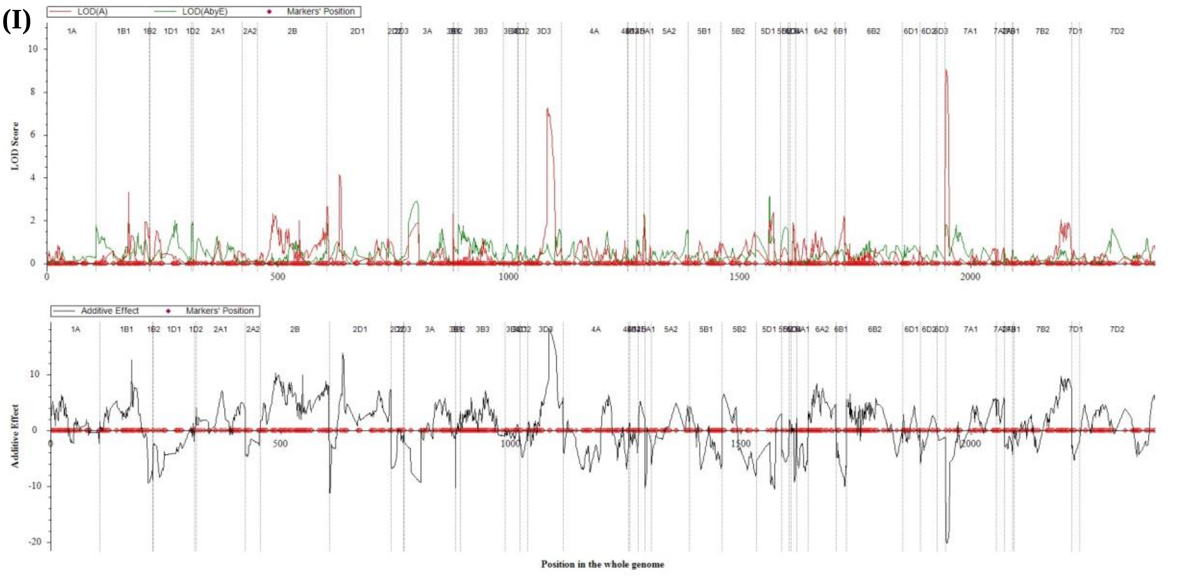

LOD(A)
LOD(AbyE)
Markers' Position
LOD Score
Additive Effect
Markers' Position
Additive Effect
Position in the whole genome

(J)

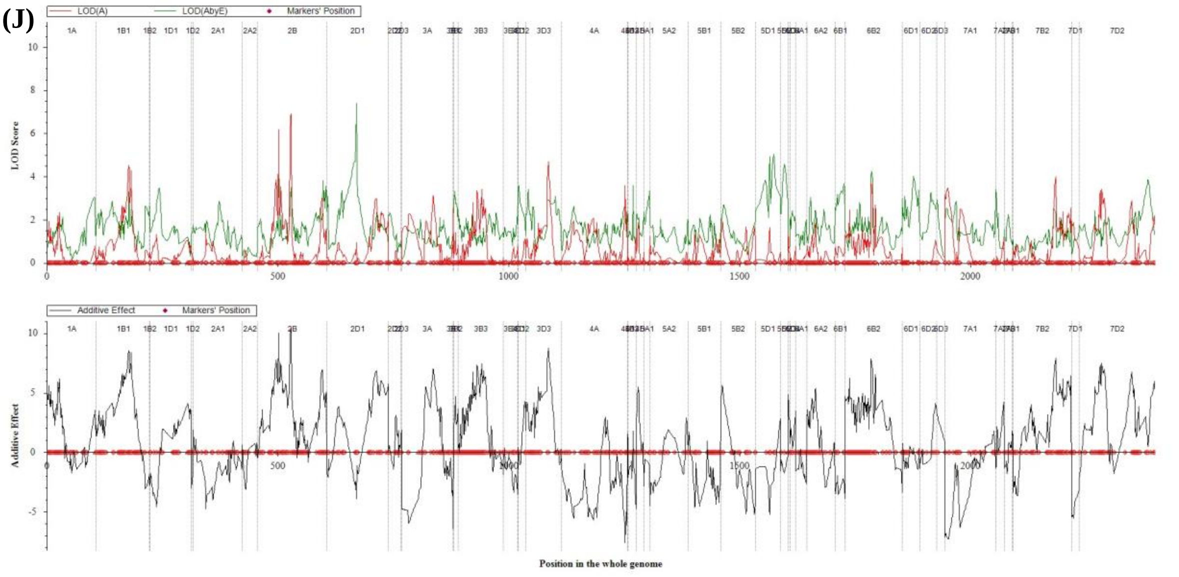

LOD(A)
LOD(AbyE)
Markers' Position
LOD Score
Additive Effect
Markers' Position
Additive Effect
Position in the whole genome

(K)

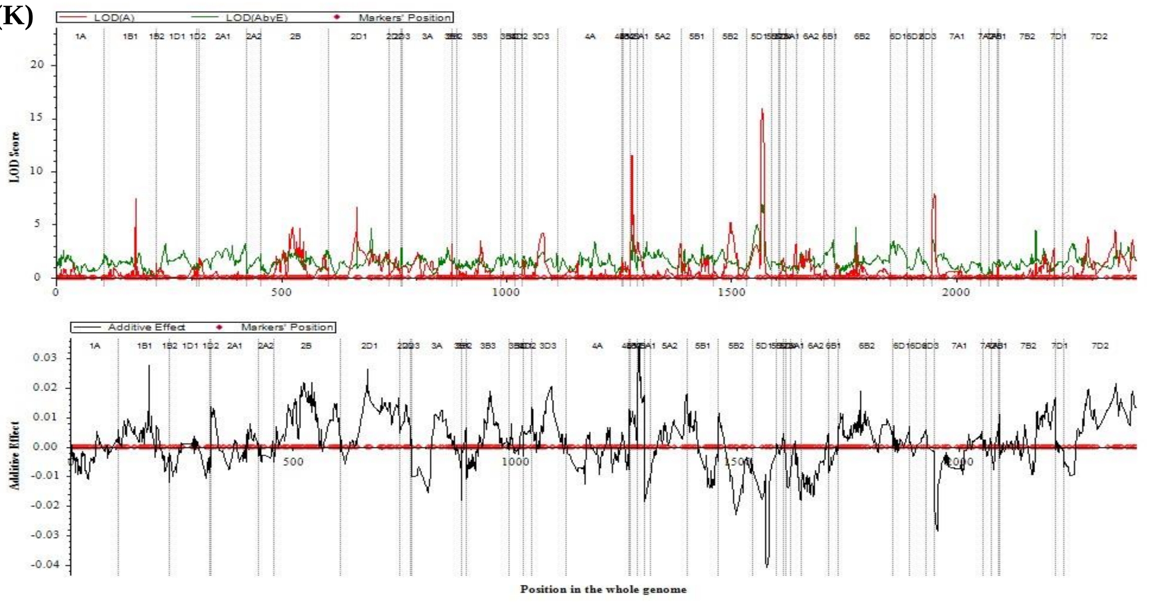
LOD(A)
LOD(AbyE)
Markers' Position
LOD Score
Additive Effect
Markers' Position
Additive Effect
Position in the whole genome

(L)

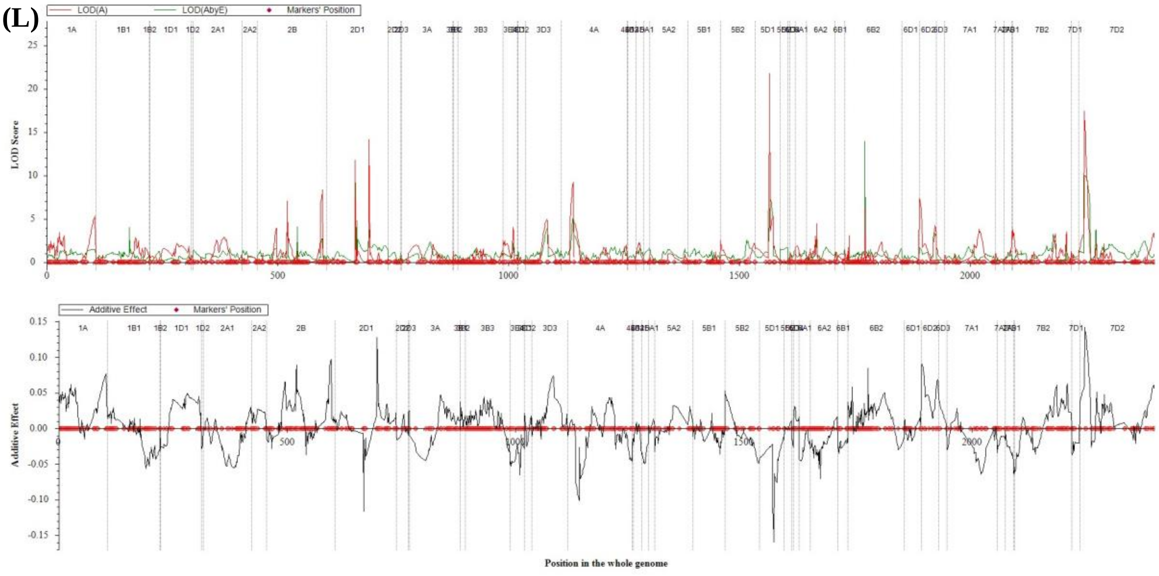
LOD(A)
LOD(AbyE)
Markers' Position
LOD Score
Additive Effect
Markers' Position
Additive Effect
Position in the whole genome

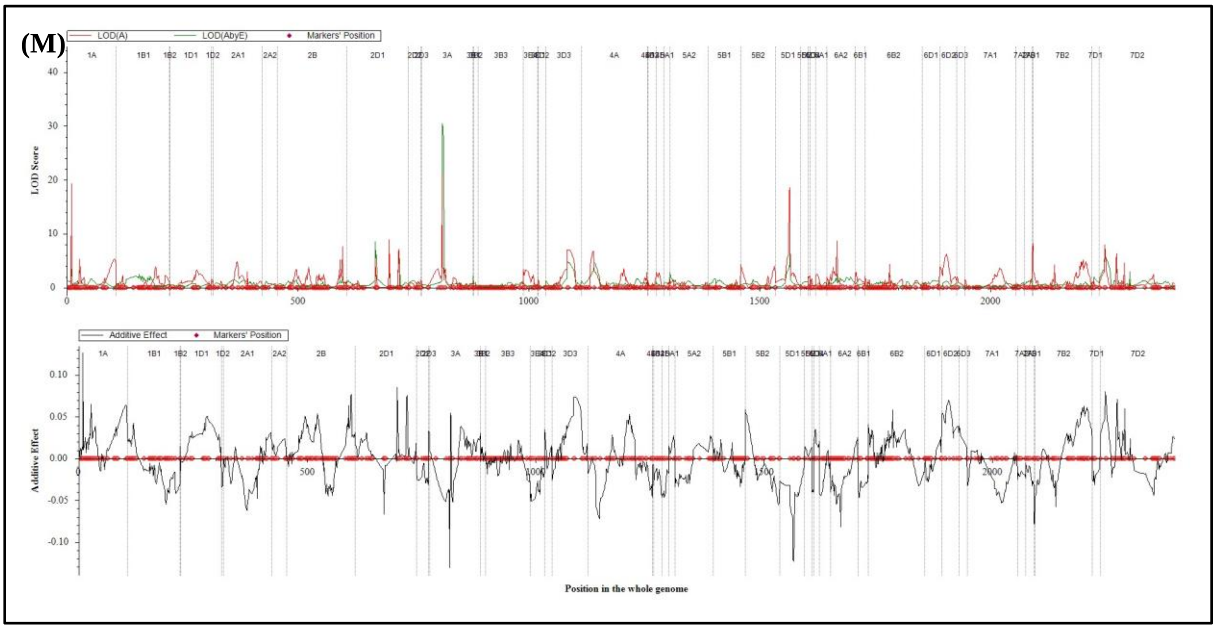
(M)
LOD(A)
LOD(AbyE)
Markers' Position
LOD Score
Additive Effect
Position in the whole genome

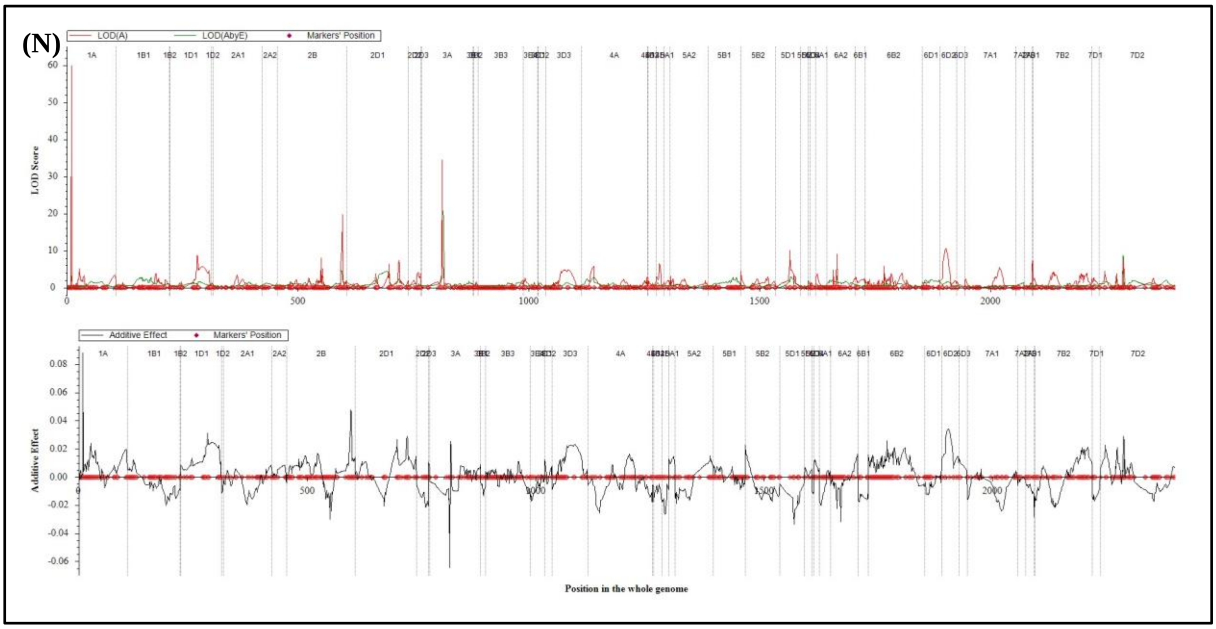
(N)
LOD(A)
LOD(AbyE)
Markers' Position
LOD Score
Additive Effect
Position in the whole genome

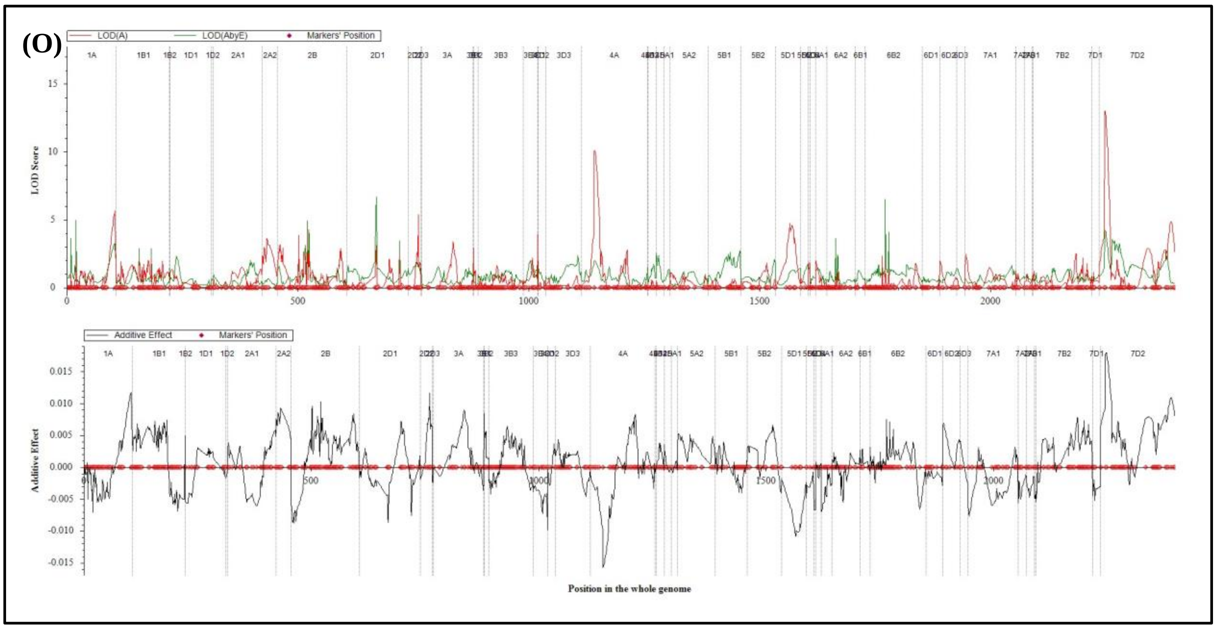
(O)
LOD(A)
LOD(AbyE)
Markers' Position
LOD Score
Additive Effect
Position in the whole genome

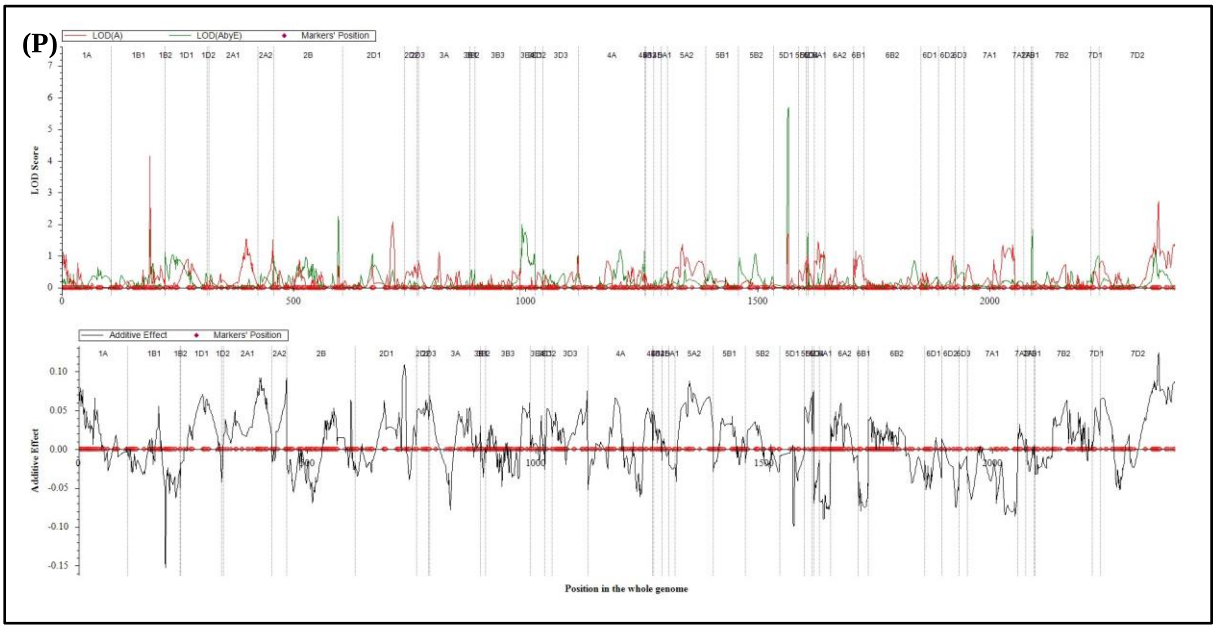
(P)
LOD(A)
LOD(AbyE)
Markers' Position
LOD Score
Additive Effect
Position in the whole genome

(Q)

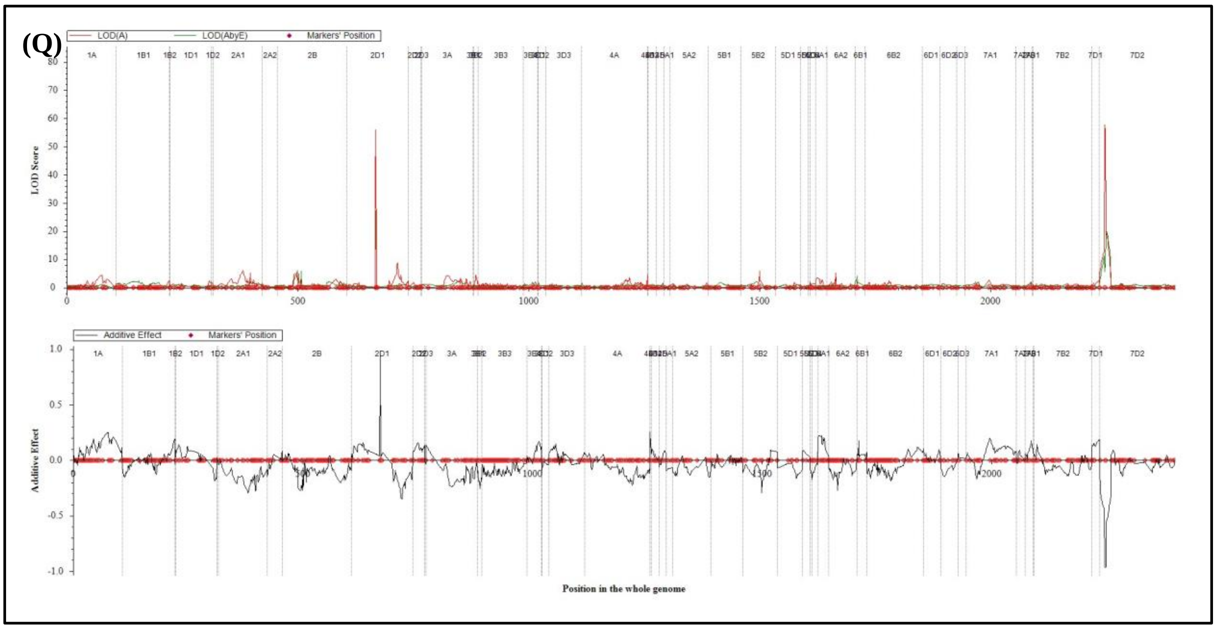

LOD(A)
LOD(AbyE)
Markers' Position
LOD Score
Additive Effect
Markers' Position
Additive Effect
Position in the whole genome

(R)

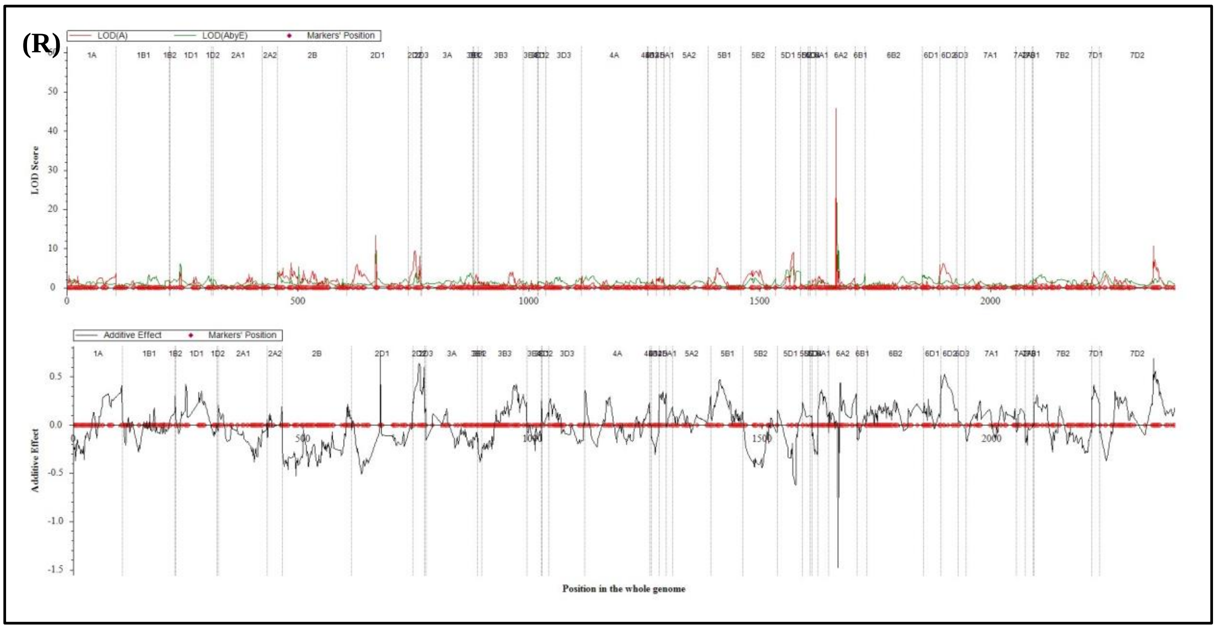

LOD(A)
LOD(AbyE)
Markers' Position
LOD Score
Additive Effect
Markers' Position
Additive Effect
Position in the whole genome

Supplemental figure S6: Whole genome significance profile of additive-by-additive epistasis effect with LOD >10 for yield, seed, and agronomy traits across individual environments. The circle with different colors represents the chromosome segments that explained by the legend on the right side. The number on each oval indicates the peak genetic position (cM) for each QTL. The red dash line shows the epistasis effect exist between markers and the number on it shows the total LOD score. Additive effect (A), additive by environment effect (AbyE), additive by additive epistasis effect (AA), additive by additive epistasis by environment effect (AAbyE) are also analyzed, the detailed information includes flank marker, genetic position, LOD scores (AA, AAbyE) and percentage of variation explained total (AA, AAbyE), effect of interacted QTL (A, AbyE, AA, AAbyE) and epistasis effects within and across mega-environments are listed in the supplement table S8. Traits: A) grain yield form whole plot (YLD, g m^{-2}); B) grain yield from biomass sample (BMYLD, g m^{-2}); C) test weight (TW, Kg m^{-3}); D) harvest index (HI, %); E) kernel $spike^{-1}$ (KPS, kernel $spike^{-1}$); F) spike m^{-2} (SPM, spike m^{-2}); G) thousand kernel weight (TKW, g); H) single head grain weight (SHGW, mg); I) single culm weight (SCW, g); J) kernel area (AREA, mm^2); K) kernel perimeter (PERI, mm); L) kernel length (KLEN, mm); M) kernel width (KWID, mm); N) heading date (HD, days); O) plant height (PH, cm).

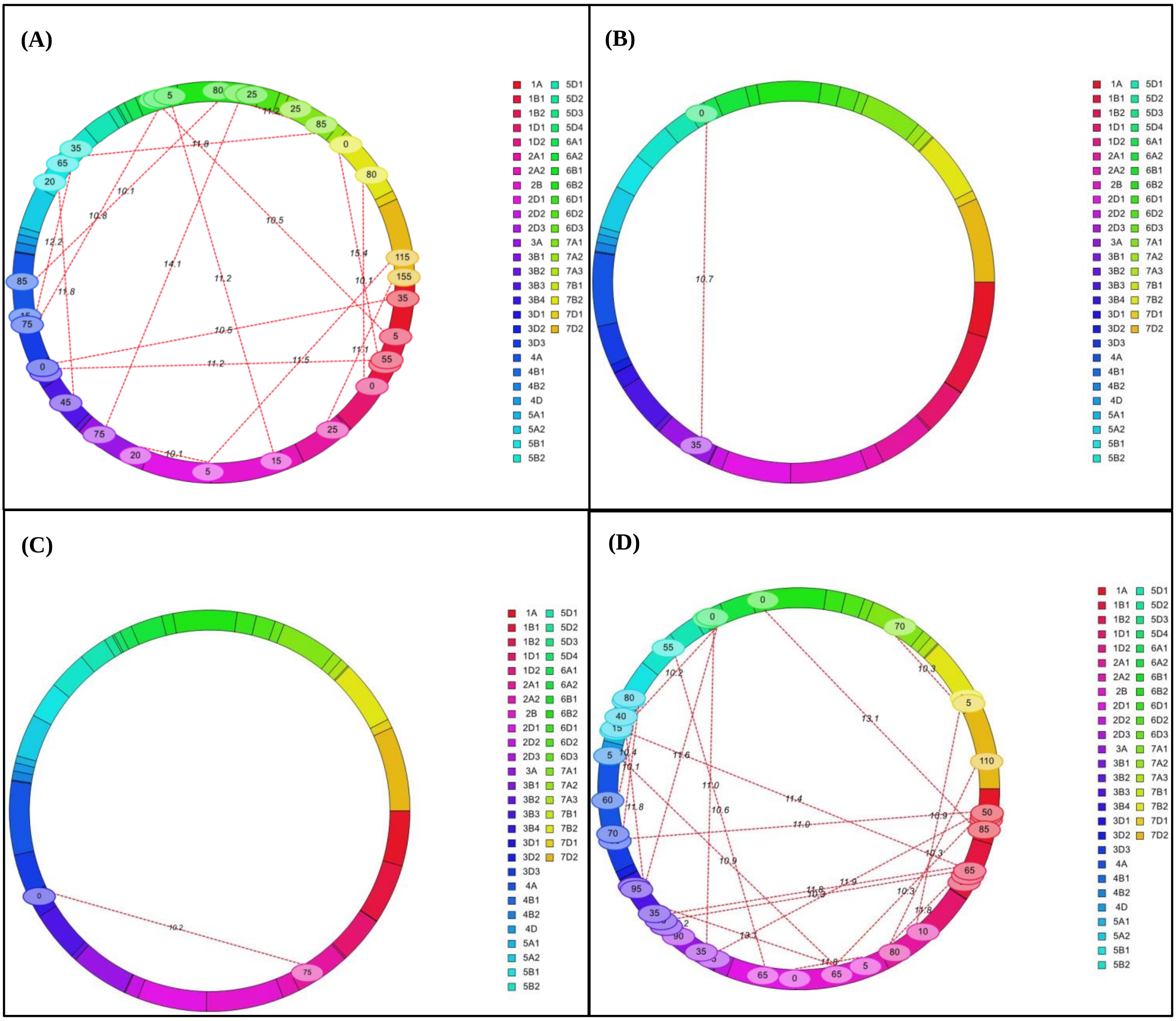
(A)
(B)
(C)
(D)
1A 1B1 1B2 1D1 1D2 2A1 2A2 2B 2D1 2D2 2D3 3A 3B1 3B2 3B3 3B4 3D1 3D2 3D3 4A 4B1 4B2 4D 5A1 5A2 5B1 5B2
5D1 5D2 5D3 5D4 6A1 6A2 6B1 6B2 6D1 6D2 6D3 7A1 7A2 7A3 7B1 7B2 7D1 7D2

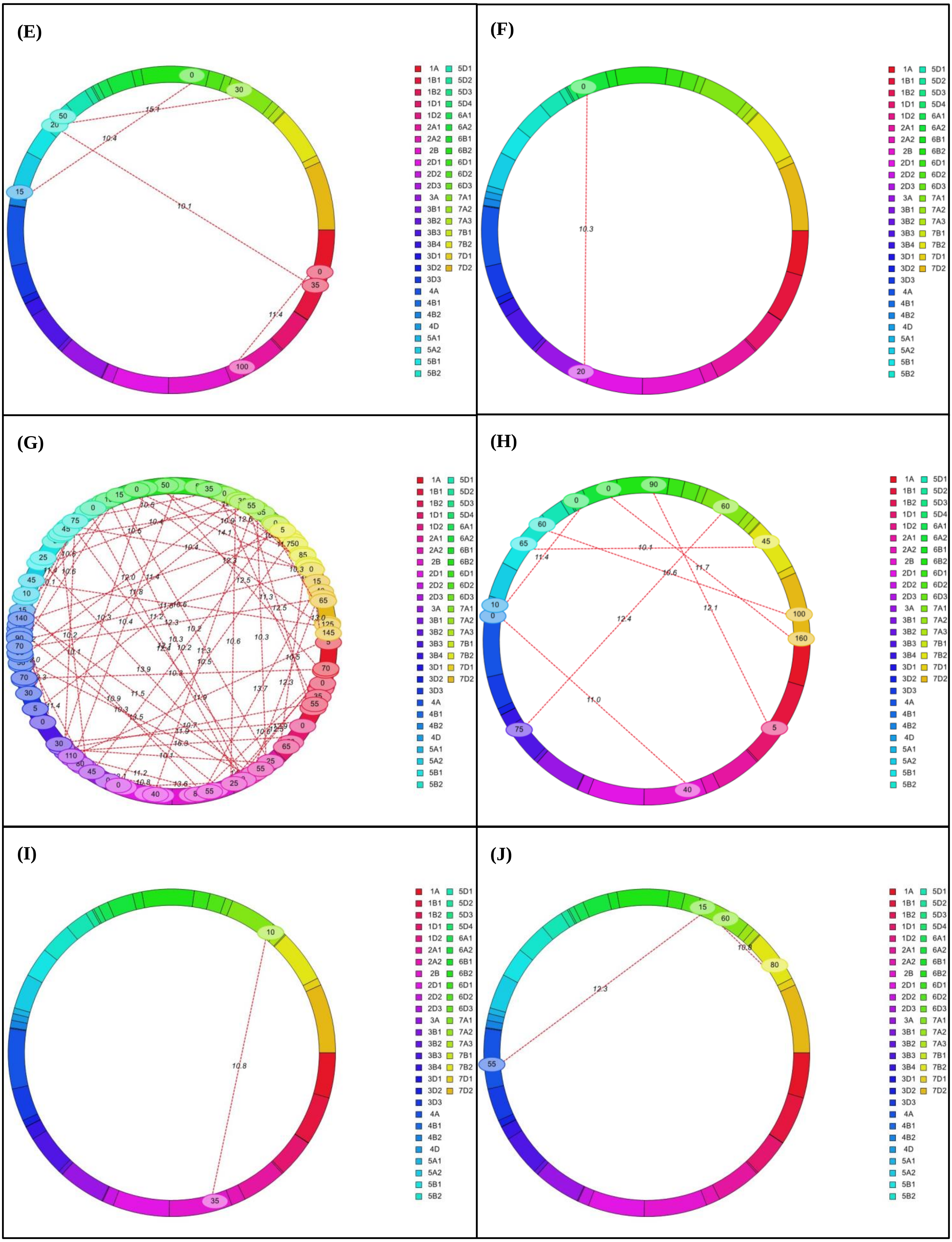
(E)
(F)
(G)
(H)
(I)
(J)

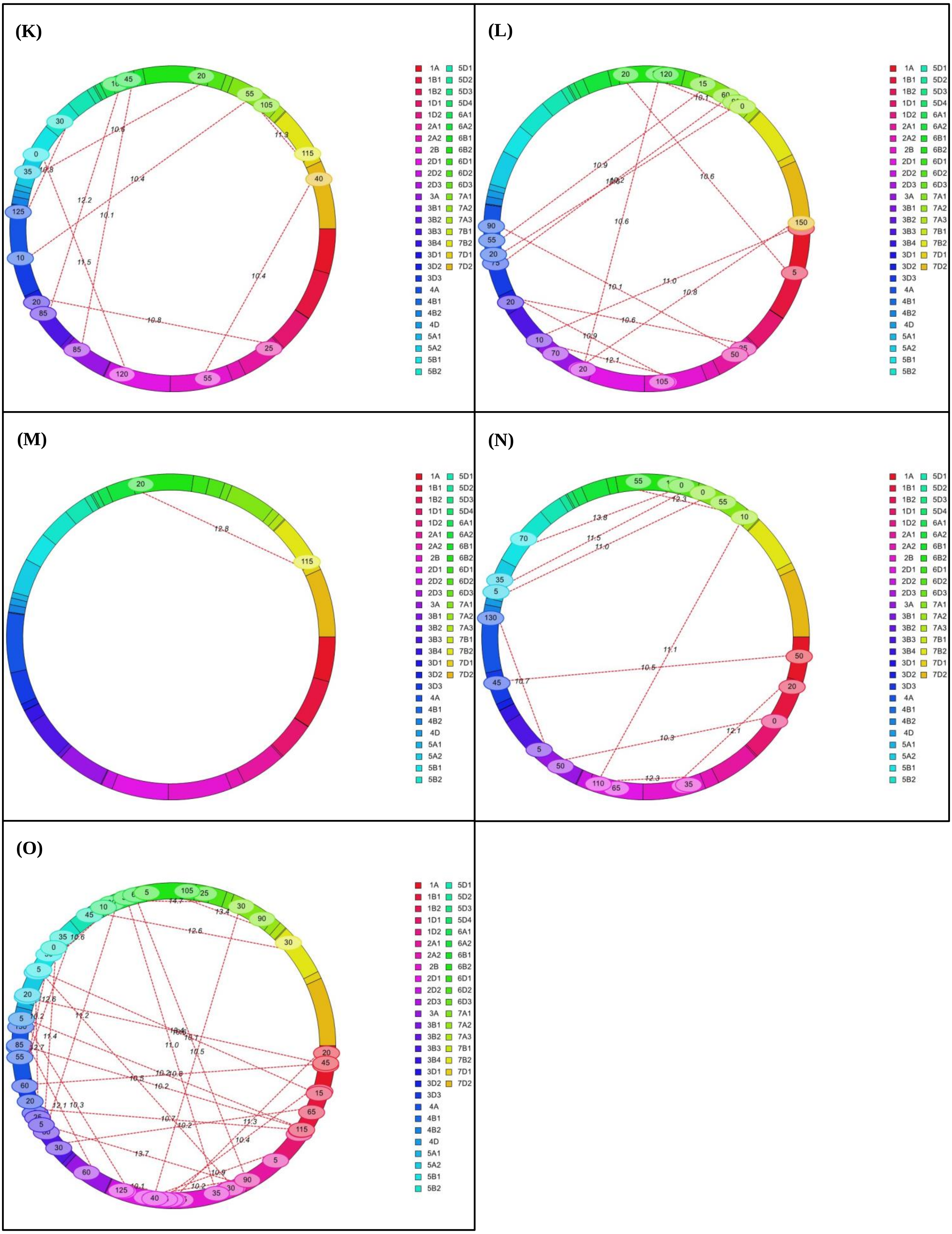
(K)
(L)
(M)
(N)
(O)

Supplemental figure S7: Haplotype analysis for major consistent QTL related to major yield traits. Traits include: A1, A2) grain yield from whole plot (YLD, g m^{-2}) for *Qyld.tamu.2D.3* (2Da) and *Qyld.tamu.2D.37* (2Db) in 17RRD and 19RRD, both with Duster alleles; B1, B2) kernel $spike^{-1}$ (KPS, kernel $spike^{-1}$) for *Qkps.tamu.2D.37* (2D) and *Qkps.tamu.6A.113* (6A) in 17BSP67 and 18BI, both with TAM 112 alleles; C1) thousand kernel weight (TKW, g) for *Qtkw.tamu.2B.62* (2Ba) and *Qtkw.tamu.2B.785* (2Bb) with TAM 112 alleles, *Qtkw.tamu.2D.37* (2D) and *Qtkw.tamu.4A.84* (4A) with Duster alleles in 17BSP100; C2) TKW for *Qtkw.tamu.2D.37* (2D) with Duster alleles and *Qtkw.tamu.7D.66* (7D) with TAM 112 alleles in 18BI; D1) kernel width (KWID, mm) for *Qkwid.tamu.2D.37* (2D) with Duster alleles and *Qkwid.tamu.7D.68* (6D) with TAM 112 alleles in 18BMS; E1, E2, E3) kernel length (KLEN, mm) for *Qklen.tamu.1A.325* (1A), *Qklen.tamu.2B.781* (2B), and *Qklen.tamu.7D.157* (7D) with TAM 112 allele, and *Qklen.tamu.3A.664* (3A) with Duster allele in 18BSP100, 19BSP100, and 19BMS. Environment abbreviations: Texas A&M AgriLife Research stations in Bushland, TX, irrigated land in 2018 (18BI), Bushland middle school dryland in 2018, 2019 (18BMS, 19BMS). Bushland south pivot irrigated land in 2017 with 67% and 100% irrigation levels (17BSP67, 17BSP100), in 2018, 2019 with 100% irrigation level (18BSP100, 19BSP100). Red River Research and Demonstration Farm in 2017, 2019 (17RRD, 19RRD).

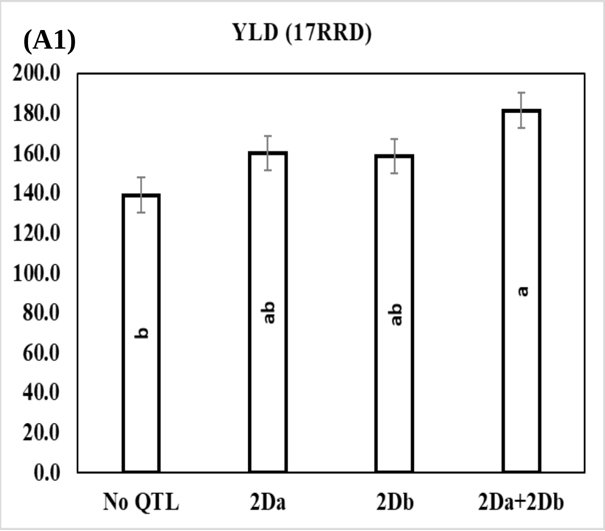

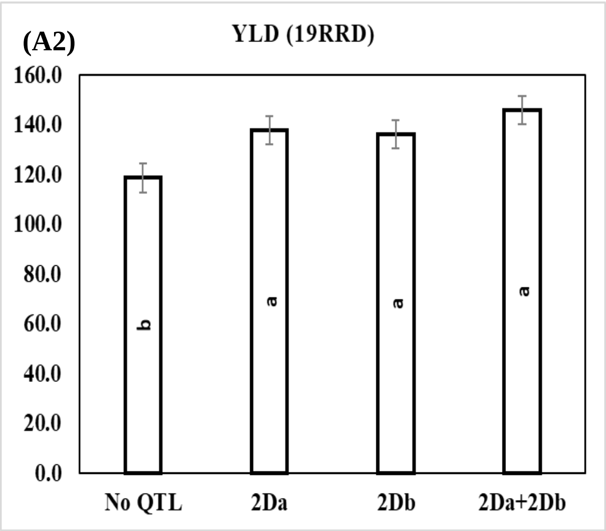

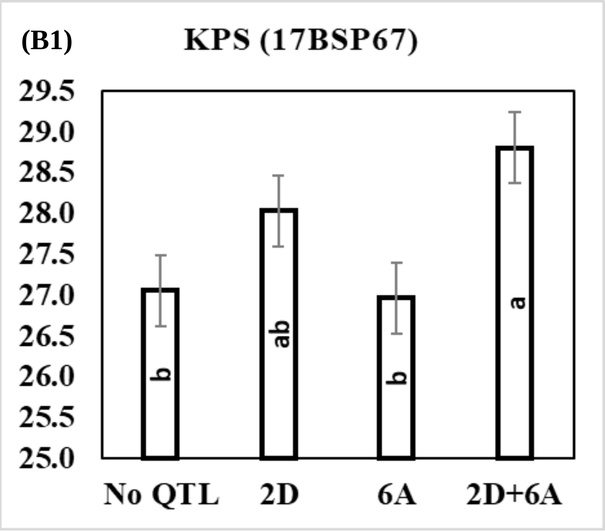

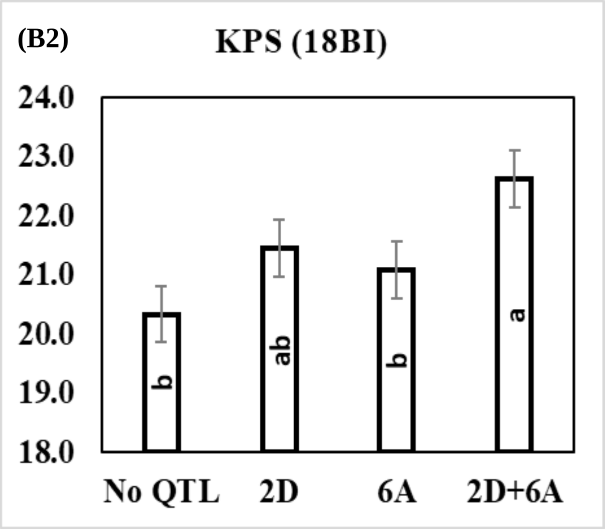

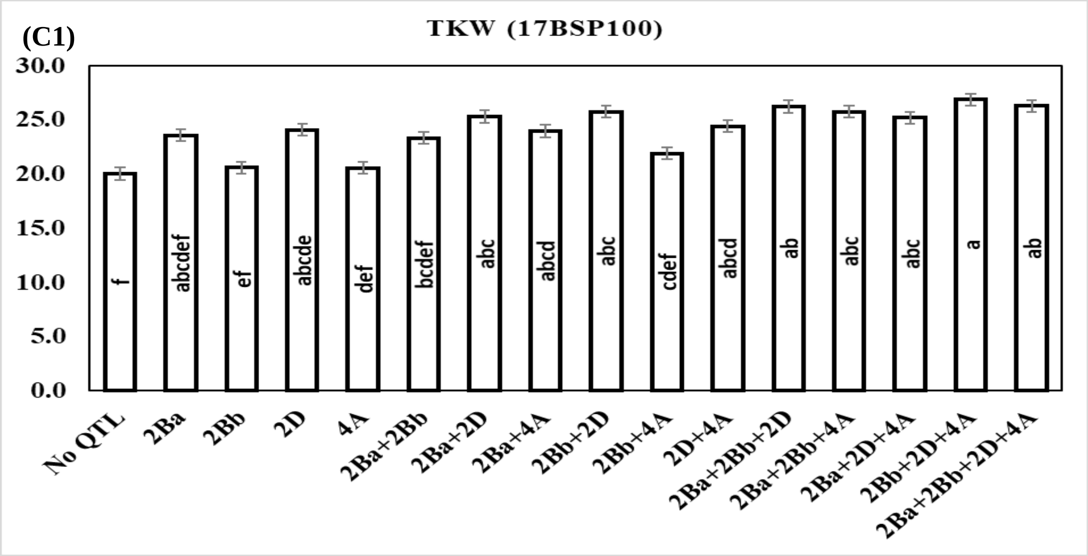
(C1)
TKW (17BSP100)
30.0
25.0
20.0
15.0
10.0
5.0
0.0
f
abcdef
ef
abcde
def
bcdef
abc
abcd
abc
cdef
abcd
ab
abc
abc
a
ab
No QTL
2Ba
2Bb
2D
4A
2Ba+2Bb
2Ba+2D
2Ba+4A
2Bb+2D
2Bb+4A
2D+4A
2Ba+2Bb+2D
2Ba+2Bb+4A
2Ba+2D+4A
2Bb+2D+4A
2Ba+2Bb+2D+4A

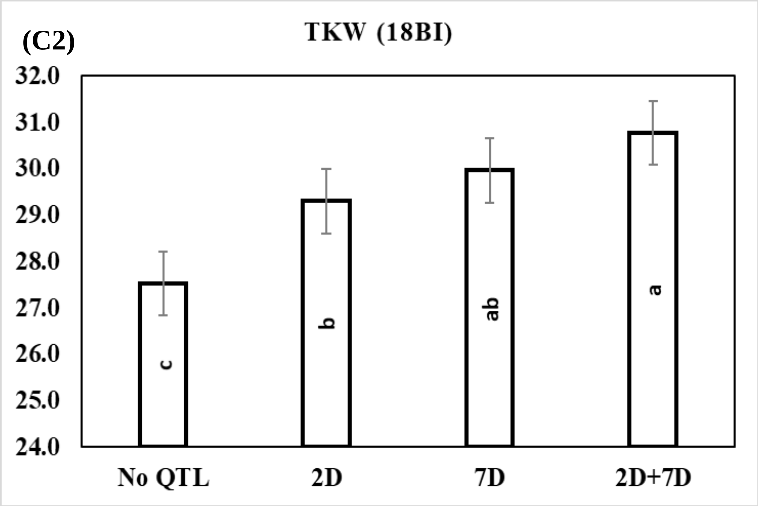
(C2)
TKW (18BI)
32.0
31.0
30.0
29.0
28.0
27.0
26.0
25.0
24.0
c
b
ab
a
No QTL
2D
7D
2D+7D

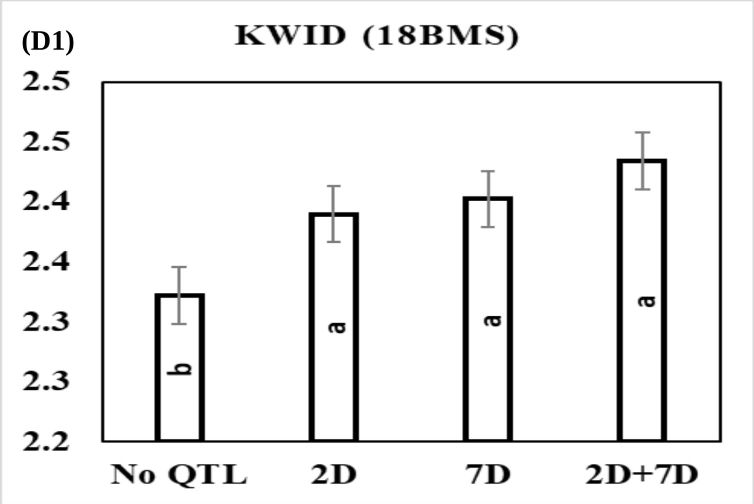
(D1)
KWID (18BMS)
2.5
2.5
2.4
2.4
2.3
2.3
2.2
b
a
a
a
No QTL
2D
7D
2D+7D

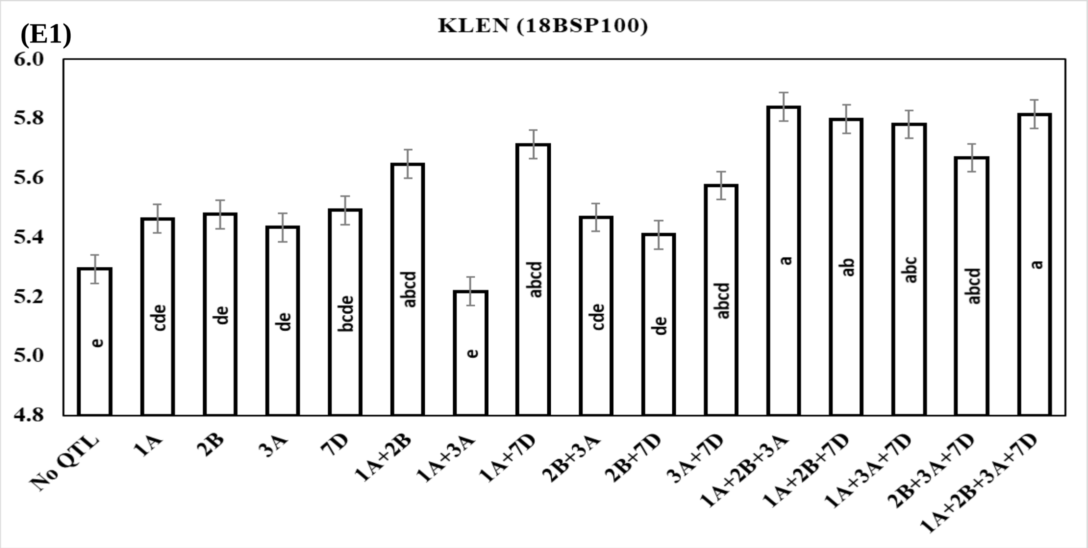
(E1)
KLEN (18BSP100)
6.0
5.8
5.6
5.4
5.2
5.0
4.8
e
cde
de
de
bcde
abcd
e
abcd
cde
de
abcd
a
ab
abc
abcd
a
No QTL
1A
2B
3A
7D
1A+2B
1A+3A
1A+7D
2B+3A
2B+7D
3A+7D
1A+2B+3A
1A+2B+7D
1A+3A+7D
2B+3A+7D
1A+2B+3A+7D

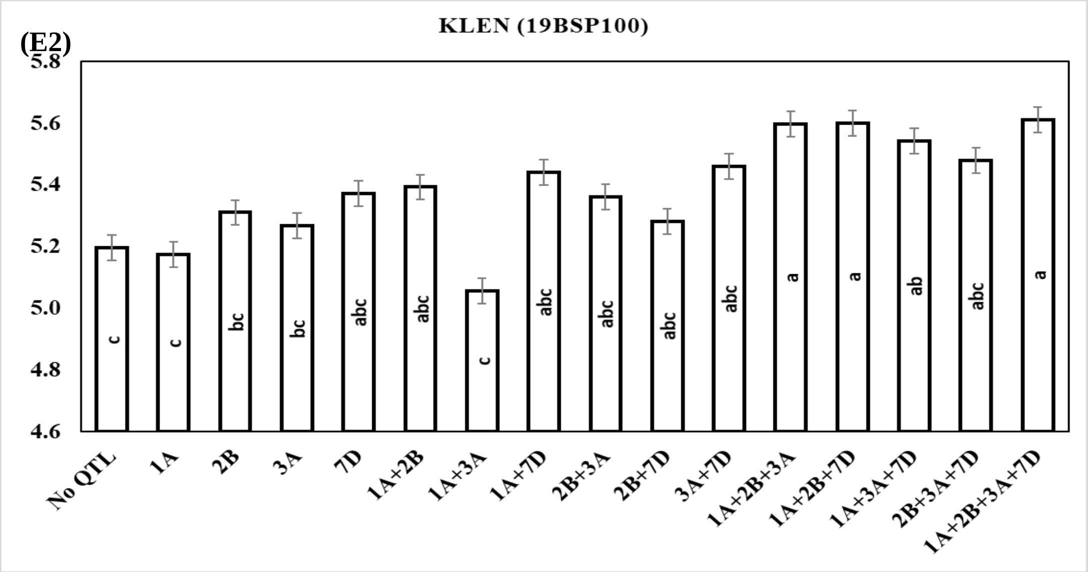
(E2)
KLEN (19BSP100)
5.8
5.6
5.4
5.2
5.0
4.8
4.6
c
c
bc
bc
abc
abc
c
abc
abc
abc
abc
a
a
ab
abc
a
No QTL
1A
2B
3A
7D
1A+2B
1A+3A
1A+7D
2B+3A
2B+7D
3A+7D
1A+2B+3A
1A+2B+7D
1A+3A+7D
2B+3A+7D
1A+2B+3A+7D

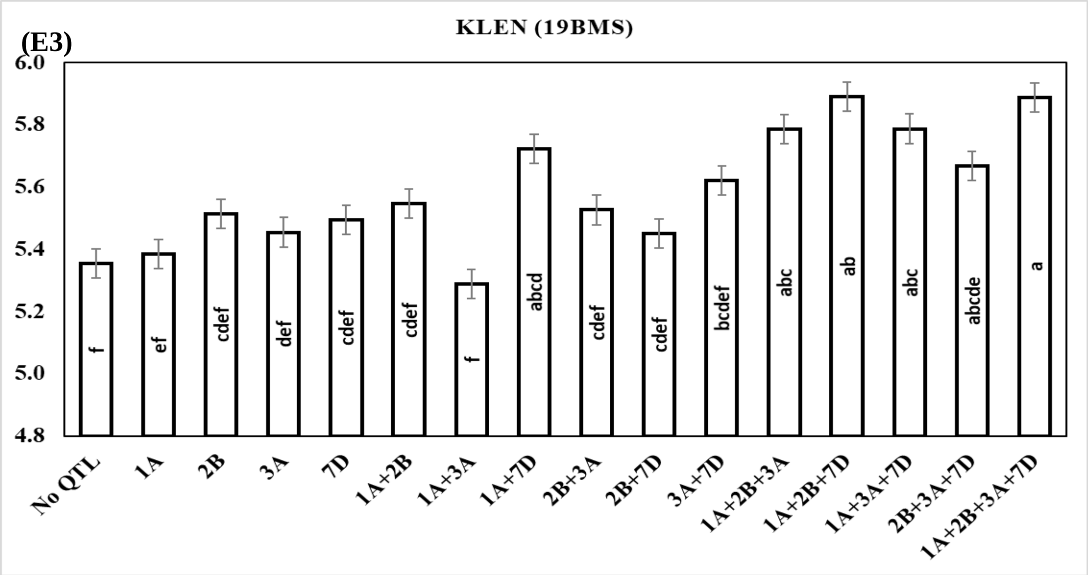
(E3)
KLEN (19BMS)
6.0
5.8
5.6
5.4
5.2
5.0
4.8
f
ef
cdef
def
cdef
cdef
f
abcd
cdef
cdef
bcdef
abc
ab
abc
abcde
a
No QTL
1A
2B
3A
7D
1A+2B
1A+3A
1A+7D
2B+3A
2B+7D
3A+7D
1A+2B+3A
1A+2B+7D
1A+3A+7D
2B+3A+7D
1A+2B+3A+7D
